# Efficacy of therapies for intermediate-stage hepatocellular carcinoma: systematic review and network meta-analysis

**DOI:** 10.3389/fimmu.2025.1577614

**Published:** 2025-07-09

**Authors:** Shaoqi Zong, Yifan Yang, Zifei Yin, Lanyun Feng, Kun Wang, Hao Chen, Zhen Chen, Zhiqiang Meng, Yongqiang Hua

**Affiliations:** ^1^ Department of Integrative Oncology, Fudan University Shanghai Cancer Center, Shanghai, China; ^2^ Department of Minimally Invasive Therapy Center, Fudan University Shanghai Cancer Center, Shanghai, China; ^3^ Department of Oncology, Shanghai Medical College, Fudan University, Shanghai, China; ^4^ School of Traditional Chinese Medicine, Naval Medical University, Shanghai, China

**Keywords:** intermediate stage, hepatocellular carcinoma, TACE, efficacy, network meta-analysis

## Abstract

**Background:**

Transarterial chemoembolization (TACE) is recommended for intermediate-stage hepatocellular carcinoma (HCC). However, several therapies have shown better efficacy than TACE, meaning that the optimal therapy is unclear. We addressed this uncertainty using network meta-analysis (NMA).

**Methods:**

A literature review was performed up to March 15, 2024. Efficacy was evaluated using overall survival (OS) and progression-free survival (PFS). The hazard ratios (HRs) and 95% confidence intervals (CIs) were extracted from the Kaplan–Meier curves. A random-effects NMA was conducted, and subgroup analysis was performed according to the tumor number, tumor size, viral etiology, and alpha fetoprotein (AFP) level. The efficacy of the different therapies was ranked based on the *P*-score.

**Results:**

A total of 38 studies, 10,972 patients, and 13 therapeutic regimens were eligible. Seven therapies showed OS benefit over TACE, including TACE plus microwave ablation (MWA) (HR = 0.24, 95%CI = 0.06–0.91), TACE plus liver resection (HR = 0.35, 95%CI = 0.22–0.57), liver resection plus RFA (HR =0.49,95%CI=0.35-0.70), TACE plus immune checkpoint inhibitors (ICIs) plus tyrosine kinase inhibitors (TKIs) (HR = 0.51, 95%CI = 0.27–0.95), liver resection (HR = 0.54, 95%CI = 0.45–0.65), and TACE plus radiofrequency ablation (RFA) (HR = 0.57, 95%CI = 0.36–0.93). However, no therapies improved the PFS better than TACE alone. Subgroup analysis indicated that liver resection plus TACE showed the best OS for patients with hepatitis B virus (HBV) infection.

**Conclusions:**

Seven therapies showed better efficacy than TACE alone for particular patients with intermediate-stage HCC.

**Systematic review registration:**

https://www.crd.york.ac.uk/, PROSPERO CRD42023459740.

## Introduction

Globally, primary liver cancer (PLC) is the sixth most frequent malignancy and the third leading cause of cancer-related death. In particular, there were approximately 860,000 new cases of PLC in 2023 (1). Hepatocellular carcinoma (HCC) accounts for nearly 80% of PLC (2). Hepatitis virus infection, aflatoxin, and metabolic dysfunction are the main risk factors of HCC. Notably, due to the lack of obvious symptoms in the early stage, nearly 80% of patients with HCC are diagnosed in the middle–advanced stage (3). Exacerbating this problem is the high resistance of some patients in the intermediate-advanced stage to current therapies and the limited survival benefits.

Presently, the Barcelona Clinic Liver Cancer (BCLC) classification, which is both a treatment strategy and a staging system, has been externally confirmed and is widely endorsed by a series of liver disease associations, including the European Association for the Study of Liver (EASL) and the American Association for the Study of Liver Diseases (AASLD) (3–5). Patients in the BCLC-B stage (intermediate stage) are defined as those with multifocal tumors with preserved liver function, good performance status, and without macrovascular invasion and extrahepatic spread (6). Therefore, intermediate-stage HCC is characterized by a high heterogeneity with extensive tumor number, tumor size, and different liver function.

Transarterial chemoembolization (TACE) is recommended as the standard therapy for patients with BCLC-B (7). The anticancer mechanism of TACE involves not only blocking the blood supply of the embolization area but also directly killing tumor cells through chemotherapy drugs. However, the median overall survival (OS) of patients treated with TACE ranges from 14 to 45 months due to the high heterogeneity of both the patient population and the TACE technique (8, 9). Partial patients are unsuitable for or are refractory to TACE (10). In addition, a considerable number of patients suffer from serious post-embolization syndromes (PES) after TACE, leading to a reduction in treatment compliance (11). Furthermore, only half of patients with HCC could benefit from a single TACE, in particular patients with large and/or multifocal tumors, which might be caused by the incomplete necrosis of the target lesions after a single TACE (12). Therefore, this procedure is repeatedly performed several times in the clinic. However, repeated TACE is usually accompanied with hepatic injury and induces the formation of an ischemic/hypoxia microenvironment, subsequently activating the hypoxia-induced factor 1 alpha (HIF-1α) pathway and modulating angiogenesis, tumor invasion, and metastasis (13, 14). Therefore, the efficacy of a single TACE in the treatment of patients with BCLC-B stage HCC is unsatisfactory. Accordingly, several randomized controlled trials (RCTs) have been conducted to compare the efficacy of TACE plus molecular targeted therapy (TACE+MTT) with that of TACE alone (15–17). Regrettably, these trials did not achieve positive results.

Presently, the efficacy and the safety of immune checkpoint inhibitors (ICIs) in the treatment of HCC have been confirmed. The IMbrave150 trial confirmed that atezolizumab (ICI) plus bevacizumab significantly prolonged the OS and PFS compared with sorafenib in patients with unresected HCC (18). Subgroup analysis also showed a positive trend in patients with BCLC-B stage HCC (19). Interestingly, Pinato et al. found that TACE could induce immunogenic cell death and release tumor antigens, which may enhance the efficacy of ICIs (20). The CHANCE001 trial, a multicenter retrospective cohort study in China, demonstrated that TACE with ICIs plus MTT showed better OS and PFS than TACE alone in patients with advanced HCC; however, the trial did not improve the OS and PFS in patients with intermediate-stage HCC (21). In addition, the EMERALD-1 trial, a global three-phase study on patients with unresected HCC, showed a significant benefit in PFS using TACE combined with durvalumab (ICI) plus bevacizumab compared with TACE alone. Notably, this trial showed positive results in patients with BCLC-B stage HCC (22). Recently, the LEAP-012 trial evaluated the efficacy of TACE plus lenvatinib plus pembrolizumab *vs*. TACE alone in patients with intermediate-stage HCC. The PFS was significantly prolonged in the triple therapy group compared with the monotherapy group. At the same time, the OS did not show positive results (23). In addition, an RCT found that partial resection showed better OS than TACE in patients with resectable multiple HCC out of the Milan criteria (24). Furthermore, several therapies, including TACE plus thermal ablation, TACE plus liver resection, liver resection plus TACE, and liver resection plus ablation, showed therapeutic benefits in patients with BCLC-B stage HCC (25–27). However, most of these clinical trials have a small sample size and are retrospective in nature.

As described above, there exists a series of potential therapies for patients with intermediate-stage HCC. However, due to the lack of direct comparisons, the optimal therapy is controversial. Therefore, we conducted a network meta-analysis (NMA) to indirectly compare the efficacy of these therapies.

## Materials and methods

We searched and extracted relative data according to the latest Preferred Reporting Items for Systematic Reviews and Meta-analysis (PRISMA) and Assessing the Methodological Quality of Systematic Reviews (AMSTAR) guidelines (28). The results are shown in **Supplementary Tables S1**, **S2**. This study is registered in the PROSPERO database (CRD42023459740).

### Search strategy and selection criteria

We searched relevant databases, including PubMed, Embase, Web of Science, and the Cochrane Library. In addition, the references of the retrieved articles and meta-analyses were manually searched. The last search date was up to March 15, 2024. The relative search items for each database are shown in **Supplementary Table S3**.

### Participants

BCLC-B stage HCC was defined according to the following criteria: 1) two or three nodules and with at least one tumor larger than 3 cm; 2) four or more tumor nodules, any size; 3) without the presentation of extrahepatic metastases; 4) without tumor thrombus in the portal vein or other major vascular structures; 6) Child–Pugh (Child–Turcotte–Pugh, CTP) A–B class; and additionlly, samle size ≥25.

### Interventions

Interventions included the therapies for intermediate-stage HCC.

### Comparator

TACE was used as the reference.

### Outcomes

The primary endpoint was OS. The secondary outcome was PFS.

### Study selection

Firstly, two authors independently read and reviewed the titles and abstracts according to the search strategy. On this basis, the selected studies were further confirmed by downloading and reviewing the full texts. Subsequently, relative data were extracted into a pre-designed Excel sheet, including the PMID number; author; publication year; sample size; follow-up duration; study design; efficacy outcomes; and clinical parameters such as age, sex, tumor size, tumor number, hepatitis B virus (HBV)/hepatitis C virus (HCV) infection status, Child–Pugh score, and alpha fetoprotein (AFP) level ≥400 ng/ml.

### Data extraction

The hazard ratios (HRs) and relative 95% confidence intervals (CIs) of the individual study were pooled for the survival data. If the HR and 95% CIs were reported, these data were directly extracted for further analysis; otherwise, the HR and 95%CIs were extracted from the survival curves using the method reported by Parmar et al. (29).

### Risk of bias assessment

Two authors independently evaluated the risk of bias (RoB) of the included studies. The assessment tools used were based on the study design. For RCTs, the Risk of Bias 2 (RoB2) tool was used (30). For observational studies, the Risk of Bias in Non-randomized Studies of Interventions (ROBINS-I) was used. Discrepancies were resolved through discussion with another author.

### Design-adjusted analysis

Considering the potential confounding of non-randomized studies (NRS), the inclusion of these studies in the NMA may influence the transitivity and consistency assumed by the method. To minimize bias, a design-adjusted analysis was performed to combine both randomized and non-randomized evidence in the NMA (31). As described above, ROBINS-I was used to evaluate the RoB of the NRS. Studies with a higher RoB were assigned a lower weight, while studies with a lower RoB were assigned a higher weight. In detail, for observational studies with low, moderate, and high RoB, weights of 25%, 50%, and 75%, respectively, were assigned. For RCTs, the assigned weight was 1.

### Standard meta-analysis

STATA (version 12.0; Stata Corp, College Station, TX, USA) was used for pairwise meta-analysis. Firstly, statistical heterogeneity was evaluated using the *I* ^2^ value. An *I* ^2^ ≥ 50% meant that there exists significant heterogeneity; therefore, the random-effects model was used. Otherwise, the fixed-effects model was utilized for pooling the HR, relative risk (RR), and relative 95%CIs. Egger’s regression test was conducted to evaluate publication bias, and a funnel plot was used for visual assessment.

### Network meta-analysis

A frequentist model NMA was performed using the “netmeta” package in R software (version 4.3.1; R Foundation for Statistical Computing, Vienna, Austria). A random-effects NMA model was used, and the network plots were drawn accordingly. For the evaluation and ranking of the efficacy of each treatment, the *P*-scores of each therapy were calculated and accordingly ranked. In terms of efficacy, a *P*-score of “0” denotes worst relative therapy, while “1” indicates that the treatment therapy is the best, which is contrary to the terms of adverse events.

### Inconsistency assessment

The consistency of the NMA is crucial for the evaluation of the stability of the transitivity assumption. Firstly, the back-calculation method was used to assess the existence of local inconsistency. In addition, a design-by-treatment interaction was determined to evaluate the global inconsistency of the model (32). The “netmeta” package in R was used to accomplish the above analysis.

### Subgroup analysis

For the primary outcomes, subgroup analysis was conducted to explore the source of heterogeneity according to the following parameters: AFP level, Child–Pugh class, tumor size, tumor number, HCV and HBV infection status, and sample size. The median value of the continuous variable was calculated for further subgroup analysis.

### Credibility of evidence

The evidence credibility of the NMA results was evaluated using CINeMA (http://cinema.ispm.ch/) (33), which is composed of six domains: within-study bias, across-study bias, indirectness, heterogeneity, imprecision, and incoherence. The degree of evidence of the NMA results was summarized based on these six domains.

## Results

### Study selection and characteristics

**Figure 1:**
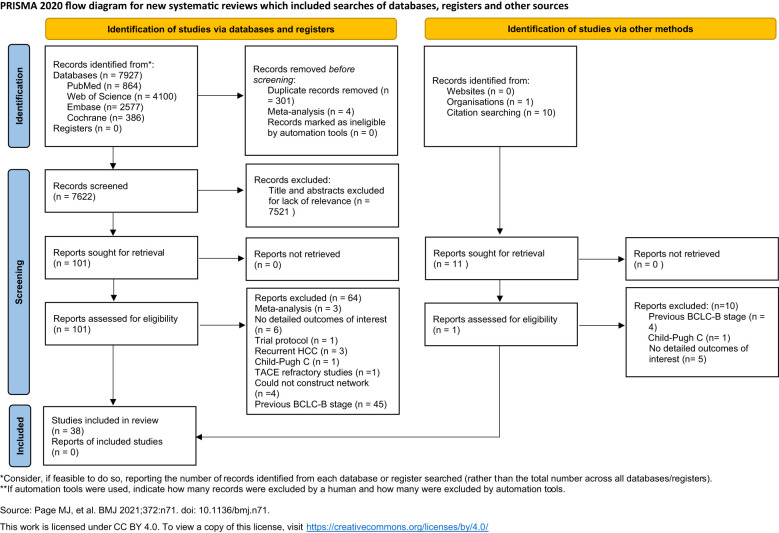
A PRISMA flow diagram of the literature screening and selection process.

Based on the search strategy, a total of 7,927 potential citations were identified. Accordingly, 301 studies with duplicate records and four meta-analyses were excluded. A total of 7,521 studies were excluded after reading the titles and abstracts. One article was excluded due to the inclusion of Child–Pugh class C patients (34), while three studies were excluded due to the enrolled participants not fulfilling the criteria: three had recurrent HCC (35–37) and one was refractory to TACE (38). There were 40 studies that reported data on OS (15, 17, 18, 21, 24, 26, 27, 39–71) and 12 studies that reported data on PFS (15, 18, 21, 22, 45, 46, 50, 52, 54, 60, 71, 72). Four studies were excluded due to the therapies not being able to form a network (18, 45, 46, 65). Finally, a total of 38 studies were found eligible for inclusion in this systematic review. The flowchart of the included studies is presented in **Figure 1**. Detailed characteristics of the included studies are shown in **Table 1**.

**Table 1:** Baseline characteristics of included studies.

Study (name)	Study design	Arm	No. of patients	Sex (Male)	Median age (Years)	CTP class A/B (%)	HBV (%)	HCV (%)	No. tumor (≥3)	Tumor size (cm)	OS (Median)	Out-comes
Ke 2014 (55) (China)	Retro	LR	53	93%	47	NA	98	NA	NA	8	35	OS
		TACE	53	93%	47	NA	93	NA	NA	9	20	OS
Akarapatima 2022 (64)	Retro	TACE	92	74%	59 (mean)	NA	40	29	NA	4.9	21	OS
(Thailand)		BSC	26	69%	61 (mean)	NA	50	4	NA	7.0	8	OS
Wang 2023 (37)	Retro	LR+TACE	105	95%	51	NA	88	NA	22.9	NA	NA	OS
(China)		TACE	105	94%	51	NA	87	NA	25.7	NA	NA	OS
Hu 2024 (26)	Retro	TACE+LR	98	85%	NA	NA	91	NA	39.8	NA	127	OS
(China)		TACE	98	90%	NA	NA	84	NA	39.8	NA	70	OS
Zhu 2023 (21)	Retro	TACE+ICIs+ TKIs	78	NA	NA	NA	NA	NA	NA	NA	NA	OS/PFS
(China)		TACE+TKIs	77	NA	NA	NA	NA	NA	NA	NA	NA	OS/PFS
Huang 2023 (57)	Retro	LR	61	NA	NA	NA	NA	NA	NA	NA	NA	OS
(China Taipei)		TACE	168	NA	NA	NA	NA	NA	NA	NA	NA	OS
Li 2023 (52)	Retro	TACE+ICIs+ TKIs	39	NA	NA	NA	NA	NA	NA	NA	NA	OS/PFS
(China)		TACE+TKIs	28	NA	NA	NA	NA	NA	NA	NA	NA	OS/PFS
Chen 2019 (62)	Retro	LR	623	95%	51	99/1	96	3	47	NA	32	OS
(China)		TACE	623	94%	51	99/1	96	2	47	NA	32	OS
Yan 2020 (27)	Retro	LR+RFA	42	81%	49	74/26	100	NA	2.7 (2-5)	6.0 (mean)	36	OS
(China)		TACE	84	90%	52	65/35	100	NA	2.5 (2-5)	6.7 (mean)	14	OS
Kudo 2018 (17)	RCT	TACE+TKIs (Orantinib)	209	NA	NA	NA	NA	NA	NA	NA	NA	OS
(Japan)		TACE	229	NA	NA	NA	NA	NA	NA	NA	NA	OS
Zhou 2019 (40)	Retro	LR+RFA	47	89%	NA	NA	NA	NA	60.6	NA	NA	OS
(China)		TACE	94	89%	NA	NA	NA	NA	59.6	NA	16	OS
Kudo 2020 (72)	RCT	TACE+TKIs (Sorafenib)	44	NA	NA	NA	NA	NA	NA	NA	NA	PFS
(Japan)		TACE	34	NA	NA	NA	NA	NA	NA	NA	NA	PFS
Lencioni 2016 (15)	RCT	TACE+TKIs (Sorafenib)	154	88%	65	88/12	36	25	NA	NA	26	OS/TTP
(USA)		TACE	153	82%	63	82/18	33	27	NA	NA	26	OS/TTP
Kim 2016 (53)	Retro	LR	52	9%	55	98/2	NA	NA	19.2	NA	30	OS
(Korea)		TACE	225	86%	58	83/17	NA	NA	60	NA	30	OS
Endo 2018 (61)	Retro	TACE+RFA	46	76%	74	78/22	NA	NA	NA	3.2	22	OS
(Japan)		TACE	46	65%	74	67/33	NA	NA	NA	3.4	30	OS
Lin 2020 (51)	Retro	TACE+RFA	56	75%	64	77/23	56	37	NA	5.5	26	OS
(China)		TACE	231	75%	64	84/16	45	39	NA	7	21	OS
Lin 2020 (51)	Retro	LR	140	84%	62	96/4	50	21	35	8.2	39	OS
(China)		TACE	87	77%	64	92/8	41	30	47	8.2	21	OS
Hirooka 2018 (59)	Retro	TACE+RFA	32	78%	70 (mean)	91/9	13	66	NA	4.5	82	OS
(Japan)		TACE	32	88%	71 (mean)	97/3	0	75	NA	4.3	28	OS
Nouso 2017 (49)	Retro	TACE+RFA	31	NA	NA	NA	NA	NA	NA	NA	60	OS
(Japan)		TACE	31	NA	NA	NA	NA	NA	NA	NA	44	OS
Peng 2020 (47)	Retro	LR	70	86%	54	96/4	76	NA	24	5	40	OS
(China)		TACE	70	87%	57	97/3	79	NA	26	5	20	OS
Ke 2014 (55)	Retro	LR	53	92%	47 (mean)	NA	NA	NA	NA	8.5	35	OS
(China)		TACE	53	92%	47 (mean)	NA	NA	NA	NA	8.9	20	OS
Chen 2020 (63)	Retro	TACE+idoine 125	38	NA	NA	NA	NA	NA	NA	NA	38	OS
(China)		TACE+RFA	74	NA	NA	NA	NA	NA	NA	NA	33	OS
Tada 2017 (44)	Retro	LR	132	17%	69	NA	19	58	NA	NA	65	OS
(Japan)		TACE	132	17%	69	NA	12	62	NA	NA	42	OS
Espinosa 2018 (60)	Retro	LR+ RFA	26	85%	63 (mean)	NA	42	50	35	5.4	60	OS/TTP
(Philippines)		TACE	78	87%	64 (mean)	NA	38	54	27	5.4	40	OS/TTP
Hou 2016 (58)	Retro	LR+RFA	51	85%	51 (mean)	NA	27	10	NA	10.5	38	OS
(China)		TACE	102	91%	53 (mean)	NA	34	8	NA	10.7	17	OS
Kariyama 2020 (56)	Retro	RFA	89	NA	NA	NA	NA	NA	NA	NA	50	OS
(Japan)		TACE	89	NA	NA	NA	NA	NA	NA	NA	38	OS
Kim 2022 (54)	Retro	LR+RFA	95	85%	NA	NA	74	8	NA	NA	133	OS/PFS
(Korea)		TACE	252	90%	NA	NA	76	8	NA	NA	48	OS/PFS
Ohama 2022 (48)	Retro	LR	45	73%	67	100/0	13	53	NA	5.3	41	OS
(Japan)		LR+RFA	25	84%	68	88/12	16	52	NA	4.8	41	OS
Zhou 2020 (39)	Retro	TACE+LR	189	89%	NA	NA	NA	NA	27	NA	NA	OS
(China)		LR	189	90%	NA	NA	NA	NA	23	NA	NA	OS
Kudo 2018	RCT	TACE+TKIs (Orantinib)	209	NA	NA	NA	NA	NA	NA	NA	NA	OS
(Japan)		TACE	229	NA	NA	NA	NA	NA	NA	NA	NA	OS
Wang 2021 (43)	Retro	LR+TACE	123	88%	NA	NA	92	NA	NA	NA	39	OS
(China)		LR	123	90%	NA	NA	91	NA	NA	NA	30	OS
Yang 2022 (41)	Retro	LR	31	84%	52 (mean)	NA	42	0	NA	NA	38	OS
(China)		TACE	31	81%	52 (mean)	NA	23	10	NA	NA	53	OS
Nong 2023 (50)	Retro	LR	108	89%	NA	NA	92	NA	3.7	NA	95	OS/PFS
(China)		TACE	55	87%	NA	NA	89	NA	16.4	NA	31	OS/PFS
Lencioni 2024 (22)	RCT	TACE+ICIs+ bevacizumab	117	NA	NA	NA	NA	NA	NA	NA	NA	PFS
(USA-ASCO)		TACE	114	NA	NA	NA	NA	NA	NA	NA	NA	PFS
Lu 2021 (11) (China)	Retro	LR	169	91%	51 (mean)	80/20	99%	NA	42	6.4 (mean)	67	OS
		TACE	169	95%	54 (mean)	83/17	98%	NA	40.8	6.4 (mean)	30	OS
Luo 2011 (67)	Non-RCT	LR	85	82%	47.5 (mean)	71/5	82%	2.3	58.8	8.7 (mean)	22.5	OS
(China)		TACE	83	95%	50.9 (mean)	86/1	92%	4.8	61.4	7.8 (mean)	19.5	OS
Xu 2018 (68)	Retro	LR	259	83%	56.8 (mean)	100/0	68%	7	1.9	NA	NA	OS
(China)		TACE	372	85%	55.6 (mean)	99/1	72%	9	10.2	NA	NA	OS
Ciria 2015 (69)	Retro	LR	36	78%	67	78/22	14%	27.8	NA	8.6	NA	OS
(Spain)		TACE	44	75%	65	66/34	16%	43.2	NA	5.4	NA	OS
Yin 2014 (24)	RCT	LR	88	93%	51.6	98.9	71.6	3.4	37.5	7.3	41	OS
(China)		TACE	85	93%	54	94.1	90.6	1.2	43.5	7.4	14	OS
Zhang 2018 (71)	Retro	TACE+MWA	50	86%	NA	92/8	NA	NA	54	NA	17.5	OS/PFS
(China)		TACE	100	91%	NA	94/6	NA	NA	54	NA	16.1	OS/PFS
Ho 2009 (70)	Retro	BSC	70	NA	NA	NA	NA	NA	NA	NA	NA	OS
(China Taiwan)		TACE	163	NA	NA	NA	NA	NA	NA	NA	NA	OS
Ho 2009 (70)	Retro	LR	122	NA	NA	NA	NA	NA	NA	NA	NA	OS
(China Taiwan)		TACE	163	NA	NA	NA	NA	NA	NA	NA	NA	OS

RCT, Randomized control trails; Retro, Retrospectives; CTP, Child-Turcotte-Pugh; HBV, Hepatitis B virus; HCV, Hepatitis C virus; NA, Not associated; LR, Liver resection; TACE, Transarterial chemoembolization; PD-(L)1, ICIs (immune checkpoint inhibitors); TKIs, tyrosine kinase inhibitors; OS, Overall survival; PFS, Progression free survival.

### Risk of bias assessment

ROBINS-I is composed of confounding, selection of participants, classification of interventions, deviation from intended interventions, missing data, measurement of the outcome, and selection of the reported results. Five studies were considered to be at high risk, 12 studies were identified as moderate risk, and 21 studies were considered to be at low risk. Details of the assessments are shown in **Supplementary Tables S4**, **S5**.

### Primary outcome: overall survival

There were a total of 36 studies (one prospective non-randomized analysis, three RCTs, and 32 retrospective studies) and 12 treatment therapies. The pairwise meta-analysis showed that several regimens had better OS benefit than TACE alone, including TACE plus radiofrequency ablation (RFA) [adjusted HR (aHR) = 0.57, 95%CI = 0.42–0.78], liver resection (aHR = 0.53, 95%CI = 0.44–0.62), liver resection plus RFA (aHR = 0.52, 95%CI = 0.41–0.66), and TACE plus tyrosine kinase inhibitors (TKIs) (aHR = 0.86, 95%CI = 0.68–1.09). The results are presented in **Table 2**.

**Figure 2:**
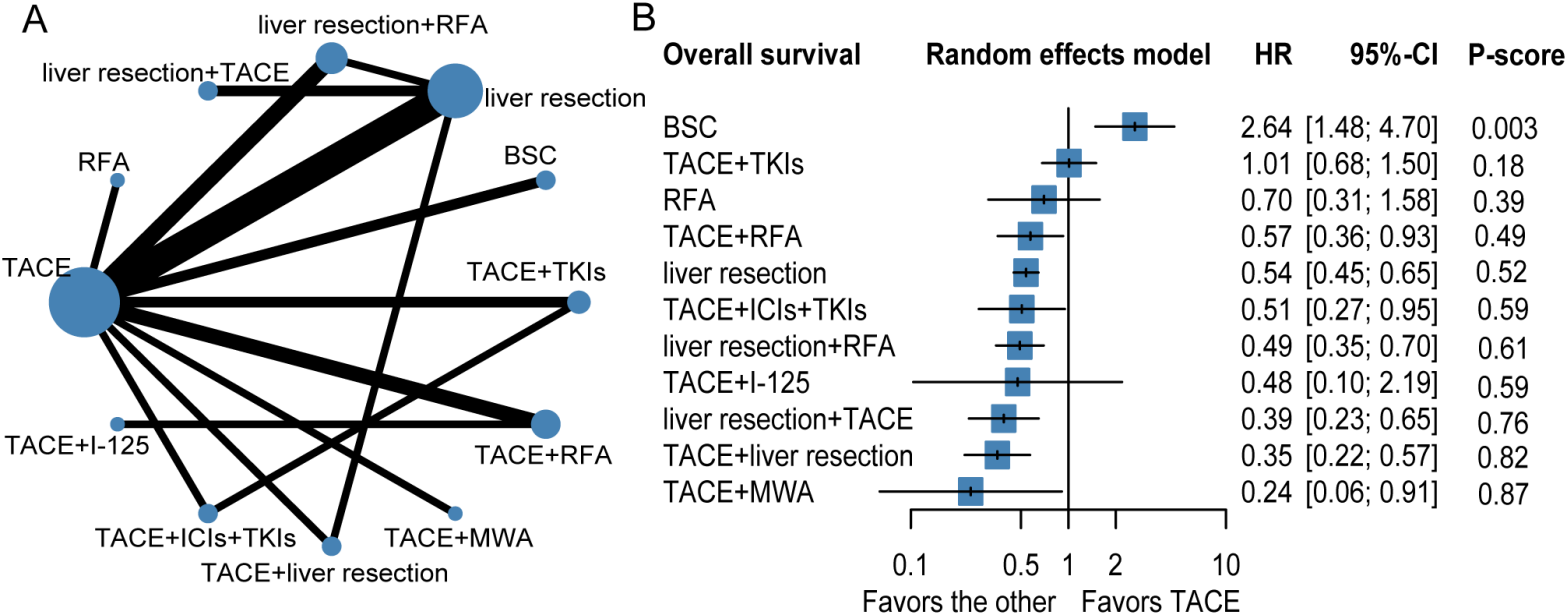
Network plot **(A)**, forest plot **(B)** for overall survival. The therapeutic regimens with direct comparisons are linked by lines, the width of lines is proportional to the number of trials comparing each pair of interventions. The size of each node is proportional to the number of sample size. BSC, best supportive care; TKIs, tyrosine kinase inhibitors; RFA, radiofrequency ablation; TACE, transarterial chemoembolization; ICIs, immune checkpoint inhibitors; I-125, iodine 125 seeds.

The results of the NMA are shown in **Figure 2**, **Table 2**. Several therapeutic regimens had significantly better OS than TACE alone, including TACE plus microwave ablation (MWA) (aHR = 0.24, 95%CI = 0.06–0.91, *P*-score = 0.87; low confidence), TACE plus liver resection (aHR = 0.35, 95%CI = 0.22–0.57, *P*-score = 0.82; high confidence), liver resection plus TACE (aHR = 0.39, 95%CI = 0.23–0.65, *P*-score = 0.76; low confidence), liver resection plus RFA (aHR = 0.49, 95%CI = 0.35–0.70, *P*-score = 0.61; high confidence), TACE combined with ICIs with TKIs (aHR = 0.51, 95%CI = 0.27–0.95, *P*-score = 0.59; very low confidence), liver resection (aHR = 0.54, 95%CI = 0.45–0.65, *P*-score = 0.52; low confidence), and TACE plus RFA (aHR = 0.57, 95%CI = 0.36–0.93, *P*-score = 0.49; very low confidence). There were no significant differences in OS improvement between TACE and the following therapies: TACE plus I-125, TACE plus TKIs, and best supportive care (BSC). Notably, TACE plus MWA ranked the highest out of all 12 treatment therapies for prolonging OS. TACE plus liver resection and liver resection plus TACE were the second and third best therapies, respectively. The evidence credibility of the NMA results is shown in **Supplementary Table S7**, **Supplementary Figure S1**.

**Table 2:** Comparative efficacy of different therapies on overall survival (OS).

**BSC**	NA	NA	NA	NA	2.64 (1.48-4.70)	NA	NA	NA	NA	NA	NA
4.90 (2.68- 8.97)	**LR**	1.09 (0.35-3.37)	1.38 (0.86-2.23)	NA	0.53 (0.44-0.63)	NA	NA	1.94 (1.08- 3.49)	NA	NA	NA
5.37 (2.74-10.52)	1.09 (0.75-1.61)	**LR+RFA**	NA	NA	0.49 (0.34-0.71)	NA	NA	NA	NA	NA	NA
6.79 (3.15-14.64)	1.38 (0.86-2.23)	1.26 (0.69-2.33)	**LR+TACE**	NA	NA	NA	NA	NA	NA	NA	NA
3.77 (1.39-10.22)	0.77 (0.33-1.77)	0.70 (0.29-1.70)	0.56 (0.21-1.45)	**RFA**	0.70 (0.31-1.58)	NA	NA	NA	NA	NA	NA
2.64 (1.48- 4.70)	0.54 (0.45-0.65)	0.49 (0.35-0.70)	0.39 (0.23-0.65)	0.70 (0.31-1.58)	**TACE**	NA	1.19 (0.56-2.52)	1.89 (0.88-4.02)	4.17 (1.10-15.76)	1.74 (1.08-2.81)	1.15 (0.76-1.74)
5.54 (1.09-28.29)	1.13 (0.24-5.25)	1.03 (0.22-4.94)	0.82 (0.16-4.08)	1.47 (0.26-8.28)	2.10 (0.46-9.65)	**TACE+I-125**	NA	NA	NA	0.83 (0.20-3.53)	NA
5.21 (2.22-12.23)	1.06 (0.55-2.05)	0.97 (0.47-1.99)	0.77 (0.34-1.73)	1.38 (0.49-3.87)	1.98 (1.05-3.70)	0.94 (0.18-4.90)	**TACE+ICIs** **+TKIs**	NA	NA	NA	0.18 (0.06-0.52)
7.46 (3.53-15.77)	1.52 (0.95-2.43)	1.39 (0.77-2.50)	1.10 (0.56-2.14)	1.98 (0.77-5.08)	2.83 (1.75-4.56)	1.35 (0.27-6.65)	1.43 (0.65-3.15)	**TACE+LR**	NA	NA	NA
11.00 (2.58-46.88)	2.24 (0.59-8.59)	2.05 (0.52-8.11)	1.62 (0.39-6.73)	2.92 (0.61-13.87)	4.17 (1.10-15.76)	1.98 (0.26-15.01)	2.11 (0.48-9.18)	1.47 (0.36-6.06)	**TACE+MWA**	NA	NA
4.60 (2.18- 9.72)	0.94 (0.56-1.56)	0.86 (0.47-1.55)	0.68 (0.34-1.36)	1.22 (0.47-3.13)	1.74 (1.08-2.81)	0.83 (0.20-3.53)	0.88 (0.40-1.94)	0.62 (0.31-1.21)	0.42 (0.10-1.72)	**TACE+RFA**	NA
2.61 (1.30- 5.25)	0.53 (0.35-0.82)	0.49 (0.29-0.82)	0.39 (0.20-0.73)	0.69 (0.28-1.71)	0.99 (0.67-1.47)	0.47 (0.10-2.28)	0.50 (0.26-0.98)	0.35 (0.19-0.65)	0.24 (0.06-0.95)	0.57 (0.31-1.06)	**TACE+TKIs**

### Secondary outcome: progression-free survival

**Figure 3:**
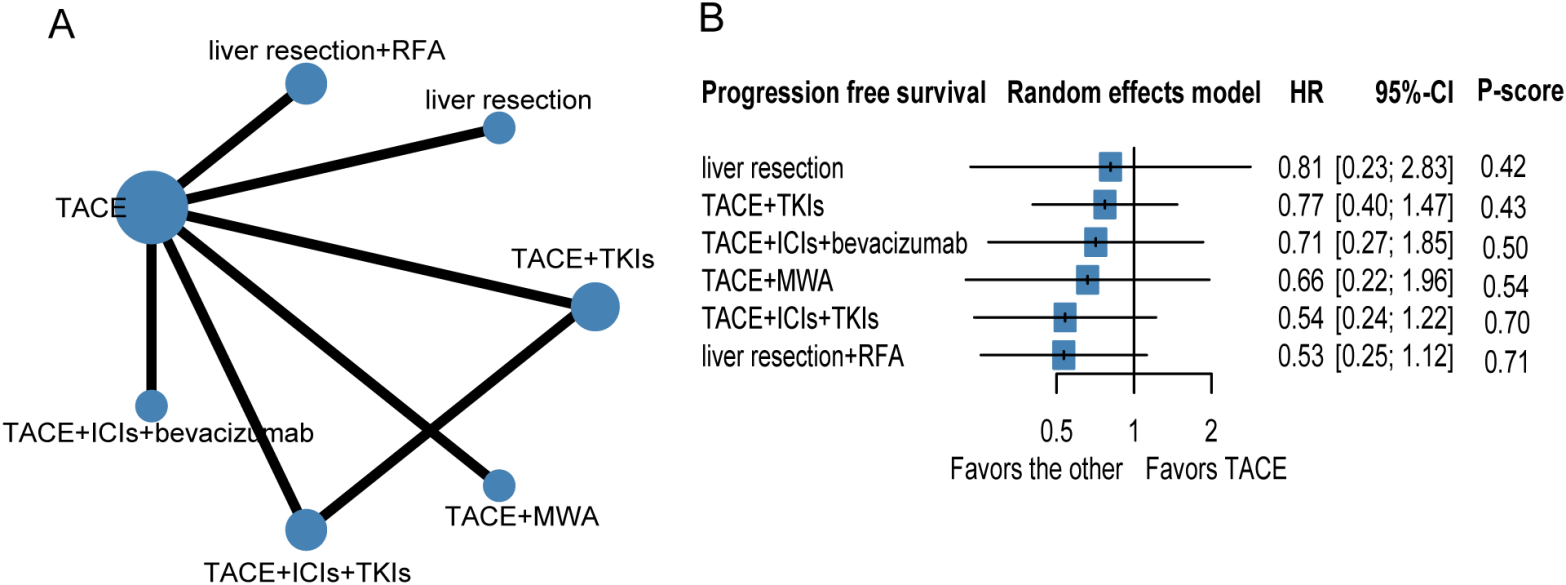
Network plot **(A)** and forest plot **(B)** for progression free survival. Circle sizes reflect numbers of participants, while line widths reflect numbers of direct comparisons. The absence of a connecting line between two treatments indicates that there was no direct comparison. TKIs, tyrosine kinase inhibitors; RFA, radiofrequency ablation; TACE, transarterial chemoembolization; ICIs, immune checkpoint inhibitors.

To evaluate progression-free survival (PFS), nine studies and seven therapeutic regimens in 1,776 patients with HCC were selected for further analysis (**Figure 3**, **Table 3**). No therapies showed better PFS benefit than TACE alone. Liver resection plus TACE (*P*-score = 0.71) and TACE combined with ICIs and TKIs (*P*-score = 0.70) were ranked first and second, respectively. In addition, pairwise treatment comparisons also indicated that no therapies improved PFS compared with TACE alone (**Supplementary Table S8**). The evidence credibility of the NMA results is shown in **Supplementary Table S9**, **Supplementary Figure S2**.

**Table 3:** Comparative efficacy of different therapies on progression free survival (PFS).

**LR**	NA	0.81 (0.23-2.83)	NA	NA	NA	NA
1.52 (0.35-6.50)	**LR+RFA**	0.53 (0.25-1.12)	NA	NA	NA	NA
0.81 (0.23-2.83)	0.53 (0.25-1.12)	**TACE**	1.41 (0.54-3.68)	1.18 (0.42-3.27)	1.52 (0.51-4.50)	1.62 (0.79-3.30)
1.14 (0.24-5.52)	0.75 (0.22-2.53)	1.41 (0.54-3.68)	**TACE+ICIs+bevacizumab**	NA	NA	NA
1.50 (0.34-6.67)	0.99 (0.33-2.97)	1.85 (0.82-4.18)	1.32 (0.37-4.63)	**TACE+ICIs+TKIs**	NA	0.40 (0.13-1.24)
1.23 (0.23-6.45)	0.81 (0.22-3.02)	1.52 (0.51-4.50)	1.08 (0.25-4.59)	0.82 (0.21-3.18)	**TACE+MWA**	NA
1.05 (0.26-4.30)	0.69 (0.26-1.85)	1.30 (0.68-2.48)	0.92 (0.29-2.93)	0.70 (0.30-1.62)	0.86 (0.24-3.04)	**TACE+TKIs**

### Subgroup analysis

Intermediate-stage (BCLC-B) HCC is characterized by substantial heterogeneity. Therefore, subgroup analysis was performed to identify the optimal patient group for these effective regimens. In terms of the primary endpoint (i.e., OS), subgroup analysis was performed according to a series of clinical parameters, including region, Child–Pugh class, AFP level, etiology (HBV or HCV infection), tumor number, and tumor size. As shown in **Table 1**, there was significant heterogeneity in terms of etiology among the included studies. Subgroup analysis was performed according to the median value of these clinical parameters (**Table 4**).

**Table 4:** Subgroup analysis of OS.

Characteristics [median, range]	Samples	No. studies	Therapy (rank first)	HR 95% CI	P-score
Region					
Asia (Excluding Japan)	7178	29	TACE+MWA	0.24 (0.06-0.92)	0.87
Rest of the world	1555	9	Liver resection	0.55 (0.33-0.96)	0.72
Child-Pugh class A (%) [94 (78-99)]					
Total	4229	14	Liver resection+RFA	0.47 (0.31-0.71)	0.85
<94	1153	7	Liver resection+RFA	0.51 (0.26-1.01)	0.80
≥94	3076	7	Liver resection+RFA	0.43 (0.24-0.78)	0.90
AFP level (≥400 ng/ml, %) [37 (30-45)]					
Total	3150	10	Liver resection+RFA	0.22 (0.08-0.60)	0.85
<37	927	5	Liver resection+RFA	0.22 (0.06-0.75)	0.95
≥37	2223	5	TACE+liver resection	0.25 (0.10-0.59)	0.88
HBV infected (%) [73 (35-90)]					
Total	6007	24	Liver resection+TACE	0.37 (0.21-0.64)	0.92
<73	2397	12	Liver resection	0.51 (0.37-0.70)	0.83
≥73	3610	12	Liver resection+TACE	0.37 (0.20-0.68)	0.89
HCV infected (%) [26 (7-52)]					
Total	3741	14	TACE+RFA	0.54 (0.34-0.86)	0.84
<26	2780	7	Liver resection+RFA	0.66 (0.44-0.98)	0.75
≥26	961	7	Liver resection+RFA	0.40 (0.18-0.92)	0.83
Tumor number (≥3, %) [35 (23-NA)]					
Total	2988	12	TACE+MWA	0.24 (0.06-0.98)	0.76
<35	1736	6	TACE+liver resection	0.23 (0.08-0.65)	0.80
≥35	1252	6	TACE+MWA	0.24 (0.06-1.01)	0.88
Tumor size (cm) [6 (5-8)]					
Total	1635	11	Liver resection	0.46 (0.33-0.62)	0.73
<5	470	5	Liver resection+RFA	0.29 (0.13-0.68)	0.79
≥5	1165	6	Liver resection	0.48 (0.33-0.69)	0.88
Sample size [88 (47-134)]					
<88	1227	13	Liver resection	0.44 (0.22-0.91)	0.72
≥88	5218	13	TACE+liver resection	0.38 (0.28-0.52)	0.95

HR, hazard ratio; Age and tumor size, mean values or median values of age and main tumor size; Gender/male percentage, the proportion of male patients.

Subgroup analysis was also performed according to the region of origin. TACE plus MWA (aHR = 0.24, 95%CI = 0.06–0.92) was associated with the highest OS benefit in Asia (excluding Japan) (**Supplementary Table S10**, **Supplementary Figure S3**), while liver resection ranked first in the rest of the region (aHR = 0.55, 95%CI = 0.33–0.96). The results are shown in **Supplementary Table S11**, **Supplementary Figure S4**. In terms of AFP level (**Supplementary Figure S8**), eight studies with 2,836 patients were included. Liver resection plus RFA ranked highest in patients with lower AFP levels (aHR = 0.22, 95%CI = 0.06–0.75) (**Supplementary Figure S9**, **Supplementary Table S14**). For the subgroup with higher AFP levels (**Supplementary Figure S10**, **Supplementary Table S15**), the combination of TACE and liver resection was associated with the greatest reduction in risk of death (aHR = 0.25, 95%CI = 0.10–0.59).

For the subgroup analysis of HBV infection (**Supplementary Figure S11**), 24 studies with 6,007 patients were included. Liver resection ranked highest in patients with lower HBV infection (aHR = 0.51, 95%CI = 0.37–0.70) (**Supplementary Figure S12**, **Supplementary Table S16**), while liver resection plus TACE ranked first in terms of improving OS in patients with higher HBV infection (aHR = 0.37, 95%CI = 0.20–0.68) (**Supplementary Figure S13**, **Supplementary Table S17**). In addition, a total of 14 studies with 3,741 patients showed the HCV infection status (**Supplementary Figure S14**). Subgroup analysis showed that liver resection plus RFA was reported as the highest therapy with the highest improvement in OS in patients with HCV infection (**Supplementary Table S18**, **Supplementary Figure S16**). In addition, 11 studies with 1,635 patients reported data on tumor size (**Supplementary Figure S19**), with the median value of tumor size being 6 cm. Liver resection plus RFA ranked highest in patients with smaller tumor size (aHR = 0.29, 95%CI = 0.13–0.68) (**Supplementary Figure S20**, **Supplementary Table S21**). For the subgroup with bigger tumor size, liver resection was significantly associated with the greatest reduction in risk of death (aHR = 0.48, 95%CI = 0.33–0.69) (**Supplementary Figure S21**, **Supplementary Table S22**). Notably, in the subgroup of bigger sample size, a total of 13 studies with 5,218 patients were included. TACE plus liver resection had the highest OS benefit in this subgroup (HR = 0.38, 95%CI = 0.28–0.52). Details of the subgroup analysis are shown in **Supplementary Table S24**, **Supplementary Figure S24**.

### Assessment of inconsistency

Overall, the results of the design-by-treatment interaction test indicated a slight inconsistency in terms of OS (*p* = 0.039). There was no significant difference for PFS in terms of inconsistency between direct and indirect estimates in the back-calculation and design-by-treatment interaction test (*p* = 0.099).

## Discussion

To date, BCLC classification is the most widely used treatment algorithm for HCC around the world. TACE is still the recommended standard therapy for BCLC-B stage HCC. However, this stage is characterized by substantial heterogeneity. In addition, patients treated with several therapy combinations have shown longer survival times compared with those with TACE alone. To compare the efficacy of these therapies, NMA was performed, which included 10,972 patients who received 13 different therapies across 38 clinical studies.

The results of the NMA revealed that TACE plus thermal ablation, TACE plus resection, TACE plus ICIs plus TKIs, liver resection, liver resection plus TACE, and liver resection plus RFA significantly improved OS compared with TACE alone. On the other hand, no monotherapies or combination treatments significantly prolonged the PFS compared with TACE alone. Notably, TACE plus MWA showed the best efficacy in improving OS. The superiority of TACE plus MWA can be explained as follows: 1) chemoembolization could significantly reduce the tumor size, then increase the complete ablation rate; 2) the undetected micronodules and the margin of active tumors can be identified by hepatic artery angiography, which then guides further ablation; and 3) the blood supply artery of the tumor can be embolized using TACE, which then reduces the cooling effect of blood flow and enhances the efficacy of ablation. In addition, the efficacy of TACE plus MWA is superior to TACE plus RFA in terms of OS, which can be attributed to MWA able to result in higher intratumor temperature, larger necrosis area, and deeper tissue penetration than RFA. Consistently, TACE plus RFA is suitable for patients in the BCLC-B stage up to seven (51, 73). Moreover, patients with two or three tumors, and intermediate tumor size (3–5 cm), could benefit from TACE plus MWA (74–76). Subgroup analysis showed that TACE plus RFA significantly prolonged the OS in patients with poor liver function, no history of HBV infection, HCV infection, and in old patients (**Table 5**).

**Table 5:** The optimal therapy for BCLC-B stage HCC based on clinical characteristics.

Therapy	Number	Size (cm)	Location	AFP	HBV infected	HCV infected	Age (year)	Child-Pugh class
TACE+MWA	2-3	3-5	NA	NA	Negative	Positive	>58	A/B
TACE+RFA	Up-to-seven		NA	NA	Negative	Positive	>58	A/B
TACE+resection	2	3-5	NA	NA	NA	NA	NA	NA
Liver resection	2-3	5-8	NA	NA	Negative	NA	NA	A
Resection+TACE	2-3	NA	NA	>400	Positive	NA	<58	NA
Resection+RFA	<5	Dominant: <6cm Ablated: <2cm	unfavorable	<400	Negative	Positive	>58	A
TACE+ICIs+TKIs	unresectable		NA	NA	NA	NA	NA	NA

NA, Not association; Dominant, Dominant size; Ablated, Ablated size.

Our study showed that liver resection provides significant OS improvement compared with TACE alone, which is consistent with a previous meta-analysis (77). Notably, we excluded patients with solitary large HCC (>5 cm), classified as BCLC-A stage in the updated BCLC classification. Huang et al. confirmed that liver resection showed better OS than TACE in patients with middle-high tumor burden (up to 7), but with tumor numbers ≤3 and preserved liver function (57). Consistently, subgroup analysis also found that liver resection ranked first among patients with intermediate tumor size (6 cm, range = 5–8 cm), a lower proportion of HBV-infected patients, and non-Asian areas (excluding Japan). Most of the patients with HCC had a history of HBV infection in Asia, but non-viral or HCV infection in Japan and in western countries (78). Moreover, the HBV-associated HCC patients with positive HBcAb showed a higher risk of recurrence after liver resection (79); hence, postoperative therapies are needed for these patients. Notably, subgroup analysis found that adjuvant TACE showed the best efficacy in prolonging OS in patients with positive HBcAb. Therefore, liver resection is suitable for intermediate-stage patients with two or three nodules, median tumor size (5–8 cm), preserved liver function, and negative HBcAb (**Table 5**).

Preoperative TACE ranked second in terms of prolonging OS in the NMA. TACE was performed as a neoadjuvant and transformative therapy in the clinic, the aim of which is to reduce the tumor size, downstage the cancer, and eventually enhance the efficacy of surgery, as well as even creating opportunities for resection. However, the clinical value of preoperative TACE in large (≥5 cm) resectable HCC is still uncertain. A prospective study confirmed that preoperative TACE did not improve OS in patients with resectable large HCC (≥5 cm, BCLC-A/B stage). This study was not included in our NMA due to the lack of subgroup analysis. Notably, the included studies also found that intemediate-stage patients with up to 7 (two tumors, tumor size ≤5 cm) could benefit from preoperative TACE (26, 39). Consistently, subgroup analysis found that preoperative TACE ranked first in terms of prolonging OS in patients in the BCLC-B stage with two nodules (**Table 5**).

Notably, our NMA found that adjuvant TACE significantly prolonged OS compared with TACE alone. Its efficacy ranked third among all therapies. The 5-year postoperative recurrence rate of HCC is up to 75% (80). Theoretically, TACE is performed to identify and eliminate latent micro-metastasis after resection, then to prevent HCC recurrence. However, the efficacy of adjuvant TACE for HCC is controversial. Some clinical studies have suggested that patients with intermediate (solitary tumor ≥5 cm without microvascular invasion) or high risk (a single tumor with microvascular invasion, two or three tumor nodules) of recurrence could benefit from adjuvant TACE (43, 81), particularly for HBV-related HCC (82). Consistently, subgroup analysis also showed that patients with two or three tumors, elevated AFP (≥400 ng/ml), and HBV infection and those aged <58 years could benefit from adjuvant TACE (**Table 5**).

As described above, curative resection provided better efficacy than TACE for a particular subgroup of BCLC-B stage patients. However, considerable intermediate-stage HCC patients showed no chance of resection due to the unfavorable tumor location, insufficient future liver remnant (FLR), and excessive tumors (four or more). To strive for curative chances for patients who can benefit from hepatectomy, partial hepatectomy plus RFA was introduced (58). This combination therapy is performed simultaneously to eradicate all tumor nodules through hepatectomy of the main tumor and RFA of the residual lesions. The included studies found that this combination therapy may be suitable for a particular subgroup of BCLC-B stage HCC patients, including those with preserved liver function, resectable dominant lesions restricted to one lobe, ≤4 or ≤5 tumors (with the main tumor size being <6 cm), and limited ablated tumor size (≤2 cm) (27, 58). Consistently, subgroup analysis also confirmed that patients with preserved liver function (Child–Pugh class A), dominant tumor size <6 cm, low AFP (<400 ng/ml), no history of HBV and HCV infection, are men, and aged >58 years could benefit from this combination therapy (**Table 5**).

With the advent of the immune targeting era, several RCTs and real-world studies have been conducted to evaluate the efficacy of TACE combined with ICIs and MTTs for the treatment of HCC. Our NMA found that this triple therapy could significantly prolong OS. However, the included participants of an RCT (EMERALD-1) and a real-world study (CHANCE001) were unresectable or were BCLC-B/C stage HCC patients. Consistently, the latest phase 3 LEAP-012 study (83) confirmed that TACE plus lenvatinib plus pembrolizumab significantly prolonged the PFS in unresected, non-metastatic HCC compared with TACE. Subgroup analysis also obtained positive results in unresectable BCLC-B stage HCC patients (HR = 0.57, 95%CI = 0.41–0.77), although not significant in terms of OS. Longer follow-up is needed. Thus, participants with unresectable BCLC-B stage HCC comprised one part of the included patients. TACE plus TKIs plus ICIs may be suitable for partial unresectable BCLC-B stage HCC. We could not further perform detailed subgroup analysis based on tumor size, tumor number, etc. The optimal BCLC-B stage patients who could benefit from this triple therapy need further validation (**Table 5**).

Intermediate-stage (BCLC-B) HCC is characterized by substantial heterogeneity. TACE is the standard therapeutic approach for patients in this stage. However, TACE is possibly overused and may not be appropriate for all patients. Our results revealed that seven therapies showed OS benefit over TACE alone: liver resection, liver resection plus TACE, liver resection plus RFA, TACE plus ICIs plus TKIs, TACE plus RFA, TACE plus MWA, and TACE plus liver resection showed better OS benefit than TACE alone. Subgroup analysis indicated that these therapies are suitable for particular intermediate-stage HCC patients. These results could be used to guide further clinical studies and facilitate clinical decision-making. However, only a portion of the included studies reported detailed clinical parameters. Due to the limited number of studies and the small sample size of the subgroups, the efficacy of TACE plus another therapy among all subtypes could not be accurately evaluated. Further high-quality clinical studies are needed to further verify these findings.

This NMA has several limitations. Firstly, all NMA studies are limited by assumptions of transitivity and similarity. The methodologies in the included studies were comparable, and the characteristics of the patients were similar. Several retrospective studies were included in this NMA. To reduce the RoB of retrospective studies, only the results of the propensity score matching (PSM) analysis were included. In addition, design-adjusted analysis was performed according to the RoB (studies with a higher bias risk were assigned a lower weight). Secondly, the HR and 95%CI were not reported in some studies. We extracted these data from the Kaplan–Meier curves. Thirdly, there was a lack of detailed clinical characteristics in some studies; therefore, the subgroup analysis could not be performed accordingly. Finally, there exists heterogeneity among the included studies. The median follow-up time of the included trials differed. Moreover, the procedures for TACE and thermal ablation varied across the included studies.

## Conclusion

In summary, it was found that a series of therapeutic regimens, including TACE plus MWA, preoperative TACE, TACE plus TKIs plus ICIs, TACE plus RFA, liver resection, and adjuvant therapies (TACE and RFA) showed better OS benefit than TACE alone. This might have implications in informed decision-making when considering the optimal treatment regimen for patients with intermediate-stage HCC. Despite confidence in the findings being high for preoperative TACE and liver resection plus RFA, low for TACE plus MWA and adjuvant RFA and very low for all others. Therefore, more therapeutic regimens and high-quality studies are needed.

## Data Availability

The original contributions presented in the study are included in the article/**Supplementary Material**. Further inquiries can be directed to the corresponding authors.
