# Supplementary material for: Efficacy of therapies for intermediate-stage hepatocellular carcinoma: systematic review and network meta-analysis
Source: Front Immunol. 2025 Jul 9;16:1577614. doi: 10.3389/fimmu.2025.1577614 (PMC12283577; doi:10.3389/fimmu.2025.1577614)
Supplement: Supplementary file 1 [file DataSheet1.docx]

Appendix

[Table s1 PRISMA NMA Checklist of Items to Include When Reporting A Systematic Review Involving a Network Meta-analysis 8](#_Toc200204521)

[Table s2 PRISMA checklist for NMA abstract 12](#_Toc200204522)

[Table s3 Seach Strategy 14](#_Toc200204523)

[**PubMed** 14](#_Toc200204524)

[**Embase** 14](#_Toc200204525)

[**Cochrane** 15](#_Toc200204526)

[Table s4 Risk of bias in studies reporting on risk of OS 18](#_Toc200204527)

[Table s5 Risk of bias in studies reporting on risk of PFS 40](#_Toc200204528)

[Table s6 Standard meta-analysis of OS 46](#_Toc200204529)

[Figure s1 Risk-of-bias contribution chart (percent stacked bar chart) to assess the credibility of evidence using CINeMA tool in terms of OS 47](#_Toc200204530)

[Table s7 Assessing the credibility of evidence using the CINeMA tool in terms of OS 48](#_Toc200204531)

[Figure s3 Risk of overall survival in studies in Asia (excluding Japan, n= 29 studies) 53](#_Toc200204532)

[Table s10 Risk of overall survival in studies in Asia (excluding Japan, n= 29 studies) 53](#_Toc200204533)

[Figure s4 Risk of overall survival in studies in rest of region (n= 9 studies) 55](#_Toc200204534)

[Table s11 Risk of overall survival in studies in rest of region (n= 9 studies) 55](#_Toc200204535)

[Figure S5 Distribution of Child-Pugh class 56](#_Toc200204536)

[Figure s6 Risk of overall survival in studies with lower percentage of CT-A class (n= 14 studies) 57](#_Toc200204537)

[Table s12 Risk of overall survival in studies with lower percentage of CT-A class (n=14 studies) 57](#_Toc200204538)

[Figure s7 Risk of overall survival in studies with a higher percentage of CT-A class (n=7 studies) 58](#_Toc200204539)

[Table s13 Risk of overall survival in studies with a higher percentage of CT-A class (n=7 studies) 58](#_Toc200204540)

[Figure s8 Distribution of AFP level across OS 59](#_Toc200204541)

[Figure s9 Risk of overall survival in studies with a lower level of AFP (n=5 studies) 60](#_Toc200204542)

[Table s14 Risk of overall survival in studies with a lower level of AFP (n=5 studies) 60](#_Toc200204543)

[Figure s10 Risk of overall survival in studies with a higher level of AFP (n=5 studies) 61](#_Toc200204544)

[Table s15 Risk of overall survival in studies with a higher level of AFP (n=5 studies) 61](#_Toc200204545)

[Figure s11: Distribution of the percentage of HBV-infected patients across OS 62](#_Toc200204546)

[Figure S12 Risk of overall survival in studies with a lower percentage of HBV-infected patients (n=12 studies) 63](#_Toc200204547)

[Table S16 Risk of overall survival in studies with a lower percentage of HBV-infected patients (n=12 studies) 63](#_Toc200204548)

[Figure S13 Risk of overall survival in studies with a higher percentage of HBV-infected patients (n=12 studies) 64](#_Toc200204549)

[Table S17 Risk of overall survival in studies with a higher percentage of HBV-infected patients (n=12 studies) 64](#_Toc200204550)

[Figure S14 Distribution of the percentage of HCV-infected patients across OS 65](#_Toc200204551)

[Figure S15 Risk of overall survival in studies with a lower percentage of HCV-infected patients (n=7 studies) 66](#_Toc200204552)

[Table S18 Risk of overall survival in studies with a lower percentage of HCV-infected patients (n=7 studies) 66](#_Toc200204553)

[Figure S16 Risk of overall survival in studies with a higher percentage of HCV-infected patients (n=7 studies) 67](#_Toc200204554)

[Table S18 Risk of overall survival in studies with a higher percentage of HCV-infected patients (n=7 studies) 67](#_Toc200204555)

[Figure S16 Distribution of the percentage of patients with tumor number ≥3 68](#_Toc200204556)

[Figure S17 Risk of overall survival in studies with a higher percentage of tumor numbers≥3 (n=6 studies) 69](#_Toc200204557)

[Table S19 Risk of overall survival in studies with a higher percentage of tumor number ≥3 (n=6 studies) 69](#_Toc200204558)

[Figure S18 Risk of overall survival in studies with a lower percentage of tumor number ≥3 (n=6 studies) 70](#_Toc200204559)

[Table S20 Risk of overall survival in studies with a lower percentage of tumor number ≥3 (n=6 studies) 70](#_Toc200204560)

[Figure S19 Distribution of the tumor size 71](#_Toc200204561)

[Figure s20 Risk of overall survival in studies with median tumor size ＜6 cm (n=5 studies) 72](#_Toc200204562)

[Table S21 Risk of overall survival in studies with median tumor size ＜6 cm (n=5 studies) 72](#_Toc200204563)

[Figure S21 Risk of overall survival in studies with median tumor size ≥ 6 cm (n= 5 studies) 73](#_Toc200204564)

[Table S22 Risk of overall survival in studies with median tumor size ≥ 6 cm (n=5 studies) 73](#_Toc200204565)

[Figure S22: Distribution of the sample size of included studies 74](#_Toc200204566)

[Figure s23 Risk of overall survival in studies with median sample size ＜88 (n = 13 studies) 75](#_Toc200204567)

[Table S23 Risk of overall survival in studies with median sample size ＜88 (n = 13 studies) 75](#_Toc200204568)

[Figure S24: Risk of overall survival in studies with median sample size ≥88 (n = 13 studies) 76](#_Toc200204569)

[Table S24 Risk of overall survival in studies with median sample size ≥88 (n = 13 studies) 76](#_Toc200204570)

**Table s1 PRISMA NMA Checklist of Items to Include When Reporting A Systematic Review Involving a Network Meta-analysis**

| **Section/Topic** | **Item #** | **Checklist Item** | **Reported on Page #** |
| --- | --- | --- | --- |
| **TITLE** |  |  |  |
| Title | 1 | Identify the report as a systematic review *incorporating a network meta-analysis (or related form of meta-analysis).* | 1 |
| **ABSTRACT** |  |  |  |
| Structured summary | 2 | Provide a structured summary including, as applicable:  **Background:** main objectives  **Methods:** data sources; study eligibility criteria, participants, and interventions; study appraisal; and *synthesis methods, such as network meta-analysis.*  **Results:** number of studies and participants identified; summary estimates with corresponding confidence/credible intervals; *treatment rankings may also be discussed. Authors may choose to summarize pairwise comparisons against a chosen treatment included in their analyses for brevity.*  **Discussion/Conclusions:** limitations; conclusions and implications of findings.  **Other:** primary source of funding; systematic review registration number with registry name. | 1 |
| **INTRODUCTION** |  |  |  |
| Rationale | 3 | Describe the rationale for the review in the context of what is already known*, including mention of why a network meta-analysis has been conducted.* | 3 |
| Objectives | 4 | Provide an explicit statement of questions being addressed, with reference to participants, interventions, comparisons, outcomes, and study design (PICOS). | 3 |
|  |  |  |  |
| **METHODS** |  |  |  |
| Protocol and registration | 5 | Indicate whether a review protocol exists and if and where it can be accessed (e.g., Web address); and, if available, provide registration information, including registration number. | 4 |
| Eligibility criteria | 6 | Specify study characteristics (e.g., PICOS, length of follow-up) and report characteristics (e.g., years considered, language, publication status) used as criteria for eligibility, giving rationale. *Clearly describe eligible treatments included in the treatment network, and note whether any have been clustered or merged into the same node (with justification).* | 4 |
| Information sources | 7 | Describe all information sources (e.g., databases with dates of coverage, contact with study authors to identify additional studies) in the search and date last searched. | 4 |
| Search | 8 | Present full electronic search strategy for at least one database, including any limits used, such that it could be repeated. | Appendix 1.2 |
| Study selection | 9 | State the process for selecting studies (i.e., screening, eligibility, included in systematic review, and, if applicable, included in the meta-analysis). | 4-5 |
| Data collection process | 10 | Describe method of data extraction from reports (e.g., piloted forms, independently, in duplicate) and any processes for obtaining and confirming data from investigators. | 4-5 |
| Data items | 11 | List and define all variables for which data were sought (e.g., PICOS, funding sources) and any assumptions and simplifications made. | 4-5 |
| **Geometry of the network** | **S1** | Describe methods used to explore the geometry of the treatment network under study and potential biases related to it. This should include how the evidence base has been graphically summarized for presentation, and what characteristics were compiled and used to describe the evidence base to readers. | 5 |
| Risk of bias within individual studies | 12 | Describe methods used for assessing risk of bias of individual studies (including specification of whether this was done at the study or outcome level), and how this information is to be used in any data synthesis. | 5 |
| Summary measures | 13 | State the principal summary measures (e.g., risk ratio, difference in means). *Also describe the use of additional summary measures assessed, such as treatment rankings and surface under the cumulative ranking curve (SUCRA) values, as well as modified approaches used to present summary findings from meta-analyses.* | 5 |
| Planned methods of analysis | 14 | Describe the methods of handling data and combining results of studies for each network meta-analysis. This should include, but not be limited to:   - *Handling of multi-arm trials;* - *Selection of variance structure;* - *Selection of prior distributions in Bayesian analyses; and* - *Assessment of model fit.* | 5 |
| **Assessment of Inconsistency** | **S2** | Describe the statistical methods used to evaluate the agreement of direct and indirect evidence in the treatment network(s) studied. Describe efforts taken to address its presence when found. | 5-6 |
| Risk of bias across studies | 15 | Specify any assessment of risk of bias that may affect the cumulative evidence (e.g., publication bias, selective reporting within studies). | 5-6 |
| Additional analyses | 16 | Describe methods of additional analyses if done, indicating which were pre-specified. This may include, but not be limited to, the following:   - Sensitivity or subgroup analyses; - Meta-regression analyses; - *Alternative formulations of the treatment network; and* - *Use of alternative prior distributions for Bayesian analyses (if applicable).* | 6 |
| **RESULTS†** |  |  |  |
| Study selection | 17 | Give numbers of studies screened, assessed for eligibility, and included in the review, with reasons for exclusions at each stage, ideally with a flow diagram. | 7 Figure 1 |
| **Presentation of network structure** | **S3** | Provide a network graph of the included studies to enable visualization of the geometry of the treatment network. | Figure 2/3 |
| **Summary of network geometry** | **S4** | Provide a brief overview of characteristics of the treatment network. This may include commentary on the abundance of trials and randomized patients for the different interventions and pairwise comparisons in the network, gaps of evidence in the treatment network, and potential biases reflected by the network structure. | 7 |
| Study characteristics | 18 | For each study, present characteristics for which data were extracted (e.g., study size, PICOS, follow-up period) and provide the citations. | Appendix 2.1 |
| Risk of bias within studies | 19 | Present data on risk of bias of each study and, if available, any outcome level assessment. | 7, Appendix 2.2 |
| Results of individual studies | 20 | For all outcomes considered (benefits or harms), present, for each study: 1) simple summary data for each intervention group, and 2) effect estimates and confidence intervals. *Modified approaches may be needed to deal with information from larger networks.* | Appendix 2.3 |
| Synthesis of results | 21 | Present results of each meta-analysis done, including confidence/credible intervals. *In larger networks, authors may focus on comparisons versus a particular comparator (e.g. placebo or standard care), with full findings presented in an appendix. League tables and forest plots may be considered to summarize pairwise comparisons.* If additional summary measures were explored (such as treatment rankings), these should also be presented. | 7-14,  Appendix 2.4  Figure 2/3 |
| **Exploration for inconsistency** | **S5** | Describe results from investigations of inconsistency. This may include such information as measures of model fit to compare consistency and inconsistency models, *P* values from statistical tests, or summary of inconsistency estimates from different parts of the treatment network. | Appendix  2.5 |
| Risk of bias across studies | 22 | Present results of any assessment of risk of bias across studies for the evidence base being studied. | Appendix  2.5 |
| Results of additional analyses | 23 | Give results of additional analyses, if done (e.g., sensitivity or subgroup analyses, meta-regression analyses*, alternative network geometries studied, alternative choice of prior distributions for Bayesian analyses,* and so forth). | 7-14; Figure s1; Appendix |
| **DISCUSSION** |  |  |  |
| Summary of evidence | 24 | Summarize the main findings, including the strength of evidence for each main outcome; consider their relevance to key groups (e.g., healthcare providers, users, and policy-makers). | 15-18 |
| Limitations | 25 | Discuss limitations at study and outcome level (e.g., risk of bias), and at review level (e.g., incomplete retrieval of identified research, reporting bias). *Comment on the validity of the assumptions, such as transitivity and consistency. Comment on any concerns regarding network geometry (e.g., avoidance of certain comparisons).* | 18 |
| Conclusions | 26 | Provide a general interpretation of the results in the context of other evidence, and implications for future research. | 18 |
| **FUNDING** |  |  |  |
| Funding | 27 | Describe sources of funding for the systematic review and other support (e.g., supply of data); role of funders for the systematic review. This should also include information regarding whether funding has been received from manufacturers of treatments in the network and/or whether some of the authors are content experts with professional conflicts of interest that could affect use of treatments in the network. | 18 |

PICOS = population, intervention, comparators, outcomes, study design.

**Table s2 PRISMA checklist for NMA abstract**

| **Section and Topic** | **Item**  **#** | **Checklist item** | **Reported**  **(Yes/No)** |
| --- | --- | --- | --- |
| **Title** | | | |
| Title | 1 | Identify the report as a systematic review. | Yes |
| **Background** | | | |
| Objectives | 2 | Provide an explicit statement of the main objective(s) or question(s) the review addresses | Yes |
| **Methods** | | | |
| Eligibility criteria | 3 | Specify the inclusion and exclusion criteria for the review | Yes |
| Information sources | 4 | Specify the information sources (e.g. databases, registers) used to identify studies and the date when each was last searched | Yes |
| Risk of bias | 5 | Specify the methods used to assess risk of bias in the included studies. | Yes |
| Synthesis of results | 6 | Specify the methods used to present and synthesise results. | Yes |
| **Results** | | | |
| Included studies | 7 | Give the total number of included studies and participants and summarise relevant characteristics of studies. | Yes |
| Synthesis of results | 8 | Present results for main outcomes, preferably indicating the number of included studies and participants for each. If meta-analysis was done, report the summary estimate and confidence/credible interval. If comparing groups, indicate the direction of the effect (i.e. which group is favoured). | Yes |
| **Discussion** | | | |
| Limitations of evidence | 9 | Provide a brief summary of the limitations of the evidence included in the review (e.g. study risk of bias, inconsistency and imprecision). | Yes |
| Interpretation | 10 | Provide a general interpretation of the results and important implications. | Yes |
| **Other** | | | |
| Funding | 11 | Specify the primary source of funding for the review. | Yes |
| Registration | 12 | Provide the register name and registration number. | Yes |

**Table s3 Seach Strategy**

**PubMed**

#1 HCC

((((((((liver neoplasms[MeSH Terms]) OR (hepatocellular carcinoma[Title/Abstract])) OR (Hepatic Cancer[Title/Abstract])) OR (Liver Cancer[Title/Abstract])) OR (liver cell carcinoma[Title/Abstract])) OR (primary liver cancer[Title/Abstract])) OR (liver cancer[Title/Abstract])) OR (hepatoma[Title/Abstract])) OR (PLC[Title/Abstract])

#2 TACE

(((embolization[Title/Abstract]) OR (chemoembolization[Title/Abstract])) OR (TACE[Title/Abstract])) OR (TAE[Title/Abstract])

#3 BCLC-B stage

(((((BCLC B[Title/Abstract]) OR (intermediate[Title/Abstract])) OR (BCLC stage B[Title/Abstract])) OR (Barcelona Clinic Liver Cancer stage B[Title/Abstract])) OR (stage b[Title/Abstract])) OR (middle stage[Title/Abstract])

#4 Review

(review[Filter]) OR (meta analysis[Filter])

#1 AND #2 AND #3 NOT #4

**Embase**

#16 #11 AND #14 AND #15

#15 'bclc b':ab,ti OR intermediate:ab,ti OR 'bclc stage b':ab,ti OR 'barcelona clinic liver cancer stage b':ab,ti OR 'stage b':ab,ti OR 'middle stage':ab,ti

#14 #12 OR #13

#13 'tace':ab,ti OR 'chemoembolization':ab,ti OR 'tae':ab,ti OR 'embolization':ab,ti

#12 'chemoembolization'/exp

#11 #3 OR #10

#10 #6 AND #9

#9 #7 OR #8

#8 'liver'/exp

#7 'liver*':ab,ti OR 'hepatocellular':ab,ti OR 'hepatic':ab,ti

#6 #4 OR #5

#5 'carcinoma*':ab,ti OR 'cancer':ab,ti OR 'tumor':ab,ti OR 'neoplasia*':ab,ti OR 'neoplasm*':ab,ti OR 'epithelioma*':ab,ti

#4 'carcinoma'/exp OR 'carcinoma' OR 'neoplasm'/exp OR 'neoplasm'

#3 #1 OR #2

#2 'liver tumor':ab,ti OR 'liver cancer':ab,ti OR 'liver cell carcinoma':ab,ti OR 'liver neoplasm':ab,ti OR 'hepatocellular carcinoma':ab,ti OR 'hepatic cancer':ab,ti OR 'plc':ab,ti OR 'hcc':ab,ti

#1 'liver tumor'/exp OR 'liver tumor' OR 'hepatobiliary system cancer'/exp OR 'hepatobiliary system cancer'

**Cochrane**

#1 (liver*):ti,ab,kw

#2 (hepatocellular):ti,ab,kw

#3 (hepatic*):ti,ab,kw

#4 #1 OR #2 OR #3

#5 (neoplasm*):ti,ab,kw

#6 (cancer*):ti,ab,kw

#7 (carcinoma*):ti,ab,kw

#8 (tumor*):ti,ab,kw

#9 #5 OR #6 OR #7 OR #8

#10 #4 AND #9

#11 MeSH descriptor: [Liver Neoplasms] explode all trees

#12 MeSH descriptor: [Carcinoma, Hepatocellular] explode all trees

#13 ("liver neoplasm"):ti,ab,kw

#14 (neoplasm, liver):ti,ab,kw

#15 (hepatic neoplasm):ti,ab,kw

#16 (neoplasm, hepatic):ti,ab,kw

#17 (Cancer of Liver):ti,ab,kw

#18 (Hepatocellular Cancer):ti,ab,kw

#19 (Cancers, Hepatocellular):ti,ab,kw

#20 (Hepatocellular Cancers):ti,ab,kw

#21 (Hepatic Cancer):ti,ab,kw

#22 (Cancer, Hepatic):ti,ab,kw

#23 (Cancers, Hepatic):ti,ab,kw

#24 (Hepatic Cancers):ti,ab,kw

#25 (Liver Cancer):ti,ab,kw

#26 (Cancer, Liver):ti,ab,kw

#27 (Cancer, Hepatocellular):ti,ab,kw

#28 #10 OR #11 OR #12 OR #13 OR #14 OR #15 OR #16 OR #17 OR #18 OR #19 OR #20 OR #21 OR #22 OR #23 OR #24 OR #25 OR #26

#29 (HCC):ti,ab,kw

#30 (PLC):ti,ab,kw

#31 #10 OR #28 OR #29 OR #30

#32 (Chemoembolization*):ti,ab,kw

#33 (TACE):ti,ab,kw

#34 (TAE):ti,ab,kw

#35 #32 OR #33 OR #34

#36 (BCLC B):ti,ab,kw

#37 (intermediate):ti,ab,kw

#38 (BCLC stage B):ti,ab,kw

#39 (Barcelona Clinic Liver Cancer stage B):ti,ab,kw

#40 (stage b):ti,ab,kw

#41 (middle stage):ti,ab,kw

#42 #36 OR #37 OR #38 OR #39 OR #40 OR #41

#43 #31 AND #35 AND #42

**Table s4 Risk of bias in studies reporting on risk of OS**

| **Study** | **RoB due to confounding (for RCTs this domain assessed the randomization process) (Part 1)** | **RoB in selection of participants (Part 2) (only for NRS)** | **RoB in classification of interventions (Part 3) (only for NRS)** | **RoB due to deviation from intended interventions (Part 4)** | **RoB due to missing data (Part 5)** | **RoB in measurement of the outcome (Part 6)** | **RoB in selection of reported results (Part 7)** | **Overall RoB** |
| --- | --- | --- | --- | --- | --- | --- | --- | --- |
| **Zhou ^[1]^** | - 1. Y; 1.2 N; 1.4 Y; 1.5 Y; 1.6 PN   Low RoB  (Adjustment for age, gender, etiology (HBV/HCV infected), tumor size, tumor number and AFP using propensity score matching, liver function was classified as CP-A) | 2.1 N; 2.4 Y  Low RoB | 3.1 Y; 3.2 Y; 3.3 NI  Low RoB | 4.1 N  Low RoB | 5.1 Y; 5.2 N; 5.3 N  Low RoB | 6.1 NI; 6.2 NI; 6.3 Y; 6.4 N  NI about RoB  (NI abut method of ascertainment of outcome) | 7.1 N; 7.2 N; 7.3 N  Low RoB | Low |
| **Lencioni 2016 ^[2]^** | - 1. NI, 1.2 NI, 1.3 N   Some concerns  (Method of randomization not reported) | **-** | **-** | 4.1 N, 4.2 N, 4.6 Y  Low RoB | 5.1 Y  Low RoB | 6.1 N, 6.2 N, 6.3 N  Low RoB | 7.1 NI, 7.2 N, 7.3 N  Some concerns | Moderate |
| **Kim 2022 ^[3]^** | - 1. Y; 1.2 N; 1.4 Y; 1.5 Y; 1.6 PN   Low RoB  (Adjustment for age, gender, etiology (HBV/HCV infected), tumor size, tumor number and AFP using propensity score matching, no control for Child-Pugh class) | 2.1 N; 2.4 Y  Low RoB | 3.1 Y; 3.2 Y; 3.3 NI  Low RoB | 4.1 N  Low RoB | 5.1 Y; 5.2 N; 5.3 N  Low RoB | 6.1 NI; 6.2 NI; 6.3 Y; 6.4 N  NI about RoB  (NI abut method of ascertainment of outcome) | 7.1 N; 7.2 N; 7.3 N  Low RoB | Low |
| **Endo 2018 ^[4]^** | - 1. Y; 1.2 N; 1.4 Y; 1.5 Y; 1.6 PN   Low RoB  (Adjustment for age, gender, etiology (HBV/HCV infected), child-Pugh class, tumor size, tumor number and AFP using propensity score matching) | 2.1 N; 2.4 Y;  Low RoB | 3.1 Y; 3.2 Y; 3.3 NI  Low RoB | 4.1 N  Low RoB | 5.1 Y; 5.2 N; 5.3 N  Low RoB | 6.1 N; 6.2 NI; 6.3 Y; 6.4 N  Low RoB | 7.1 N; 7.2 N; 7.3 N  Low RoB | Low |
| **Lin 2020 ^[5]^** | - 1. Y; 1.2 N; 1.4 PY; 1.5 PY; 1.6 N   Low RoB  (Adjustment for age, gender, etiology (HBV/HCV infected), Child-Pugh class, tumor size and tumor number with propensity score matching analysis) | 2.1 N; 2.4 Y  Low RoB | 3.1 Y; 3.2 Y; 3.3 N  Low RoB | 4.1 N  Low RoB | 5.1 Y; 5.2 N; 5.3 N  Low RoB | 6.1 NI; 6.2 NI; 6.3 Y; 6.4 N  NI about RoB | 7.1 N; 7.2 N; 7.3 N  Low RoB | Low |
| **Hirooka**  **2018 ^[6]^** | - 1. Y; 1.2 N; 1.4 Y; 1.5 PY; 1.6 N   Low RoB  (Adjustment for age, sex, etiology (HBV/HCV infected), child-Pugh class, tumor size and tumor number with propensity score matching analysis) | 2.1 N; 2.4 Y  Low RoB | 3.1 Y; 3.2 Y;  3.3 NI  NI about RoB | 4.1 N  Low RoB | 5.1 Y; 5.2 N; 5.3 N  Low RoB | 6.1 NI; 6.2 NI; 6.3 Y; 6.4 N  NI about RoB | 7.1 N; 7.2 N; 7.3 N  Low RoB | NI (Probably low) |
| **Nouso 2017 ^[7]^** | 1.1 Y; 1.2 N; 1.4 Y; 1.5 PY; 1.6 N  Low RoB  (Adjustment for age, gender, etiology, child-pugh class, tumor size, tumor number, AFP with propensity score matching analysis) | 2.1 N; 2.4 Y  Low RoB | 3.1 Y; 3.2 Y; 3.3 N  Low RoB | 4.1 N  Low RoB | 5.1 Y; 5.2 NI; 5.3 NI  NI about RoB | 6.1 N; 6.2 NI; 6.3 Y; 6.4 N  Low RoB | 7.1 N; 7.2 N; 7.3 N | Low |
| **Peng 2019 ^[8]^** | - 1. Y; 1.2 N; 1.4 Y;   1.5 PY; 1.6 N  Low RoB  (Propensity score matching (PSM) was conducted to minimize effect of confounding factors, including age, gender, etiology (HBV infected), CT class, AFP, tumor number, tumor size) | 2.1 N; 2.4 Y  Low RoB | 3.1 Y; 3.2 Y; 3.3 N  Low RoB | 4.1 N  Low RoB | 5.1 Y; 5.2 NI;  5.3 NI  NI about RoB | 6.1 N; 6.2 NI; 6.3 Y; 6.4 N  Low RoB | 7.1 N; 7.2 N; 7.3 N  Low RoB | NI (probably low) |
| **Ke 2014 ^[9]^** | - 1. Y; 1.2 N; 1.4 Y   1.5 PY; 1.6 N  Low RoB  (Adjustment for age, gender, etiology (HBV infected), AFP, tumor size, tumor number with propensity score matching, no control for CT class) | 2.1 N; 2.4 Y  Low RoB | 3.1 Y; 3.2 Y; 3.3 N  Low RoB | 4.1 N  Low RoB | 5.1 Y; 5.2 NI;  5.3 N  Low RoB | 6.1 N; 6.2 NI; 6.3 Y; 6.4 N  Low RoB | 7.1 N; 7.2 N; 7.3 N  Low RoB | Low |
| **Chen 2020 ^[10]^** | - 1. Y; 1.2 N; 1.4 NI; 1.6 N   NI about RoB (probably serious due to the HR and relative 95% CI were obtained in patients before PSM) | 2.1 N; 2.4 Y  Low RoB | 3.1 Y; 3.2 Y;  3.3 N  Low RoB | 4.1 N  Low RoB | 5.1 Y; 5.2 NI; 5.3 NI  NI about RoB | 6.1 N; 6.2 NI; 6.3 Y; 6.4 N  Low RoB | 7.1 N; 7.2 N; 7.3 N  Low RoB | NI (probably serious) |
| **Tada 2017 ^[11]^** | - 1. Y; 1.2 N; 1.4 PY   1.5 Y; 1.6 N  Low RoB  (Adjustment for age/sex/etiology/chilid-pugh score/tumor numbers/tumor size/AFP using propensity score matching analysis-(PSM)) | 2.1 N; 2.4 Y  Low RoB | 3.1 Y; 3.2 Y;  3.3 N  Low RoB | 4.1 N  Low RoB | 5.1 Y; 5.2 NI; 5.3 NI  NI about RoB | 6.1 N; 6.2 NI; 6.3 Y; 6.4 N  Low RoB | 7.1 N; 7.2 N; 7.3 N  Low RoB | Low |
| **Espinosa 2018 ^[12]^** | 1.1 Y; 1.2 N; 1.4 PY; 1.5 Y; 1.6 N  Low RoB  (a propensity-matched analysis was performed to adjust for age, gender, etiology (HBV/HCV infected), AFP, child-pugh class, tumor number and tumor size) | 2.1 N; 2.4 Y  Low RoB | 3.1 Y; 3.2 Y;  3.3 N  Low RoB | 4.1 N  Low RoB | 5.1 Y; 5.2 NI; 5.3 NI  NI about RoB | 6.1 N; 6.2 NI; 6.3 Y; 6.4 N  Low RoB | 7.1 N; 7.2 N; 7.3 N  Low RoB | Low |
| **Hou 2016 ^[13]^** | - 1. Y; 1.2 N; 1.4   PY; 1.5 NI; 1.6 N  Low RoB  (Age/gender/etiology (HBV/HCV infected)/AFP/tumor number/tumor size did not differ between groups, no control for CT class) | 2.1 N; 2.4 Y  Low RoB | 3.1 Y; 3.2 Y;  3.3 NI  NI about RoB | 4.1 N  Low RoB | 5.1 Y; 5.2 NI; 5.3 NI  NI about RoB | 6.1 N; 6.2 NI; 6.3 Y; 6.4 N  Low RoB | 7.1 N; 7.2 N; 7.3 N  Low RoB | Low |
| **Akarapatima 2022 ^[14]^** | - 1. Y; 1.2 N; 1.4 N; 1.6 N   Moderate RoB  (Age, gender, etiology (HBV infected), tumor size, AFP level did not differ between groups, expect for HCV infected; no control for CT class) | 2.1 N; 2.4 Y  Low RoB | 3.1 Y; 3.2 Y;  3.3 N  Low RoB | 4.1 N  Low RoB | 5.1 Y; 5.2 NI; 5.3 N  NI about RoB | 6.1 N; 6.2 NI; 6.3 Y; 6.4 N  NI about RoB | 7.1 N; 7.2 N; 7.3 N  Low RoB | Moderate |
| **Kariyama 2020 ^[15]^** | 1.1 Y; 1.2 N; 1.4 PY; 1.5 Y; 1.6 N  Moderate RoB  (A propensity score-matching analysis: was performed to adjust age, gender, etiology, tumor size and AFP level, no control for tumor number, CT class) | 2.1 N; 2.4 Y  Low RoB | 3.1 Y; 3.2 Y;  3.3 N  Low RoB | 4.1 N  Low RoB | 5.1 Y; 5.2 NI; 5.3 N  NI about RoB | 6.1 N; 6.2 NI; 6.3 Y; 6.4 N  NI about RoB | 7.1 N; 7.2 N; 7.3 N  Low RoB | Moderate |
| **Kim 2016 ^[16]^** | 1.1 Y; 1.2 N; 1.4 PY; 1.5 PY; 1.6 N  Serious RoB  (Age, Child-Pugh class, tumor number differs between the two groups) | 2.1 N; 2.4 Y  Low RoB | 3.1 Y; 3.2 Y;  3.3 N  Low RoB | 4.1 N  Low RoB | 5.1 Y; 5.2 Y; 5.3 Y; 5.4 Y; 5.5 PY  Moderate RoB | 6.1 N; 6.2 NI; 6.3 Y; 6.4 N  Low RoB | 7.1 N; 7.2 N; 7.3 N  Low RoB | Serious |
| **Ohama 2022 ^[17]^** | 1.1 Y; 1.2 N; 1.4 PY; 1.5 NI; 1.6 N  Low RoB  (Age, gender, etiology, tumor number, tumor size, AFP level didn’t differ between groups) | 2.1 N; 2.4 Y  Low RoB | 3.1 Y; 3.2 Y;  3.3 NI  NI about RoB | 4.1 N  Low RoB | 5.1 Y; 5.2 NI; 5.3 NI  NI about RoB  (Probably low) | 6.1 N; 6.2 NI; 6.3 Y; 6.4 N  Low RoB | 7.1 N; 7.2 N; 7.3 N  Low RoB | Low |
| **Zhou 2020 ^[18]^** | 1.1 Y; 1.2 N; 1.4 PY; 1.5 Y; 1.6 N  Moderate RoB  (Age, gender, tumor number, HBV infected, AFP level didn’t differ between groups, tumor size marginally statical differed, no control for Child-Pugh class) | 2.1 N; 2.4 Y  Low RoB | 3.1 Y; 3.2 Y;  3.3 N  Low RoB | 4.1 N  Low RoB | 5.1 Y; 5.2 NI; 5.3 N  NI about RoB | 6.1 N; 6.2 NI; 6.3 Y; 6.4 N  Low RoB | 7.1 N; 7.2 N; 7.3 N  Low RoB | Moderate |
| **Yang 2022 ^[19]^** | 1.1 Y; 1.2 N; 1.4 PY; 1.5 Y; 1.6 N  Low RoB  (Adjustment for age, gender, etiology (HBV/HCV infected), Child-Pugh scores, AFP level, tumor size, tumor number with propensity score match analysis) | 2.1 N; 2.4 Y  Low RoB | 3.1 Y; 3.2 Y;  3.3 N  Low RoB | 4.1 N  Low RoB | 5.1 Y; 5.2 NI; 5.3 N  NI about RoB | 6.1 N; 6.2 NI; 6.3 Y; 6.4 N  Low RoB | 7.1 N; 7.2 N; 7.3 N  Low RoB | Low |
| **Wang 2023 ^[20]^** | 1.1 Y; 1.2 N; 1.4 PY; 1.5 NI; 1.6 N  Low RoB  (Age, gender, etiology, tumor number, tumor size, AFP level didn’t differ between groups; no control for Child-Pugh class) | 2.1 N; 2.4 Y  Low RoB | 3.1 Y; 3.2 Y;  3.3 NI  NI about RoB | 4.1 N  Low RoB | 5.1 Y; 5.2 NI; 5.3 PY; 5.4 NI; 5.5 NI  NI about RoB  (Probably moderate) | 6.1 N; 6.2 NI; 6.3 Y; 6.4 N  Low RoB | 7.1 N; 7.2 N; 7.3 N  Low RoB | Moderate |
| **Wang 2021 ^[21]^** | 1.1 Y; 1.2 N; 1.4 PY; 1.5 Y; 1.6 N  Low RoB  (Adjustment for age, gender, HBV infected, AFP level, tumor size, tumor number with propensity score match analysis, expect for CT class) | 2.1 N; 2.4 Y  Low RoB | 3.1 Y; 3.2 Y;  3.3 N  Low RoB | 4.1 N  Low RoB | 5.1 Y; 5.2 NI; 5.3 N  NI about RoB | 6.1 N; 6.2 NI; 6.3 Y; 6.4 N  Low RoB | 7.1 N; 7.2 N; 7.3 N  Low RoB | Low |
| **Nong 2023 ^[22]^** | 1.1 Y; 1.2 N; 1.4 PY; 1.5 NI; 1.6 N  Moderate RoB  (Age, gender, AFP level, HBV infection didn’t differ between groups; tumor number significantly differs between the two groups, no control for tumor size, Child-Pugh class) | 2.1 N; 2.4 Y  Low RoB | 3.1 Y; 3.2 Y;  3.3 N  Low RoB | 4.1 N  Low RoB | 5.1 Y; 5.2 NI; 5.3 N  NI about RoB (Moderate) | 6.1 N; 6.2 NI; 6.3 Y; 6.4 N  Low RoB | 7.1 N; 7.2 N; 7.3 N  Low RoB | Moderate |
| **Hu 2024 ^[23]^** | 1.1 Y; 1.2 N; 1.4 PY; 1.5 NI; 1.6 N  low RoB  (Age, gender, AFP level, tumor size, tumor number, HBV infection didn’t differ between groups; no control for Child-Pugh class) | 2.1 N; 2.4 Y  Low RoB | 3.1 Y; 3.2 Y;  3.3 NI  NI about RoB | 4.1 N  Low RoB | 5.1 Y; 5.2 NI; 5.3 NI  NI about RoB  (Probably low) | 6.1 N; 6.2 NI; 6.3 Y; 6.4 N  Low RoB | 7.1 N; 7.2 N; 7.3 N  Low RoB | Low |
| **Zhu 2023 ^[24]^** | 1.1 Y; 1.2 N; 1.4 PY; 1.5 NI; 1.6 N  low RoB  (Age, gender, AFP level, tumor size, tumor number, HBV infection, Child-Pugh class didn’t differ between groups) | 2.1 N; 2.4 Y  Low RoB | 3.1 Y; 3.2 Y;  3.3 NI  NI about RoB | 4.1 N  Low RoB | 5.1 Y; 5.2 NI; 5.3 NI  NI about RoB  (Probably low) | 6.1 N; 6.2 NI; 6.3 Y; 6.4 N  Low RoB | 7.1 N; 7.2 N; 7.3 N  Low RoB | Low |
| **Huang 2023 ^[25]^** | 1.1 Y; 1.2 N; 1.4 NI; 1.6 N  High RoB  (No control for confounding factors) | 2.1 N; 2.4 Y  Low RoB | 3.1 Y; 3.2 Y;  3.3 N  Low RoB | 4.1 N  Low RoB | 5.1 Y; 5.2 NI; 5.3 N  NI about RoB | 6.1 N; 6.2 NI; 6.3 Y; 6.4 N  Low RoB | 7.1 N; 7.2 N; 7.3 N  Low RoB | High |
| **Li 2023 ^[26]^** | 1.1 Y; 1.2 N; 1.4 PY; 1.5 NI; 1.6 N  low RoB  (Age, gender, AFP level, tumor size, tumor number, HBV infection, Child-Pugh class didn’t differ between groups) | 2.1 N; 2.4 Y  Low RoB | 3.1 Y; 3.2 Y;  3.3 N  Low RoB | 4.1 N  Low RoB | 5.1 Y; 5.2 NI; 5.3 N  NI about RoB | 6.1 N; 6.2 NI; 6.3 Y; 6.4 N  Low RoB | 7.1 N; 7.2 N; 7.3 N  Low RoB | Low |
| **Chen 2019 ^[27]^** | 1.1 Y; 1.2 N; 1.4 PY; 1.5 NI; 1.6 N  low RoB  (Age, gender, AFP level, tumor size, tumor number, HBV infection, Child-Pugh class didn’t differ between groups) | 2.1 N; 2.4 Y  Low RoB | 3.1 Y; 3.2 Y;  3.3 NI  NI about RoB | 4.1 N  Low RoB | 5.1 Y; 5.2 NI; 5.3 NI  NI about RoB  (Probably low) | 6.1 N; 6.2 NI; 6.3 Y; 6.4 N  Low RoB | 7.1 N; 7.2 N; 7.3 N  Low RoB | Low |
| **Yan 2020 ^[28]^** | 1.1 Y; 1.2 N; 1.4 PY; 1.5 NI; 1.6 N  low RoB  (Age, gender, AFP level, tumor size, tumor number, HBV infection, Child-Pugh class didn’t differ between groups) | 2.1 N; 2.4 Y  Low RoB | 3.1 Y; 3.2 Y;  3.3 NI  NI about RoB | 4.1 N  Low RoB | 5.1 Y; 5.2 NI; 5.3 NI  NI about RoB  (Probably low) | 6.1 N; 6.2 NI; 6.3 Y; 6.4 N  Low RoB | 7.1 N; 7.2 N; 7.3 N  Low RoB | Low |
| **Kudo 2018^[29]^** | - 1. Y, 1.2 Y, 1.3 N   Low RoB | **-** | **-** | 4.1 N, 4.2 N; 4.6 Y  Low RoB | 5.1 Y  Low RoB | 6.1 N, 6.2 N, 6.3 NI  NI about RoB  (Probably moderate) | 7.1 NI, 7.2 N, 7.3 N  NI about RoB  (Probably moderate) | Moderate |
| **Lu 2021^[30]^** | 1.1 Y; 1.2 N; 1.4 PY; 1.5 NI; 1.6 N  Low RoB  (Adjustment for age, the sum of tumor number and tumor size, AFP level using propensity score-matched analysis, gender, etiology, Child-Pugh class didn’t differ between groups) | 2.1 N; 2.4 Y  Low RoB | 3.1 Y; 3.2 Y;  3.3 NI  NI about RoB | 4.1 N  Low RoB | 5.1 Y; 5.2 NI; 5.3 PY; 5.4 NI; 5.5 NI  NI about RoB  (Probably moderate) | 6.1 N; 6.2 NI; 6.3 Y; 6.4 N  Low RoB | 7.1 N; 7.2 N; 7.3 N  Low RoB | Low |
| **Luo 2011 ^[31]^** | 1.1 Y; 1.2 N; 1.4 PY; 1.5 NI; 1.6 N  Low RoB  (Age, AFP level, tumor size, tumor number, HBV infection, Child-Pugh class didn’t differ between groups, expect for gender) | 2.1 N; 2.4 Y  Low RoB | 3.1 Y; 3.2 Y;  3.3 NI  NI about RoB | 4.1 N  Low RoB | 5.1 Y; 5.2 NI; 5.3 PY; 5.4 NI; 5.5 NI  NI about RoB  (Probably moderate) | 6.1 N; 6.2 NI; 6.3 Y; 6.4 N  Low RoB | 7.1 N; 7.2 N; 7.3 N  Low RoB | Low |
| **Xu 2018 ^[32]^** | 1.1 Y; 1.2 N; 1.4 PY; 1.5 NI; 1.6 N  Moderate RoB  (Age, AFP level, tumor size, tumor number, HBV infection, Child-Pugh class didn’t differ between groups, expect for tumor number) | 2.1 N; 2.4 Y  Low RoB | 3.1 Y; 3.2 Y;  3.3 NI  NI about RoB | 4.1 N  Low RoB | 5.1 Y; 5.2 NI; 5.3 PY; 5.4 NI; 5.5 NI  NI about RoB  (Probably moderate) | 6.1 N; 6.2 NI; 6.3 Y; 6.4 N  Low RoB | 7.1 N; 7.2 N; 7.3 N  Low RoB | Moderate |
| **Ciria 2015 ^[33]^** | 1.1 Y; 1.2 N; 1.4 PY; 1.5 NI; 1.6 N  High RoB  (Age, gender, HBV infection, Child-Pugh class didn’t differ between groups, expect for tumor number, main tumor size, Child-Pugh class) | 2.1 N; 2.4 Y  Low RoB | 3.1 Y; 3.2 Y;  3.3 NI  NI about RoB | 4.1 N  Low RoB | 5.1 Y; 5.2 NI; 5.3 PY; 5.4 NI; 5.5 NI  NI about RoB  (Probably moderate) | 6.1 N; 6.2 NI; 6.3 Y; 6.4 N  Low RoB | 7.1 N; 7.2 N; 7.3 N  Low RoB | High |
| **Yin 2014 ^[34]^** | - 1. Y, 1.2 Y, 1.3 N   Low RoB | **-** | **-** | 4.1 N, 4.2 N; 4.6 Y  Low RoB | 5.1 Y  Low RoB | 6.1 N, 6.2 N, 6.3 NI  NI about RoB  (Probably moderate) | 7.1 NI, 7.2 N, 7.3 N  NI about RoB  (Probably moderate) | Moderate |
| **Zhang 2018^[35]^** | 1.1 Y; 1.2 N; 1.4 PY; 1.5 NI; 1.6 N  Moderate RoB  (Age, gender, AFP level, tumor size, tumor number, Child-Pugh class didn’t differ between groups, no data about HBV infected) | 2.1 N; 2.4 Y  Low RoB | 3.1 Y; 3.2 Y;  3.3 NI  NI about RoB | 4.1 N  Low RoB | 5.1 Y; 5.2 NI; 5.3 PY; 5.4 NI; 5.5 NI  NI about RoB  (Probably moderate) | 6.1 N; 6.2 NI; 6.3 Y; 6.4 N  Low RoB | 7.1 N; 7.2 N; 7.3 N  Low RoB | Moderate |
| **Ho 2009^[36]^** | 1.1 Y; 1.2 N; 1.4 PY; 1.5 NI; 1.6 N  High RoB  (No relative data) | 2.1 N; 2.4 Y  Low RoB | 3.1 Y; 3.2 Y;  3.3 NI  NI about RoB | 4.1 N  Low RoB | 5.1 Y; 5.2 NI; 5.3 PY; 5.4 NI; 5.5 NI  NI about RoB  (Probably moderate) | 6.1 N; 6.2 NI; 6.3 Y; 6.4 N  Low RoB | 7.1 N; 7.2 N; 7.3 N  Low RoB | High |

**References**

1. Zhou C, Peng Y, Zhou K, Zhang L, Zhang X, Yu L*, et al.* Surgical resection plus radiofrequency ablation for the treatment of multifocal hepatocellular carcinoma. **Hepatobiliary Surg Nutr** **2019**, 8(1)**:** 19-28.

2. Lencioni R, Llovet JM, Han G, Tak WY, Yang J, Guglielmi A*, et al.* Sorafenib or placebo plus TACE with doxorubicin-eluting beads for intermediate stage HCC: The SPACE trial. **J Hepatol** **2016**, 64(5)**:** 1090-1098.

3. Kim GH, Kim JH, Ko HK, Chu HH, Kim SH, Shin JH*, et al.* Surgical Resection plus Intraoperative Radiofrequency Ablation versus Chemoembolization for the Treatment of Intermediate-Stage (BCLC B) Hepatocellular Carcinoma with Preserved Liver Function: A Propensity Score-Matched Analysis. **Cancers (Basel)** **2022**, 14(10).

4. Endo K, Kuroda H, Oikawa T, Okada Y, Fujiwara Y, Abe T*, et al.* Efficacy of combination therapy with transcatheter arterial chemoembolization and radiofrequency ablation for intermediate-stage hepatocellular carcinoma. **Scand J Gastroenterol** **2018**, 53(12)**:** 1575-1583.

5. Lin CW, Chen YS, Lo GH, Hsu YC, Hsu CC, Wu TC*, et al.* Comparison of overall survival on surgical resection versus transarterial chemoembolization with or without radiofrequency ablation in intermediate stage hepatocellular carcinoma: a propensity score matching analysis. **BMC Gastroenterol** **2020**, 20(1)**:** 99.

6. Hirooka M, Hiraoka A, Ochi H, Kisaka Y, Joko K, Michitaka K*, et al.* Transcatheter Arterial Chemoembolization With or Without Radiofrequency Ablation: Outcomes in Patients With Barcelona Clinic Liver Cancer Stage B Hepatocellular Carcinoma. **AJR Am J Roentgenol** **2018**, 210(4)**:** 891-898.

7. Nouso K, Kariyama K, Nakamura S, Oonishi A, Wakuta A, Oyama A*, et al.* Application of radiofrequency ablation for the treatment of intermediate-stage hepatocellular carcinoma. **J Gastroenterol Hepatol** **2017**, 32(3)**:** 695-700.

8. Peng Y, Liu F, Xu H, Wei Y, Li B. Is laparoscopic liver resection suitable for selected patients with BCLC stage B HCC? A propensity score-matched analysis. **HPB (Oxford)** **2020**, 22(4)**:** 595-602.

9. Ke Y, Zhong J, Guo Z, Liang Y, Li L, Xiang B. [Comparison liver resection with transarterial chemoembolization for Barcelona Clinic Liver Cancer stage B hepatocellular carcinoma patients on long-term survival after SPSS propensity score matching]. **Zhonghua Yi Xue Za Zhi** **2014**, 94(10)**:** 747-750.

10. Chen L, Kan X, Sun T, Ren Y, Cao Y, Yan L*, et al.* Transarterial chemoembolization combined with iodine 125 seeds versus transarterial chemoembolization combined with radiofrequency ablation in the treatment of early- and intermediate-stage hepatocellular carcinoma. **BMC Gastroenterol** **2020**, 20(1)**:** 205.

11. Tada T, Kumada T, Toyoda H, Tsuji K, Hiraoka A, Itobayashi E*, et al.* Role of hepatic resection in patients with intermediate-stage hepatocellular carcinoma: A multicenter study from Japan. **Cancer Sci** **2017**, 108(7)**:** 1414-1420.

12. Espinosa W, Liu YW, Wang CC, Lin CC, Wang JH, Lu SN*, et al.* Combined resection and radiofrequency ablation versus transarterial embolization for intermediate-stage hepatocellular carcinoma: A propensity score matching study. **J Formos Med Assoc** **2018**, 117(3)**:** 197-203.

13. Hou YF, Wei YG, Yang JY, Wen TF, Xu MQ, Yan LN*, et al.* Combined hepatectomy and radiofrequency ablation versus TACE in improving survival of patients with unresectable BCLC stage B HCC. **Hepatobiliary Pancreat Dis Int** **2016**, 15(4)**:** 378-385.

14. Akarapatima K, Chang A, Prateepchaiboon T, Pungpipattrakul N, Songjamrat A, Pakdeejit S*, et al.* Comparison of Overall Survival between Transarterial Chemoembolization and Best Supportive Care in Intermediate- Stage Hepatocellular Carcinoma. **Asian Pac J Cancer Prev** **2022**, 23(9)**:** 3173-3178.

15. Kariyama K, Nouso K, Wakuta A, Oonishi A, Toyoda H, Tada T*, et al.* Treatment of Intermediate-Stage Hepatocellular Carcinoma in Japan: Position of Curative Therapies. **Liver Cancer** **2020**, 9(1)**:** 41-49.

16. Kim JY, Sinn DH, Gwak GY, Choi GS, Saleh AM, Joh JW*, et al.* Transarterial chemoembolization versus resection for intermediate-stage (BCLC B) hepatocellular carcinoma. **Clin Mol Hepatol** **2016**, 22(2)**:** 250-258.

17. Ohama H, Hiraoka A, Tada F, Kato K, Fukunishi Y, Yanagihara E*, et al.* Clinical Usefulness of Surgical Resection Including the Complementary Use of Radiofrequency Ablation for Intermediate-Stage Hepatocellular Carcinoma. **Cancers (Basel)** **2022**, 15(1).

18. Zhou Q, Tuo F, Li R, Wang X, Wang J, Huang Z*, et al.* Transarterial Chemoembolization Combined With Hepatectomy for the Treatment of Intermediate-Stage Hepatocellular Carcinoma. **Front Oncol** **2020**, 10**:** 578763.

19. Yang B, Yuan M, Yang T, Liao Z, Wu H. Transarterial chemoembolization vs. liver resection as initial treatment for hepatocellular carcinoma occurring exclusively in caudate lobe: A retrospective propensity matching analysis. **Oncol Res** **2022**, 30(1)**:** 23-33.

20. Wang XH, Zhou QF, Wang CM, Xiang CL, Song YH, Li SQ*, et al.* Adjuvant transarterial chemoembolization for intermediate-stage hepatocellular carcinoma with microvascular invasion. **Br J Surg** **2023**, 110(8)**:** 913-916.

21. Wang L, Lin N, Lin K, Xiao C, Wang R, Chen J*, et al.* The Clinical Value of Postoperative Transarterial Chemoembolization for Resectable Patients with Intermediate Hepatocellular Carcinoma After Radical Hepatectomy: a Propensity Score-Matching Study. **J Gastrointest Surg** **2021**, 25(5)**:** 1172-1183.

22. Nong X, Zhang Y, Xie J, Liang J, Xie A, Zhang Z. Evaluation of the up-to-7 criterion for determining the treatment of hepatocellular carcinoma in Barcelona Clinic Liver Cancer stage B: a single-center retrospective cohort study. **J Gastrointest Oncol** **2023**, 14(2)**:** 768-779.

23. Hu Z, Wang X, Fu Y, Yang D, Zhou Z, Chen M*, et al.* Survival benefit of liver resection following complete response to transarterial chemoembolization for intermediate-stage hepatocellular carcinoma: a retrospective, multicenter, cohort study. **Int J Surg** **2024**, 110(2)**:** 1019-1027.

24. Zhu HD, Li HL, Huang MS, Yang WZ, Yin GW, Zhong BY*, et al.* Transarterial chemoembolization with PD-(L)1 inhibitors plus molecular targeted therapies for hepatocellular carcinoma (CHANCE001). **Signal Transduct Target Ther** **2023**, 8(1)**:** 58.

25. Huang CT, Chu YL, Su TH, Huang SC, Tseng TC, Hsu SJ*, et al.* Optimizing Survival Benefit by Surgical Resection by the Seven-Eleven Criteria in Barcelona Clinic Liver Cancer Stage A/B Hepatocellular Carcinoma beyond the Milan Criteria. **Liver Cancer** **2023**, 12(6)**:** 539-549.

26. Li H, Wang J, Zhang G, Kuang D, Li Y, He X*, et al.* Transarterial chemoembolization combined donafenib with/without PD-1 for unresectable HCC in a multicenter retrospective study. **Front Immunol** **2023**, 14**:** 1277329.

27. Chen S, Jin H, Dai Z, Wei M, Xiao H, Su T*, et al.* Liver resection versus transarterial chemoembolization for the treatment of intermediate-stage hepatocellular carcinoma. **Cancer Med** **2019**, 8(4)**:** 1530-1539.

28. Yan J, Man Z, Lu Q, Ma K. Long-Term Survival in Patients Receiving Combination Therapy with Resection and Radiofrequency Ablation for Multi-Focal Hepatocellular Carcinoma Classified as Barcelona Clinic Liver Cancer Stage B: A Retrospective Controlled Study. **Cancer Manag Res** **2020**, 12**:** 2613-2621.

29. Kudo M, Cheng AL, Park JW, Park JH, Liang PC, Hidaka H*, et al.* Orantinib versus placebo combined with transcatheter arterial chemoembolisation in patients with unresectable hepatocellular carcinoma (ORIENTAL): a randomised, double-blind, placebo-controlled, multicentre, phase 3 study. **Lancet Gastroenterol Hepatol** **2018**, 3(1)**:** 37-46.

30. Lu L, Zheng P, Wu Z, Chen X. Hepatic Resection Versus Transarterial Chemoembolization for Intermediate-Stage Hepatocellular Carcinoma: A Cohort Study. **Front Oncol** **2021**, 11**:** 618937.

31. Luo J, Peng ZW, Guo RP, Zhang YQ, Li JQ, Chen MS*, et al.* Hepatic resection versus transarterial lipiodol chemoembolization as the initial treatment for large, multiple, and resectable hepatocellular carcinomas: a prospective nonrandomized analysis. **Radiology** **2011**, 259(1)**:** 286-295.

32. Xu W, Rao Q, An Y, Li M, Xu G, Sang X*, et al.* Proposal for subclassification to select patients for hepatectomy with intermediate hepatocellular carcinoma and Child-Pugh A liver function: A double-center study from China. **Medicine (Baltimore)** **2018**, 97(32)**:** e11800.

33. Ciria R, López-Cillero P, Gallardo AB, Cabrera J, Pleguezuelo M, Ayllón MD*, et al.* Optimizing the management of patients with BCLC stage-B hepatocellular carcinoma: Modern surgical resection as a feasible alternative to transarterial chemoemolization. **Eur J Surg Oncol** **2015**, 41(9)**:** 1153-1161.

34. Yin L, Li H, Li AJ, Lau WY, Pan ZY, Lai EC*, et al.* Partial hepatectomy vs. transcatheter arterial chemoembolization for resectable multiple hepatocellular carcinoma beyond Milan Criteria: a RCT. **J Hepatol** **2014**, 61(1)**:** 82-88.

35. Zhang R, Shen L, Zhao L, Guan Z, Chen Q, Li W. Combined transarterial chemoembolization and microwave ablation versus transarterial chemoembolization in BCLC stage B hepatocellular carcinoma. **Diagn Interv Radiol** **2018**, 24(4)**:** 219-224.

36. Ho MC, Huang GT, Tsang YM, Lee PH, Chen DS, Sheu JC*, et al.* Liver resection improves the survival of patients with multiple hepatocellular carcinomas. **Ann Surg Oncol** **2009**, 16(4)**:** 848-855.

**Table s5 Risk of bias in studies reporting on risk of PFS**

| **Study** | **RoB due to confounding (for RCTs this domain assessed the randomization process) (Part 1)** | | **RoB in selection of participants (Part 2) (only for NRS)** | **RoB in classification of interventions (Part 3) (only for NRS)** | **RoB due to deviation from intended interventions (Part 4)** | **RoB due to missing data (Part 5)** | **RoB in measurement of the outcome (Part 6)** | **RoB in selection of reported results (Part 7)** | **Overall RoB** |
| --- | --- | --- | --- | --- | --- | --- | --- | --- | --- |
| **Kim 2022 ^[1]^** | - 1. Y; 1.2 N; 1.4 Y; 1.5 Y; 1.6 PN   Low RoB  (Adjustment for age, gender, etiology (HBV/HCV infected), tumor size, tumor number and AFP using propensity score matching, no control for Child-Pugh class) | | 2.1 N; 2.4 Y  Low RoB | 3.1 Y; 3.2 Y; 3.3 NI  Low RoB | 4.1 N  Low RoB | 5.1 Y; 5.2 N; 5.3 N  Low RoB | 6.1 NI; 6.2 NI; 6.3 Y; 6.4 N  NI about RoB  (NI abut method of ascertainment of outcome) | 7.1 N; 7.2 N; 7.3 N  Low RoB | Low |
| **Kudo 2020 ^[2]^** | - 1. NI, 1.2 NI, 1.3 N   Some concerns  (Method of randomization not reported) | | **-** | **-** | 4.1 N, 4.2 N, 4.6 Y  Low RoB | 5.1 Y  Low RoB | 6.1 N, 6.2 N, 6.3 N  Low RoB | 7.1 NI, 7.2 N, 7.3 N  Some concerns | Moderate |
| **Zhu 2023 ^[3]^** | 1.1 Y; 1.2 N; 1.4 PY; 1.5 NI; 1.6 N  low RoB  (Age, gender, AFP level, tumor size, tumor number, HBV infection, Child-Pugh class didn’t differ between groups) | | 2.1 N; 2.4 Y  Low RoB | 3.1 Y; 3.2 Y;  3.3 NI  NI about RoB | 4.1 N  Low RoB | 5.1 Y; 5.2 NI; 5.3 NI  NI about RoB  (Probably low) | 6.1 N; 6.2 NI; 6.3 Y; 6.4 N  Low RoB | 7.1 N; 7.2 N; 7.3 N  Low RoB | Low |
| **Lencioni 2016 ^[4]^** | - 1. NI, 1.2 NI, 1.3 N   Some concerns  (Method of randomization not reported) | | **-** | **-** | 4.1 N, 4.2 N, 4.6 Y  Low RoB | 5.1 Y  Low RoB | 6.1 N, 6.2 N, 6.3 N  Low RoB | 7.1 NI, 7.2 N, 7.3 N  Some concerns | Moderate |
| **Espinosa 2018 ^[5]^** | 1.1 Y; 1.2 N; 1.4 PY; 1.5 Y; 1.6 N  Low RoB  (a propensity-matched analysis was performed to adjust for age, gender, etiology (HBV/HCV infected), AFP, child-pugh class, tumor number and tumor size) | | 2.1 N; 2.4 Y  Low RoB | 3.1 Y; 3.2 Y;  3.3 N  Low RoB | 4.1 N  Low RoB | 5.1 Y; 5.2 NI; 5.3 NI  NI about RoB | 6.1 N; 6.2 NI; 6.3 Y; 6.4 N  Low RoB | 7.1 N; 7.2 N; 7.3 N  Low RoB | Low |
| **Li 2023 ^[6]^** | 1.1 Y; 1.2 N; 1.4 PY; 1.5 NI; 1.6 N  low RoB  (Age, gender, AFP level, tumor size, tumor number, HBV infection, Child-Pugh class didn’t differ between groups) | | 2.1 N; 2.4 Y  Low RoB | 3.1 Y; 3.2 Y;  3.3 N  Low RoB | 4.1 N  Low RoB | 5.1 Y; 5.2 NI; 5.3 N  NI about RoB | 6.1 N; 6.2 NI; 6.3 Y; 6.4 N  Low RoB | 7.1 N; 7.2 N; 7.3 N  Low RoB | Low |
| **Lencioni 2024 ^[7]^** | - 1. NI, 1.2 NI, 1.3 N   Some concerns  (Method of randomization not reported) | | **-** | **-** | 4.1 N, 4.2 N, 4.6 Y  Low RoB | 5.1 Y  Low RoB | 6.1 N, 6.2 N, 6.3 N  Low RoB | 7.1 NI, 7.2 N, 7.3 N  Some concerns | Moderate |
| **Zhang 2018^[8]^** | 1.1 Y; 1.2 N; 1.4 PY; 1.5 NI; 1.6 N  Moderate RoB  (Age, gender, AFP level, tumor size, tumor number, Child-Pugh class didn’t differ between groups, no data about HBV infected) | | 2.1 N; 2.4 Y  Low RoB | 3.1 Y; 3.2 Y;  3.3 NI  NI about RoB | 4.1 N  Low RoB | 5.1 Y; 5.2 NI; 5.3 PY; 5.4 NI; 5.5 NI  NI about RoB  (Probably moderate) | 6.1 N; 6.2 NI; 6.3 Y; 6.4 N  Low RoB | 7.1 N; 7.2 N; 7.3 N  Low RoB | Moderate |
| **Nong 2023 ^[9]^** | 1.1 Y; 1.2 N; 1.4 PY; 1.5 NI; 1.6 N  Moderate RoB  (Age, gender, AFP level, HBV infection didn’t differ between groups; tumor number significantly differs between the two groups, no control for tumor size, Child-Pugh class) | 2.1 N; 2.4 Y  Low RoB | | 3.1 Y; 3.2 Y;  3.3 N  Low RoB | 4.1 N  Low RoB | 5.1 Y; 5.2 NI; 5.3 N  NI about RoB (Moderate) | 6.1 N; 6.2 NI; 6.3 Y; 6.4 N  Low RoB | 7.1 N; 7.2 N; 7.3 N  Low RoB | Moderate |

**References**

1. Kim GH, Kim JH, Ko HK, Chu HH, Kim SH, Shin JH*, et al.* Surgical Resection plus Intraoperative Radiofrequency Ablation versus Chemoembolization for the Treatment of Intermediate-Stage (BCLC B) Hepatocellular Carcinoma with Preserved Liver Function: A Propensity Score-Matched Analysis. **Cancers (Basel)** **2022**, 14(10).

2. Kudo M, Ueshima K, Ikeda M, Torimura T, Tanabe N, Aikata H*, et al.* Randomised, multicentre prospective trial of transarterial chemoembolisation (TACE) plus sorafenib as compared with TACE alone in patients with hepatocellular carcinoma: TACTICS trial. **Gut** **2020**, 69(8)**:** 1492-1501.

3. Zhu HD, Li HL, Huang MS, Yang WZ, Yin GW, Zhong BY*, et al.* Transarterial chemoembolization with PD-(L)1 inhibitors plus molecular targeted therapies for hepatocellular carcinoma (CHANCE001). **Signal Transduct Target Ther** **2023**, 8(1)**:** 58.

4. Lencioni R, Llovet JM, Han G, Tak WY, Yang J, Guglielmi A*, et al.* Sorafenib or placebo plus TACE with doxorubicin-eluting beads for intermediate stage HCC: The SPACE trial. **J Hepatol** **2016**, 64(5)**:** 1090-1098.

5. Espinosa W, Liu YW, Wang CC, Lin CC, Wang JH, Lu SN*, et al.* Combined resection and radiofrequency ablation versus transarterial embolization for intermediate-stage hepatocellular carcinoma: A propensity score matching study. **J Formos Med Assoc** **2018**, 117(3)**:** 197-203.

6. Li H, Wang J, Zhang G, Kuang D, Li Y, He X*, et al.* Transarterial chemoembolization combined donafenib with/without PD-1 for unresectable HCC in a multicenter retrospective study. **Front Immunol** **2023**, 14**:** 1277329.

7. Riccardo Lencioni, Masatoshi Kudo, Joseph Erinjeri, Shukui Qin, Zhenggang Ren, Stephen L chan*, et al.* EMERALD-1: A phase 3, randomized, placebo-controlled study of transarterial chemoembolization combined with durvalumab with or without bevacizumab in participants with unresectable hepatocellular carcinoma eligible for embolization. *ASCO-GI LBA432*; 2024.

8. Zhang R, Shen L, Zhao L, Guan Z, Chen Q, Li W. Combined transarterial chemoembolization and microwave ablation versus transarterial chemoembolization in BCLC stage B hepatocellular carcinoma. **Diagn Interv Radiol** **2018**, 24(4)**:** 219-224.

9. Nong X, Zhang Y, Xie J, Liang J, Xie A, Zhang Z. Evaluation of the up-to-7 criterion for determining the treatment of hepatocellular carcinoma in Barcelona Clinic Liver Cancer stage B: a single-center retrospective cohort study. **J Gastrointest Oncol** **2023**, 14(2)**:** 768-779.

**Table S6 Standard meta-analysis of OS**

Firstly, a pairwise meta-analysis was performed; the treatment method before “-” was selected as the first treatment, while the treatment therapy after “-” indicated as the second treatment; the HRs <1 favors the first treatment, however, the HRs >1 favors the second treatment.

| **Comparison** | **N studies** | **N1** | **N2** | **N (Total)** | **HR (95%CI)** | **P-value**  **(for HR)** | **I^2^ (P-value)** | **Model** |
| --- | --- | --- | --- | --- | --- | --- | --- | --- |
| TACE+TKIs-TACE | 2 | 363 | 382 | 745 | 0.86 (0.68-1.09) | 0.22 | 0 (0.78) | fixed |
| Liver resection+RFA-TACE | 5 | 261 | 610 | 871 | 0.52 (0.41-0.66) | <0.001 | 59.5 (0.04) | random |
| TACE+RFA-TACE | 4 | 165 | 340 | 505 | 0.57 (0.42-0.78) | <0.001 | 0 (0.55) | fixed |
| Liver resection-TACE | 15 | 2029 | 2360 | 4389 | 0.53 (0.44-0.62) | <0.001 | 67.6 (<0.001) | random |
| liver resection+TACE -liver resection | 2 | 228 | 228 | 456 | 0.71 (0.55-0.93) | 0.01 | 0 (0.58) | fixed |
| TACE-BSC | 2 | 255 | 96 | 351 | 0.39 (0.25-0.60) | <0.001 | 54.7 (0.06) | random |
| RFA-TACE | 1 | 89 | 89 | 178 | 0.7 (0.44-1.12) | NA | NA | NA |
| TACE+I-125-TACE+RFA | 1 | 38 | 74 | 112 | 0.83 (0.42-1.65) | NA | NA | NA |
| liver resection+RFA-liver resection | 1 | 25 | 45 | 70 | 0.92 (0.38-2.25) | NA | NA | NA |
| liver resection-TACE+liver resection | 1 | 265 | 223 | 488 | 1.94 (1.52-2.48) | NA | NA | NA |
| TACE+ICIs+TKIs-TACE+TKIs | 1 | 39 | 28 | 67 | 0.18 (0.08-0.42) | NA | NA | NA |
| TACE+liver resection-TACE | 1 | 98 | 98 | 196 | 0.53 (0.32-0.89) | NA | NA | NA |
| TACE+ICIs+TKIs-TACE | 1 | 78 | 77 | 165 | 0.84 (0.51-1.40) | NA | NA | NA |

**Figure S1 Risk-of-bias contribution chart (percent stacked bar chart) to assess the credibility of evidence using CINeMA tool in terms of OS**


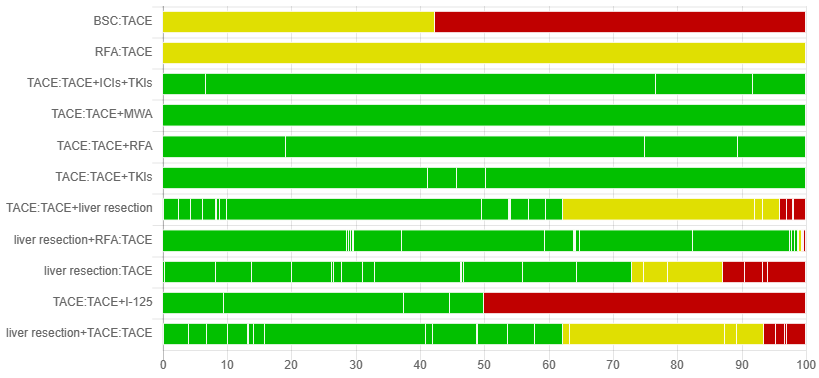


Percentage of studies at low RoB (green color), moderate RoB (yellow color), and high RoB (red color) in each pairwise comparison

**Table S7: Assessing the credibility of evidence using the CINeMA tool in terms of OS**

| **Comparison** | **Number of studies** | **Within-study bias** | **Reporting bias** | **Indirectness** | **Imprecision** | **Heterogeneity** | **Incoherence** | **Confidence rating** |
| --- | --- | --- | --- | --- | --- | --- | --- | --- |
| BSC:TACE | 2 | Major concerns | Low risk | No concerns | No concerns | No concerns | Major concerns | Very low |
| RFA:TACE | 1 | Some concerns | Low risk | No concerns | Major concerns | No concerns | Major concerns | Very low |
| TACE:TACE+ICIs+TKIs | 1 | No concerns | Low risk | No concerns | No concerns | Major concerns | Major concerns | Very low |
| TACE:TACE+MWA | 1 | No concerns | Low risk | No concerns | No concerns | No concerns | Major concerns | Low |
| TACE:TACE+RFA | 4 | No concerns | Low risk | No concerns | No concerns | Major concerns | Major concerns | Very low |
| TACE:TACE+TKIs | 2 | No concerns | Low risk | No concerns | Major concerns | No concerns | Major concerns | Very low |
| TACE:TACE+LR | 1 | No concerns | Low risk | No concerns | No concerns | No concerns | No concerns | High |
| LR:TACE | 15 | No concerns | High risk | No concerns | No concerns | No concerns | No concerns | Low |
| LR+RFA:TACE | 5 | No concerns | Low risk | No concerns | No concerns | No concerns | No concerns | High |
| TACE:TACE+I-125 | 0 | Some concerns | Low risk | No concerns | Major concerns | No concerns | Major concerns | Very low |
| LR+TACE:TACE | 0 | No concerns | Low risk | No concerns | No concerns | No concerns | Major concerns | Low |

**Table s8 Standard meta-analysis of PFS**

Firstly, a pairwise meta-analysis was performed, the treatment method before “-” was selected as first treatment, while the treatment therapy after “-” indicated as second treatment; the HRs <1 favors the first treatment, however, the HRs >1 favors the second treatment.

| **Comparison** | **N studies** | **N1** | **N2** | **N (Total)** | **HR (95%CI)** | **P-value**  **(for HR)** | **I^2^ (P-value)** | **Model** |
| --- | --- | --- | --- | --- | --- | --- | --- | --- |
| TACE+TKIs-TACE | 2 | 198 | 187 | 385 | 0.63 (0.36-1.10) | 0.10 | 69.1 (0.07) | random |
| Liver resection+RFA-TACE | 2 | 121 | 330 | 451 | 0.53 (0.26-1.06) | 0.07 | 77.4 (0.04) | random |
| Liver resection-TACE | 1 | 108 | 55 | 163 | 0.81 (0.44-1.48) | NA | NA | NA |
| TACE+ICIs+TKIs-TACE | 1 | 103 | 226 | 329 | 0.85 (0.57-1.27) | NA | NA | NA |
| TACE+ICIs+bevacizumab-TACE | 1 | 117 | 114 | 231 | 0.71 (0.52-0.95) | NA | NA | NA |
| TACE+ICIs+TKIs -TACE+TKIs | 1 | 39 | 28 | 67 | 0.4 (0.22-0.71) | NA | NA | NA |
| TACE+MWA-TACE | 1 | 50 | 100 | 150 | 0.66 (0.43-1.0) | NA | NA | NA |

**Figure S2 Risk-of-bias contribution chart (percent stacked bar chart) to assess the credibility of evidence using CINeMA tool in terms of PFS**


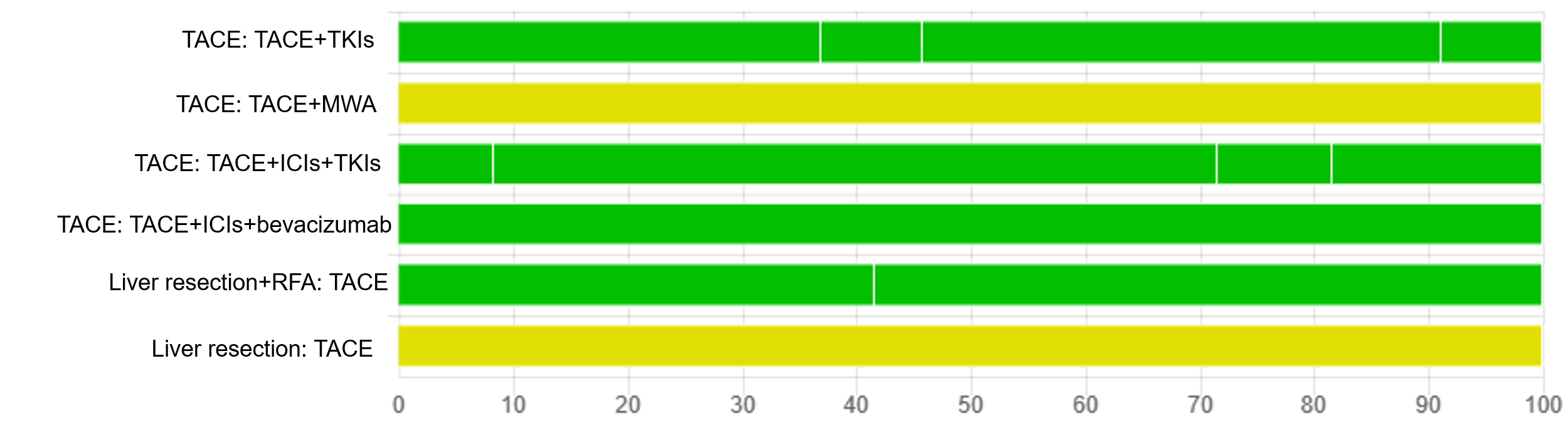


Percentage of studies at low RoB (green color), moderate RoB (yellow color), and high RoB (red color) in each pairwise comparison

**Table s9 Assessing the credibility of evidence using the CINeMA tool**

| **Comparison** | **No. of studies** | **Within-study bias** | **Reporting bias** | **Indirectness** | **Imprecision** | **Heterogeneity** | **Incoherence** | **Confidence rating** |
| --- | --- | --- | --- | --- | --- | --- | --- | --- |
| liver resection+RFA:TACE | 2 | No concerns | Low risk | No concerns | Major concerns | No concerns | Some concerns | **Very low** |
| TACE:TACE+TKIs | 2 | No concerns | Low risk | No concerns | Major concerns | No concerns | No concerns | **Low** |
| TACE:TACE+MWA | 1 | Some concerns | Low risk | No concerns | Major concerns | No concerns | Some concerns | **Very low** |
| TACE:TACE+ICIs +TKIs | 1 | No concerns | Low risk | No concerns | Major concerns | No concerns | No concerns | **Low** |
| TACE:TACE+ICIs+  bevacizumab | 1 | No concerns | Low risk | No concerns | Major concerns | No concerns | Some concerns | **Very low** |
| liver resection:TACE | 1 | Some concerns | Low risk | No concerns | Major concerns | No concerns | Some concerns | **Very low** |
| liver resection+RFA:TACE | 2 | No concerns | Low risk | No concerns | Major concerns | No concerns | Some concerns | **Very low** |

**Subgroup Analysis of Overall Survival (OS)**

In this section, we present the figures of the distribution of all effect modifiers (Publication year, AFP, gender, AFP level, Child-Pugh class, tumor size, tumor number, HCV-infected state, HBV-infected state, sample size) across treatment comparisons. After each figure, we show the subgroup analyses for this effect modifier.

Subgroup analyses are presented in league tables, where each box represents the comparison of the row-defining treatment vs the column-defining treatment. HR is reported first, followed by 95% CI; HRs >1 favor the column-defining treatment, while HRs <1 favor the row-defining treatment. After the league tables, we present the results of all subgroup/sensitivity analyses in a summary table.

**Region, Publication year,**

A: TACE+RFA-TACE; B: Liver Resection-TACE; C: TACE+I-125-TACE+RFA; D: liver resection+RFA-TACE; E: TACE-BSC; F: RFA-TACE; G: liver resection+RFA-liver resection; H: liver resection-TACE+liver resection; I: liver resection-liver resection+TACE; J: TACE+liver resection-TACE; K: TACE+ICIs+TKIs-TACE; L: TACE+ICIs+TKIs- TACE+TKIs; M: TACE+TKIs-TACE; N: TACE+MWA-TACE.

**Other**

A: TACE+ICIs+TKIs-TACE; B: liver resection+TACE-liver resection; C: TACE+liver resection-liver resection; D: liver resection-TACE; E: TACE+liver resection-TACE; F: TACE+ICIs+TKIs-TACE+TKIs; G: TACE+TKIs-TACE; H: TACE+RFA-TACE; I: liver resection+RFA-TACE; J: RFA-TACE; K: TACE-BSC; L: TACE+I-125-TACE+RFA; M: liver resection+RFA-liver resection; S: TACE+MWA-TACE.

1. **Region**

**
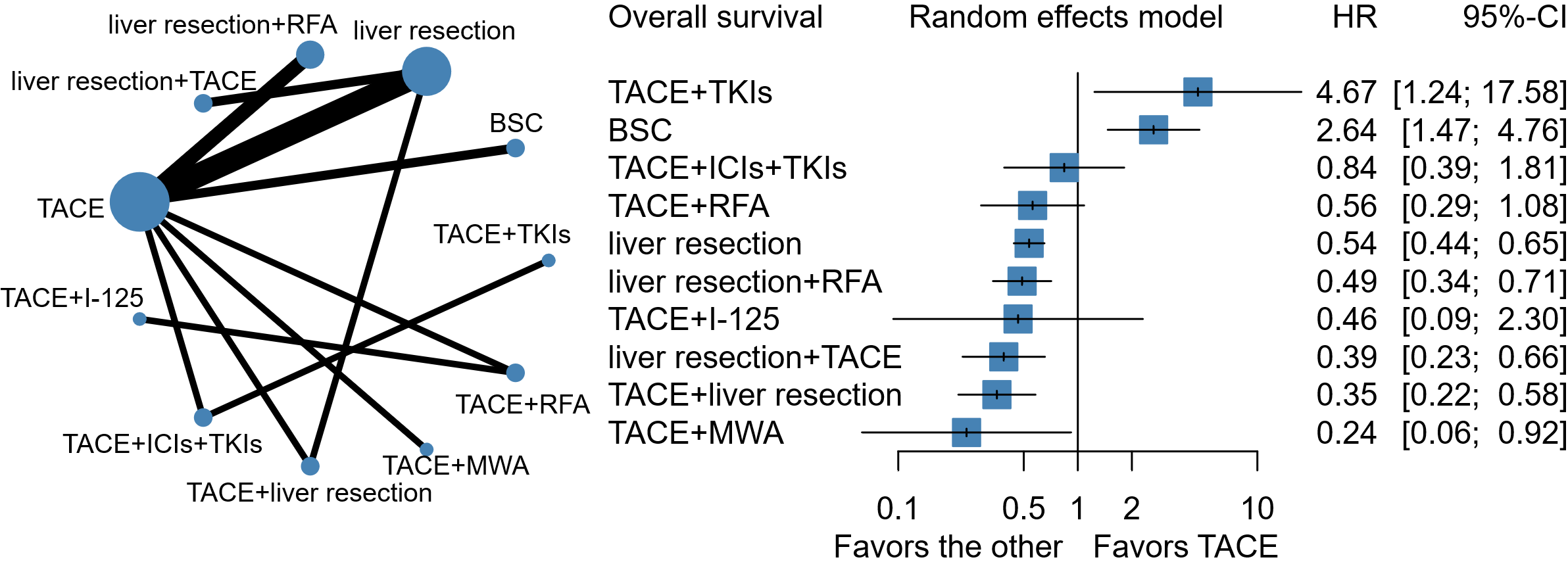
Figure S3 Risk of overall survival in studies in Asia (excluding Japan, n=29 studies)**

**Table s10 Risk of overall survival in studies in Asia (excluding Japan, n= 29 studies)**

| **BSC** | NA | NA | NA | 2.64  (1.47-4.76) | NA | NA | NA | NA | NA | NA |
| --- | --- | --- | --- | --- | --- | --- | --- | --- | --- | --- |
| 4.93  (2.65-9.17) | **LR** | NA | 1.38  (0.85-2.26) | 0.52  (0.43-0.64) | NA | NA | 1.94  (1.05-3.57) | NA | NA | NA |
| 5.40  (2.69-10.83) | 1.09  (0.72-1.67) | **LR+RFA** | NA | 0.49  (0.34-0.71) | NA | NA | NA | NA | NA | NA |
| 6.83  (3.10-15.05) | 1.38  (0.85-2.26) | 1.27  (0.66-2.41) | **LR+TACE** | NA | NA | NA | NA | NA | NA | NA |
| 2.64  (1.47-4.76) | 0.54  (0.44-0.65) | 0.49  (0.34-0.71) | 0.39  (0.23-0.66) | **TACE** | NA | 1.19  (0.55-2.57) | 1.89  (0.87-4.09) | 4.17  (1.09-15.92) | 1.79  (0.92- 3.46) | NA |
| 5.69  (1.03-31.27) | 1.15  (0.23-5.78) | 1.05  (0.20-5.44) | 0.83  (0.15-4.49) | 2.15  (0.43- 10.65) | **TACE+I-125** | NA | NA | NA | 0.83  (0.19- 3.56) | NA |
| 3.15  (1.20-8.28) | 0.64  (0.29-1.41) | 0.58  (0.25-1.37) | 0.46  (0.18-1.17) | 1.19  (0.55-2.57) | 0.55  (0.09-3.27) | **TACE+ICIs**  **+TKIs** | NA | NA | NA | 0.18  (0.06- 0.53) |
| 7.46  (3.46-16.08) | 1.51  (0.93-2.46) | 1.38  (0.74-2.57) | 1.09  (0.55-2.18) | 2.82  (1.72-4.63) | 1.31  (0.25-7.00) | 2.37  (0.95-5.92) | **TACE+LR** | NA | NA | NA |
| 11.01  (2.55-47.59) | 2.23  (0.58-8.65) | 2.04  (0.51-8.20) | 1.61  (0.38-6.81) | 4.17  (1.09-15.92) | 1.94  (0.24-15.61) | 3.50  (0.75-16.41) | 1.48  (0.35-6.16) | **TACE+MWA** | NA | NA |
| 4.72  (1.95-11.42) | 0.96  (0.48-1.90) | 0.87  (0.41-1.87) | 0.69  (0.30-1.61) | 1.79  (0.92-3.46) | 0.83  (0.19-3.56) | 1.50  (0.54-4.13) | 0.63  (0.28-1.44) | 0.43  (0.10-1.91) | **TACE+RFA** | NA |
| 0.57  (0.13-2.42) | 0.11  (0.03-0.44) | 0.10  (0.03-0.42) | 0.08  (0.02-0.35) | 0.21  (0.06-0.81) | 0.10  (0.01-0.80) | 0.18  (0.06-0.53) | 0.08  (0.02-0.31) | 0.05  (0.01-0.34) | 0.12  (0.03-0.53) | **TACE+TKIs** |

**
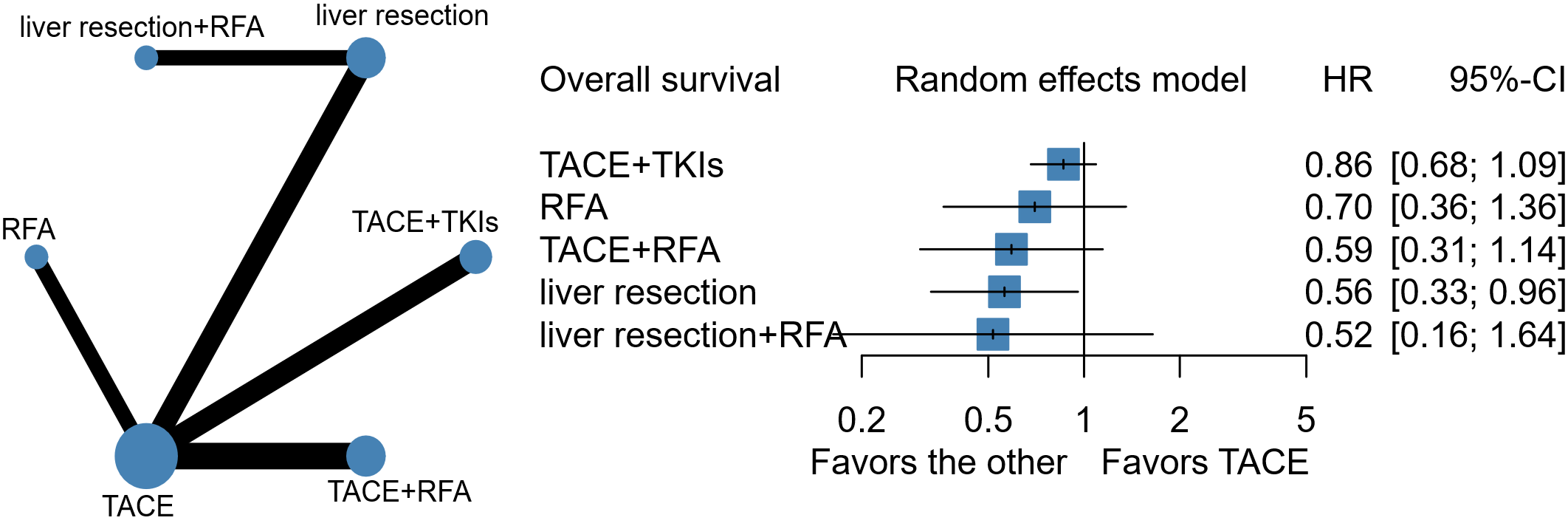
Figure S4 Risk of overall survival in studies in the rest of the region (n=9 studies)**

**Table S11 Risk of overall survival in studies in the rest of the region (n=9 studies)**

| **liver resection** | 1.09 (0.39-3.03) | NA | 0.56  (0.33-0.96) | NA | NA |
| --- | --- | --- | --- | --- | --- |
| 1.09  (0.39-3.03) | **LR+RFA** | NA | NA | NA | NA |
| 0.80  (0.34-1.88) | 0.74 (0.20-2.80) | **RFA** | 0.70  (0.36-1.36) | NA | NA |
| 0.56  (0.33-0.96) | 0.52 (0.16-1.64) | 0.70  (0.36-1.36) | **TACE** | 1.69  (0.87-3.28) | 1.16  (0.92-1.47) |
| 0.95  (0.41-2.22) | 0.87 (0.23-3.31) | 1.18  (0.47-3.01) | 1.69  (0.87-3.28) | **TACE+RFA** | NA |
| 0.65  (0.36-1.17) | 0.60 (0.18-1.95) | 0.81  (0.40-1.64) | 1.16  (0.92-1.47) | 0.69  (0.34-1.38) | **TACE+TKIs** |

**2. Child-Pugh class (the percentage of CT-A class patients)**

**
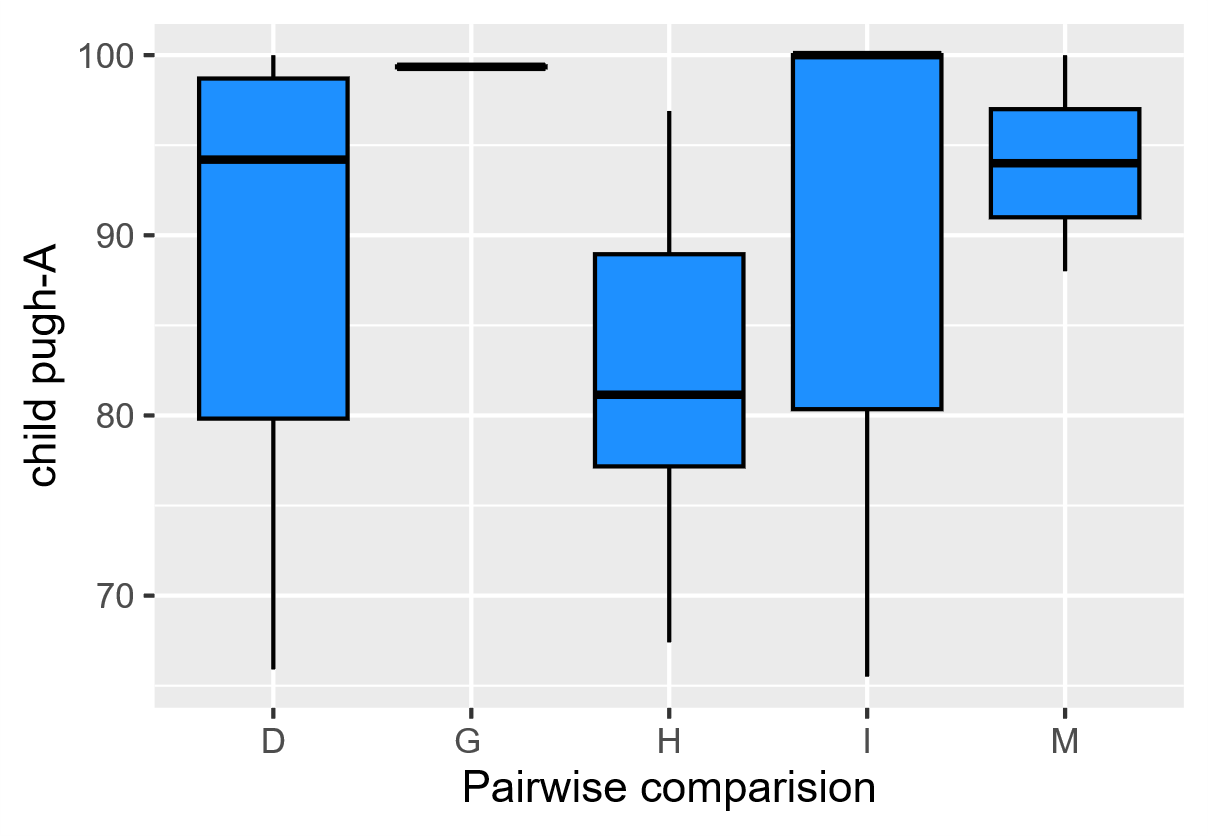
Figure S5: Distribution of Child-Pugh class**

Median of the CT-A class (the percentage of CT-A patients) across studies: 94% (IQR=78-99)

**
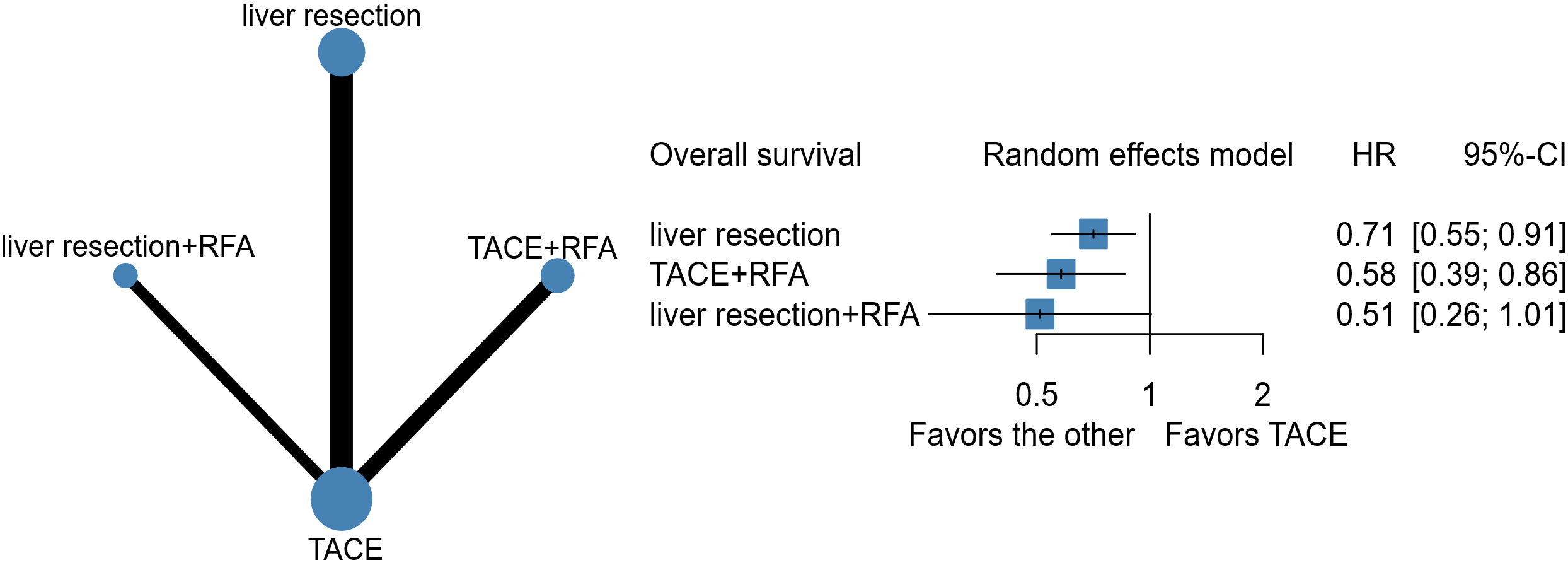
Figure S6 Risk of overall survival in studies with a lower percentage of CT-A class (n=14 studies)**

**Table S12 Risk of overall survival in studies with a lower percentage of CT-A class (n=14 studies)**

| **liver resection** | NA | 0.71 (0.55-0.91) | NA |
| --- | --- | --- | --- |
| 1.39 (0.67-2.87) | **liver resection+RFA** | 0.51 (0.26-1.01) | NA |
| 0.71 (0.55-0.91) | 0.51 (0.26-1.01) | **TACE** | 1.72 (1.16-2.55) |
| 1.22 (0.76-1.95) | 0.88 (0.40-1.93) | 1.72 (1.16-2.55) | **TACE+RFA** |

**
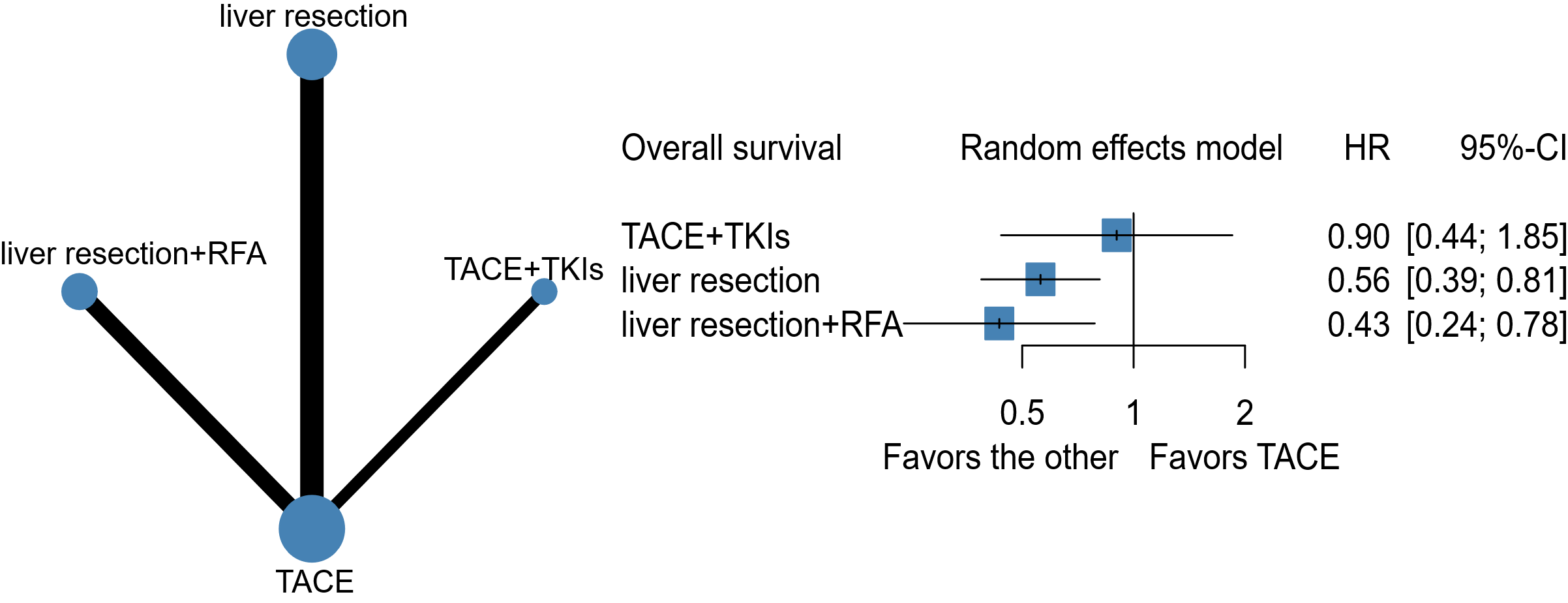
Figure S7 Risk of overall survival in studies with a higher percentage of CT-A class (n=7 studies)**

**Table S13 Risk of overall survival in studies with a higher percentage of CT-A class (n=7 studies)**

| **liver resection** | NA | 0.56 (0.39-0.81) | NA |
| --- | --- | --- | --- |
| 1.29 (0.64-2.60) | **liver resection+RFA** | 0.43 (0.24-0.78) | NA |
| 0.56 (0.39-0.81) | 0.43 (0.24-0.78) | **TACE** | 1.11 (0.54-2.28) |
| 0.62 (0.28-1.40) | 0.48 (0.19-1.22) | 1.11 (0.54-2.28) | **TACE+TKIs** |

3. AFP level (≥400ng/mL)

**
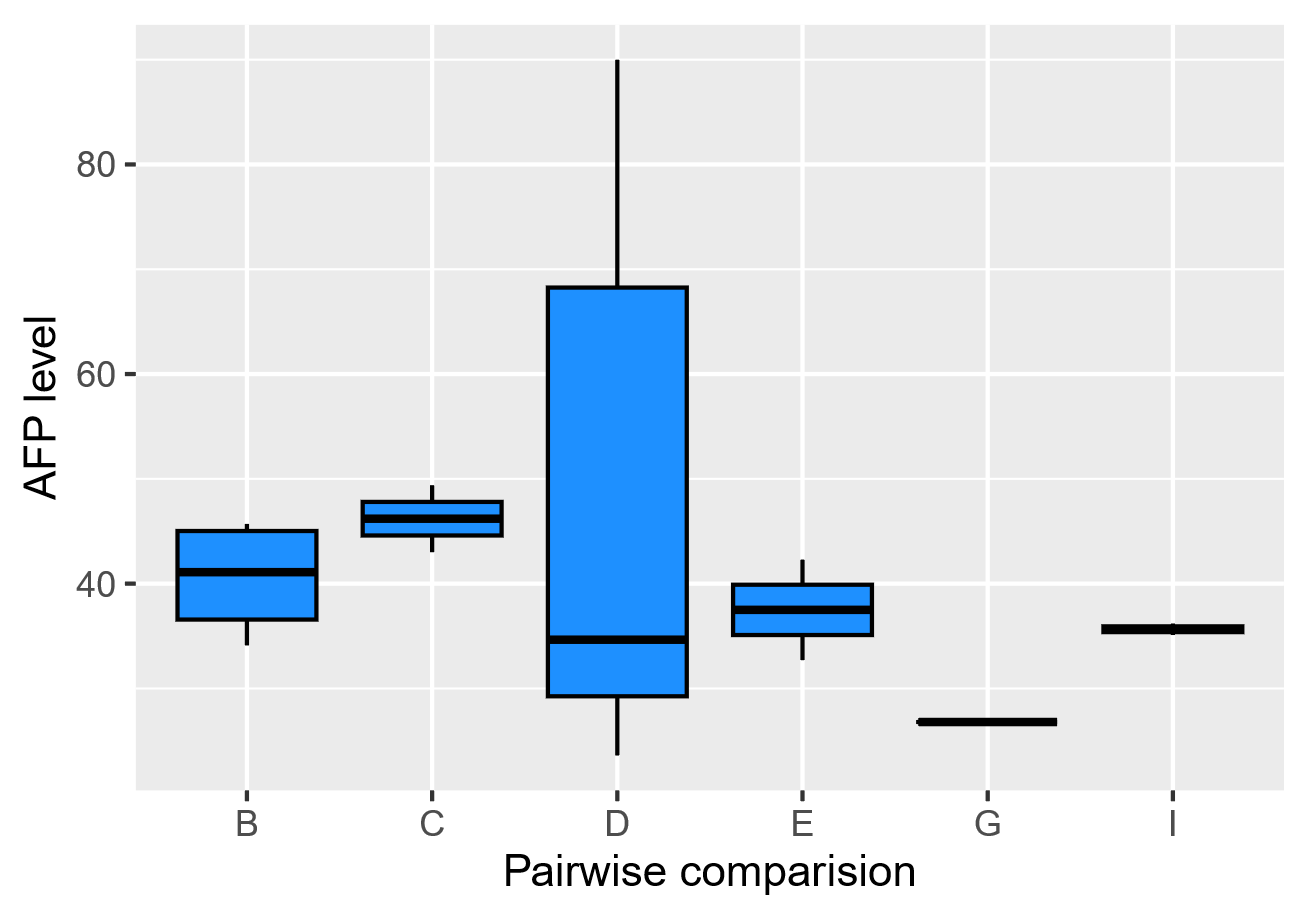
Figure S8: Distribution of AFP level across OS**

Median of the AFP level (the percentage of AFP level ≥400ng/mL) across studies: 37% (IQR=30- 45)

**
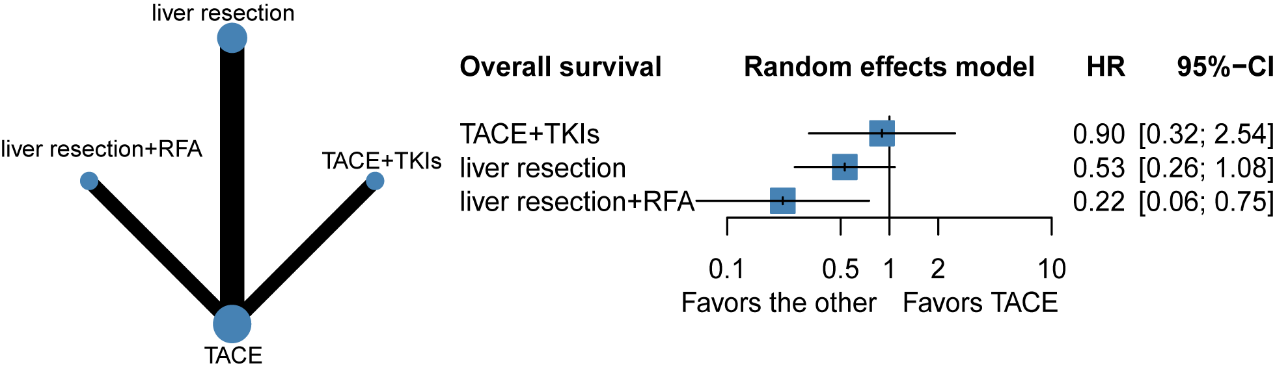
Figure S9 Risk of overall survival in studies with a lower level of AFP (n=5 studies)**

**Table S14 Risk of overall survival in studies with a lower level of AFP (n=5 studies)**

| **liver resection** | NA | 0.53 (0.26- 1.08) | NA |
| --- | --- | --- | --- |
| 2.41 (0.59- 9.93) | **liver resection+RFA** | 0.22 (0.06- 0.75) | NA |
| 0.53 (0.26- 1.08) | 0.22 (0.06- 0.75) | **TACE** | 1.11 (0.39- 3.13) |
| 0.59 (0.17- 2.07) | 0.24 (0.05- 1.22) | 1.11 (0.39- 3.13) | **TACE+TKIs** |

**Figure S10 Risk of overall survival in studies with a higher level of AFP (n=5 studies)**


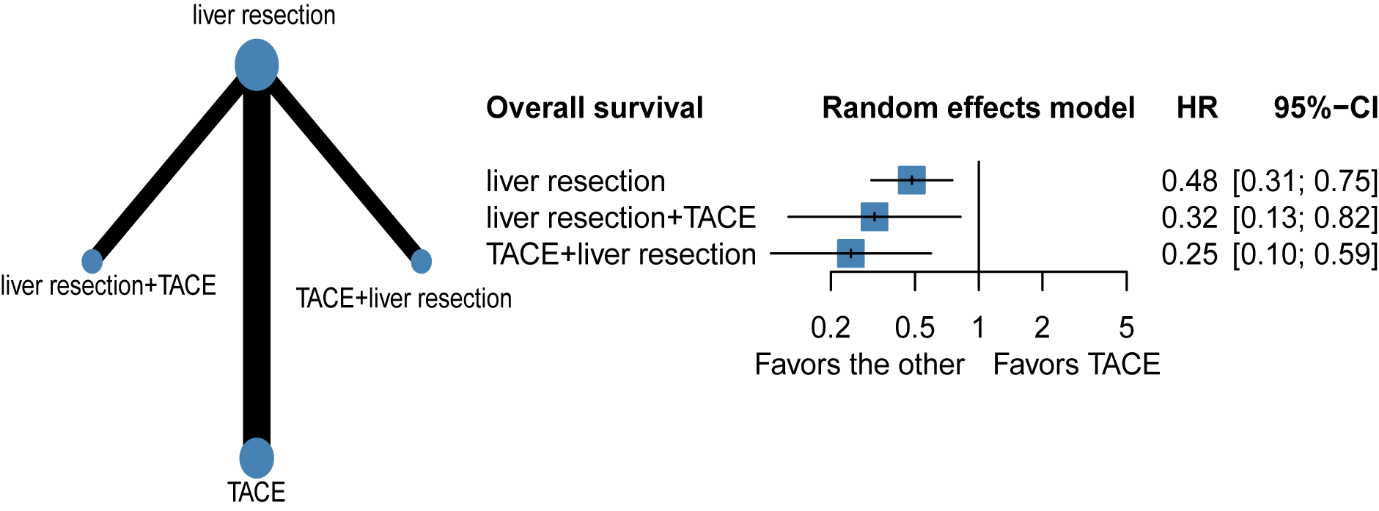


**Table S15 Risk of overall survival in studies with a higher level of AFP (n=5 studies)**

| **liver resection** | 1.50 (0.66-3.43) | 0.48 (0.31-0.75) | 1.94 (0.92-4.11) |
| --- | --- | --- | --- |
| 1.50 (0.66-3.43) | **liver resection+TACE** | NA | NA |
| 0.48 (0.31-0.75) | 0.32 (0.13-0.82) | **TACE** | NA |
| 1.94 (0.92-4.11) | 1.29 (0.42-3.95) | 4.02 (1.68-9.60) | **TACE+liver resection** |

4. HBV-infected (The percentage of HBV-infected patients)

**
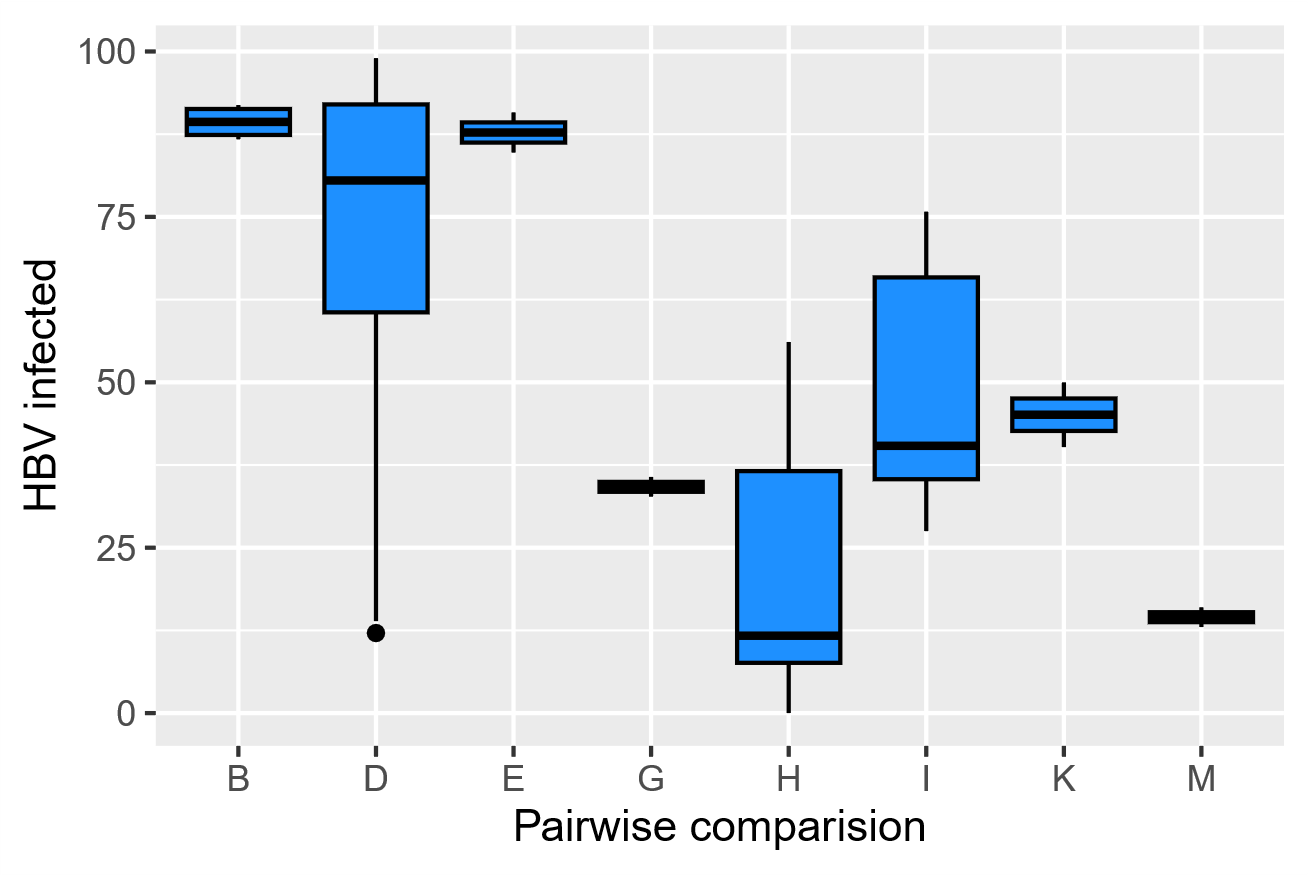
Figure S11: Distribution of the percentage of HBV-infected patients across OS**

Median of the HBV infected (the percentage of HBV-infected patients) across studies: 70% (IQR=34- 88)

**Figure S12 Risk of overall survival in studies with a lower percentage of HBV-infected patients (n=12 studies)**


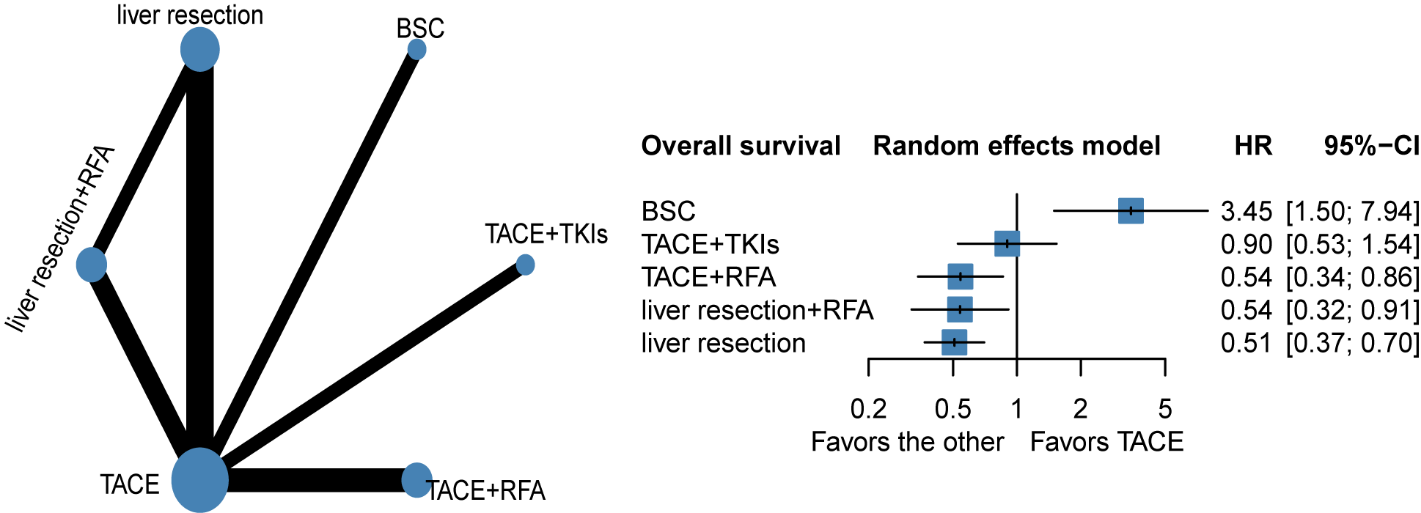


**Table S16 Risk of overall survival in studies with a lower percentage of HBV-infected patients (n=12 studies)**

| **BSC** | NA | NA | 3.45 (1.50-7.94) | NA | NA |
| --- | --- | --- | --- | --- | --- |
| 6.79 (2.78-16.61) | **liver resection** | 1.09 (0.37-3.23) | 0.50 (0.36-0.70) | NA | NA |
| 6.40 (2.39-17.13) | 0.94 (0.53- 1.68) | **liver resection+RFA** | 0.56 (0.31-1.02) | NA | NA |
| 3.45 (1.50- 7.94) | 0.51 (0.37- 0.70) | 0.54 (0.32- 0.91) | **TACE** | 1.84 (1.16-2.92) | 1.11 (0.65-1.90) |
| 6.36 (2.45-16.49) | 0.94 (0.53- 1.64) | 0.99 (0.49- 2.00) | 1.84 (1.16- 2.92) | **TACE+RFA** | NA |
| 3.83 (1.42-10.32) | 0.56 (0.30- 1.05) | 0.60 (0.28- 1.27) | 1.11 (0.65- 1.90) | 0.60 (0.30- 1.22) | **TACE+TKIs** |

**Figure S13 Risk of overall survival in studies with a higher percentage of HBV-infected patients (n=12 studies)**


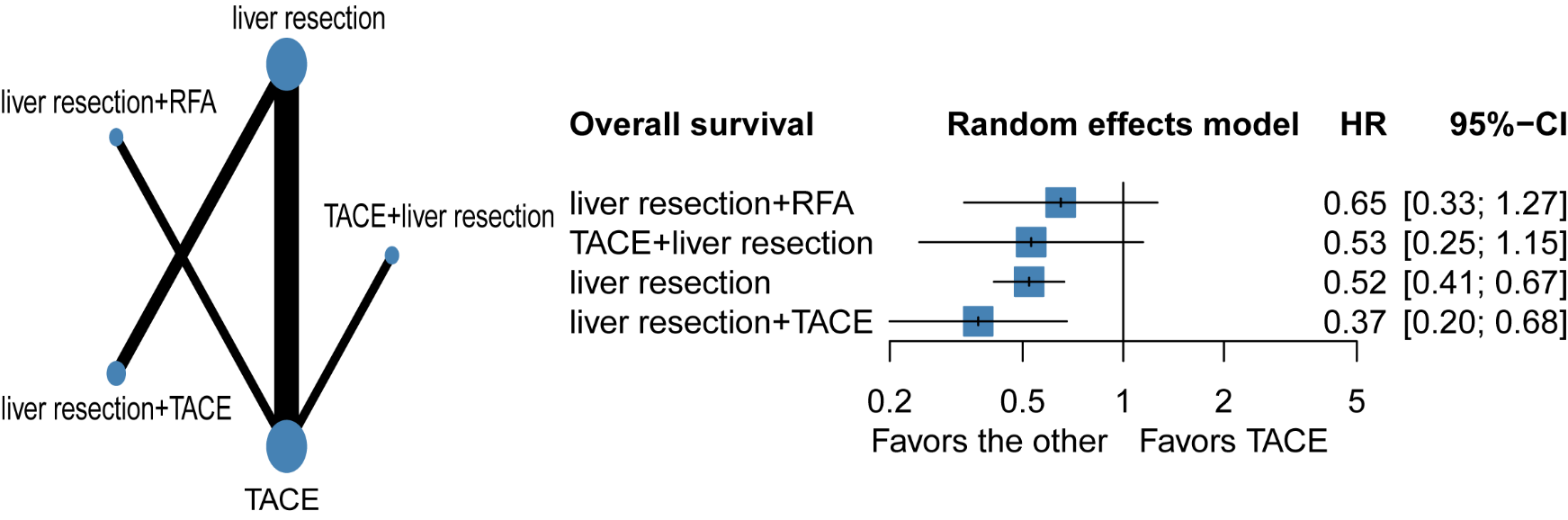


**Table S17 Risk of overall survival in studies with a higher percentage of HBV-infected patients (n=12 studies)**

| **liver resection** | NA | 1.42 (0.81-2.49) | 0.52 (0.41-0.67) | NA |
| --- | --- | --- | --- | --- |
| 0.80 (0.40-1.63) | **liver resection+RFA** | NA | 0.65 (0.33-1.27) | NA |
| 1.42 (0.81-2.49) | 1.77 (0.72-4.36) | **liver resection+TACE** | NA | NA |
| 0.52 (0.41-0.67) | 0.65 (0.33-1.27) | 0.37 (0.20-0.68) | **TACE** | 1.89 (0.87-4.08) |
| 0.99 (0.44-2.21) | 1.23 (0.44-3.40) | 0.69 (0.26-1.86) | 1.89 (0.87-4.08) | **TACE+liver resection** |

5. HCV-infected (The percentage of HCV-infected patients)

**
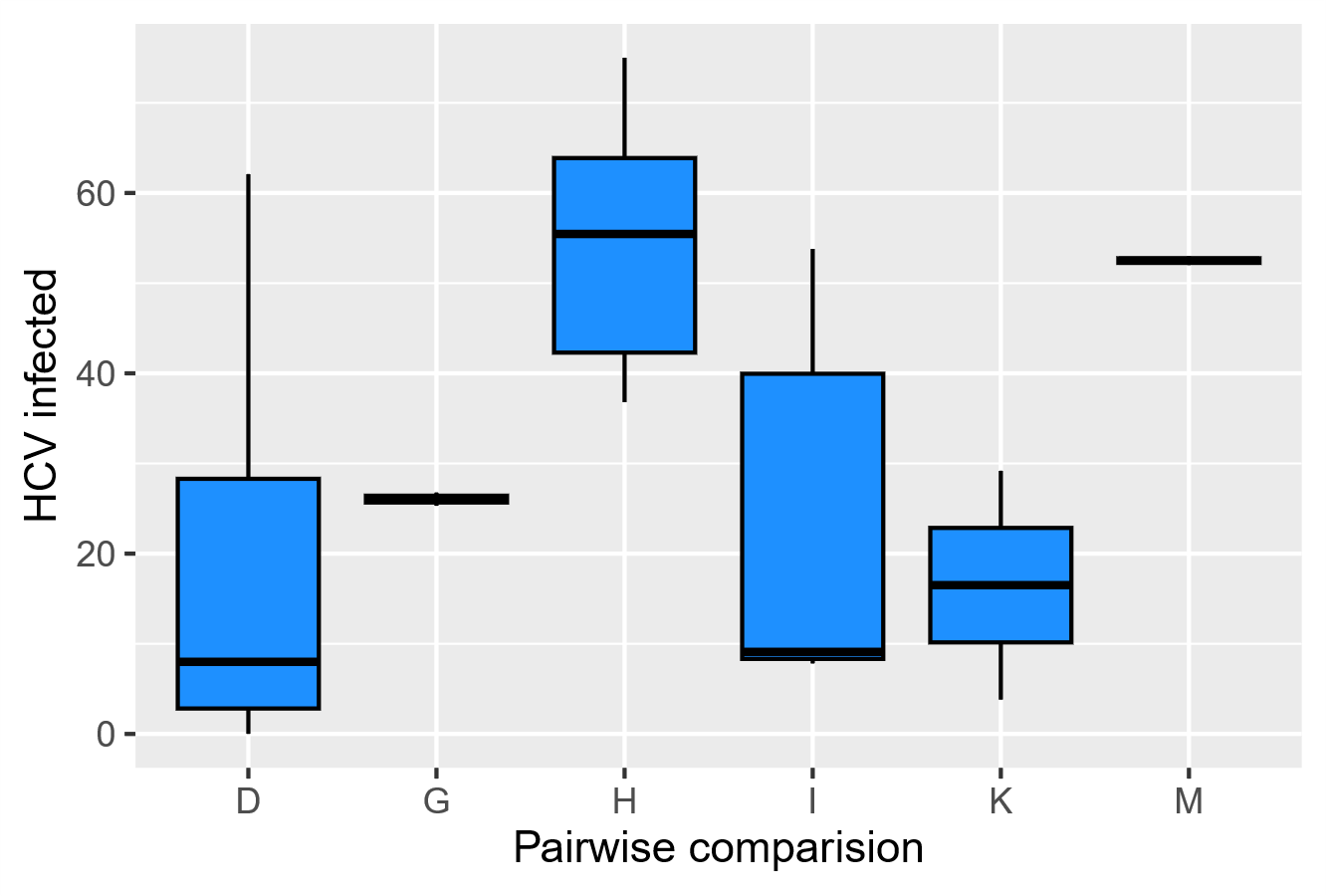
Figure S14 Distribution of the percentage of HCV-infected patients across OS**

Median of the HCV-infected (the percentage of HCV-infected patients) across studies: 26% (IQR=7- 52)

**
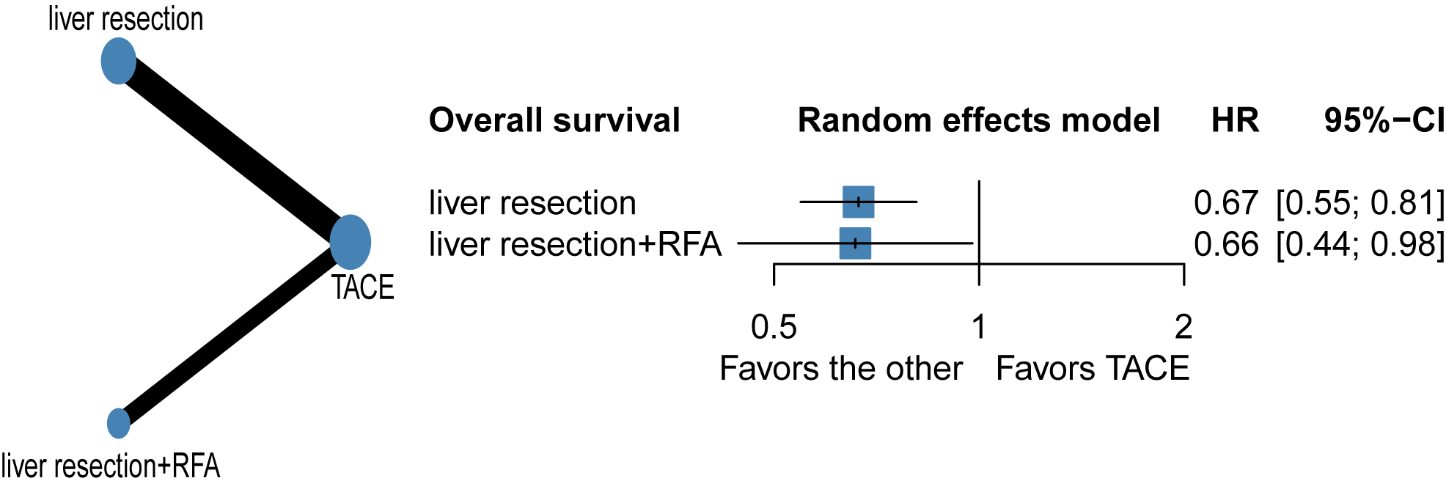
Figure S15 Risk of overall survival in studies with a lower percentage of HCV-infected patients (n=7 studies)**

**Table S18 Risk of overall survival in studies with a lower percentage of HCV-infected patients (n=7 studies)**

| **liver resection** | NA | 0.67 (0.55-0.81) |
| --- | --- | --- |
| 1.01 (0.65-1.57) | **liver resection+RFA** | 0.66 (0.44-0.98) |
| 0.67 (0.55-0.81) | 0.66 (0.44-0.98) | **TACE** |

**
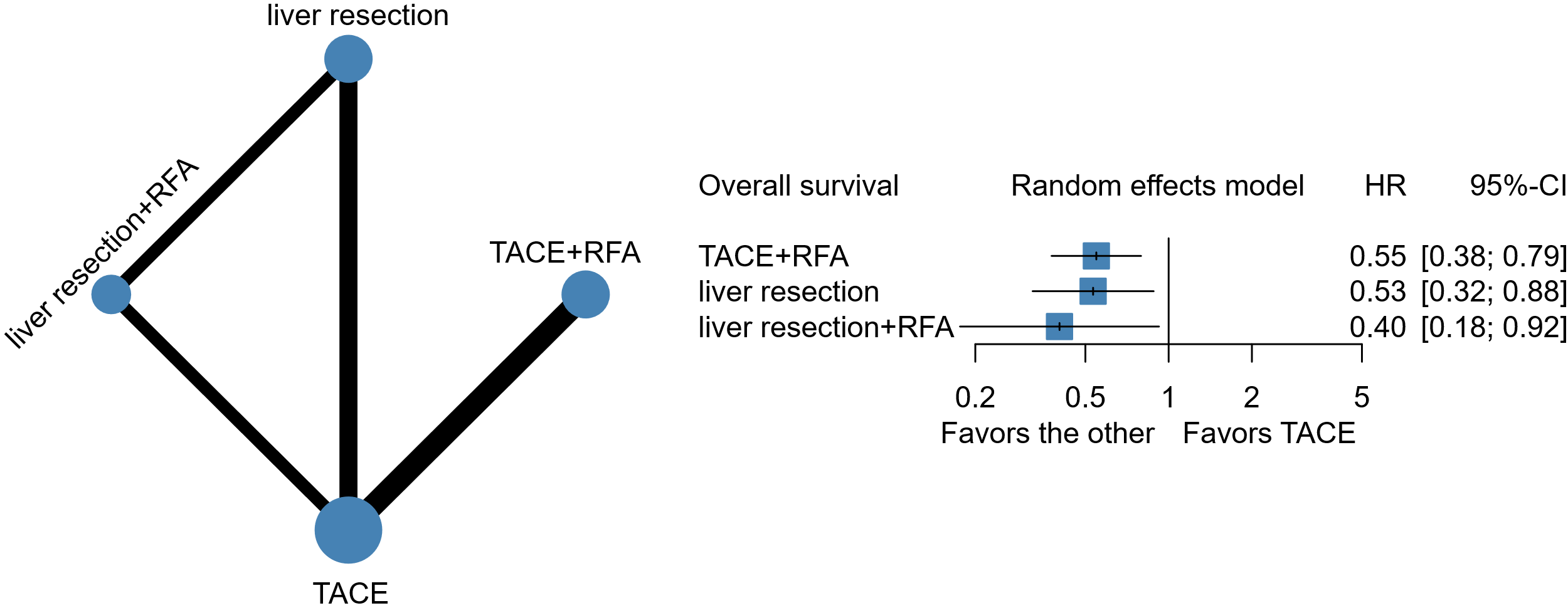
Figure S16 Risk of overall survival in studies with a higher percentage of HCV-infected patients (n=7 studies)**

**Table S18 Risk of overall survival in studies with a higher percentage of HCV-infected patients (n=7 studies)**

| **liver resection** | 1.09 (0.39- 3.03) | 0.56 (0.33- 0.96) | NA |
| --- | --- | --- | --- |
| 1.32 (0.59-2.96) | **liver resection+RFA** | 0.31 (0.09- 1.02) | NA |
| 0.53 (0.32-0.88) | 0.40 (0.18-0.92) | **TACE** | 1.83 (1.26- 2.65) |
| 0.97 (0.52-1.82) | 0.74 (0.30-1.83) | 1.83 (1.26-2.65) | **TACE+RFA** |

6. Tumor number

Tumor number ≥3 (the percentage of patients with tumor number ≥3)

**
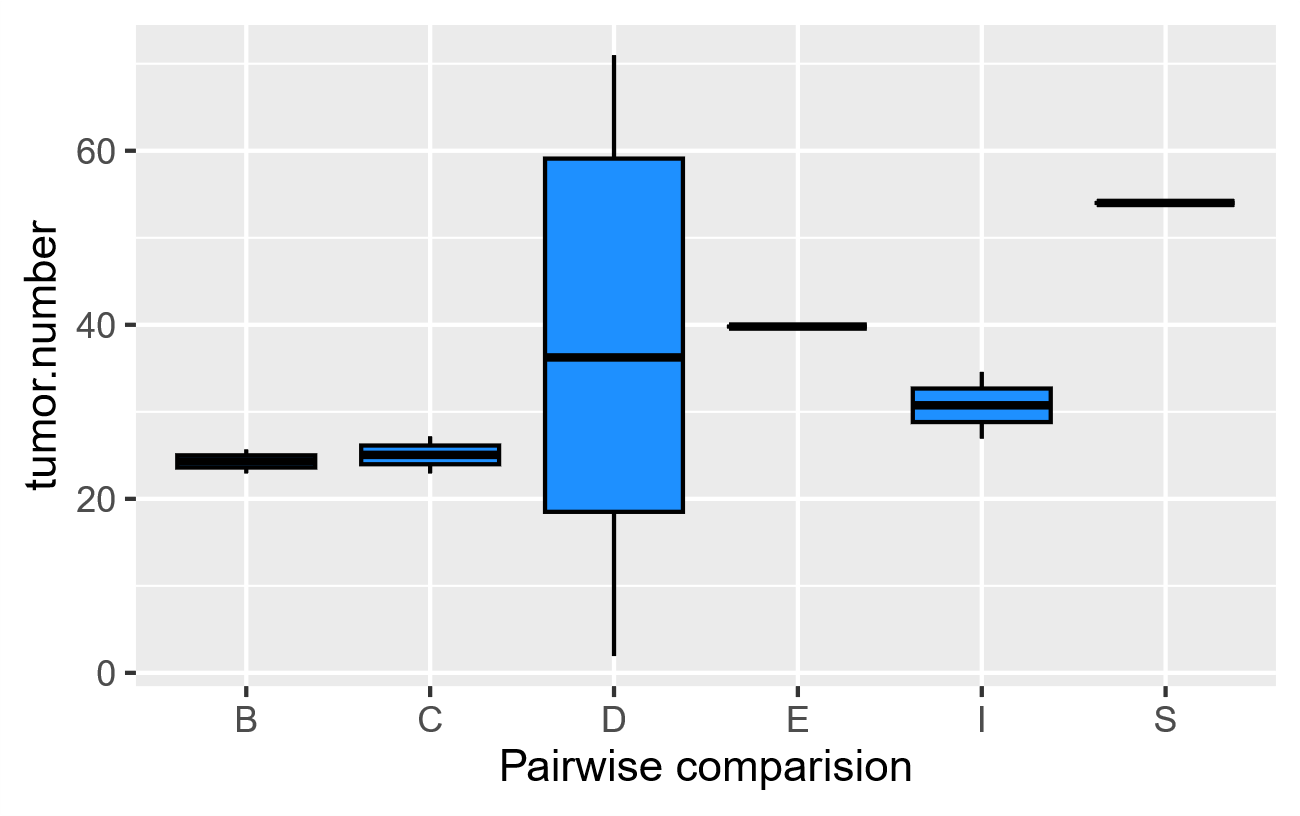
Figure S16 Distribution of the percentage of patients with tumor number ≥3**

Median of the percentage of patients with tumor number ≥3: 35% (IQR=23-NA)

**
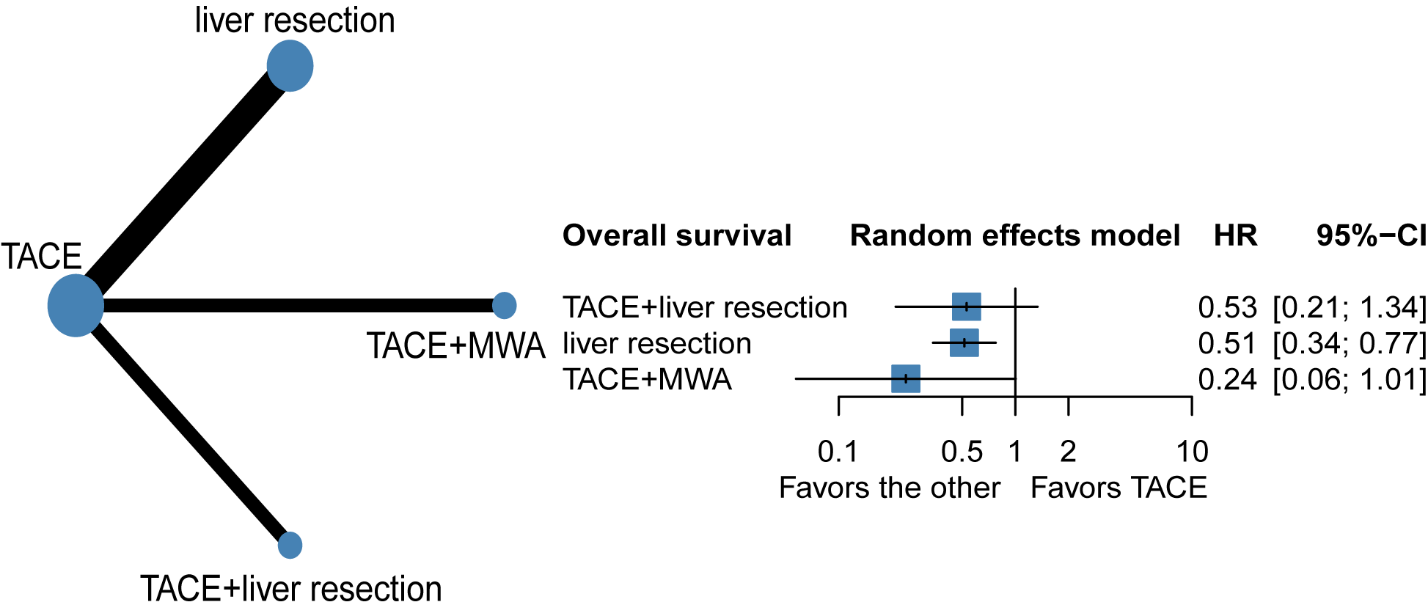
Figure S17 Risk of overall survival in studies with a higher percentage of tumor numbers ≥ 3 (n=6 studies)**

**Table S19 Risk of overall survival in studies with a higher percentage of tumor number ≥3 (n=6 studies)**

| **liver resection** | 0.51 (0.34- 0.77) | NA | NA |
| --- | --- | --- | --- |
| 0.51 (0.34- 0.77) | **TACE** | 1.89 (0.75- 4.76) | 4.17 (0.99-17.45) |
| 0.97 (0.35- 2.67) | 1.89 (0.75- 4.76) | **TACE+liver resection** | NA |
| 2.14 (0.48- 9.50) | 4.17 (0.99-17.45) | 2.21 (0.40-12.15) | **TACE+MWA** |

**Figure S18 Risk of overall survival in studies with a lower percentage of tumor number ≥3 (n=6 studies)**


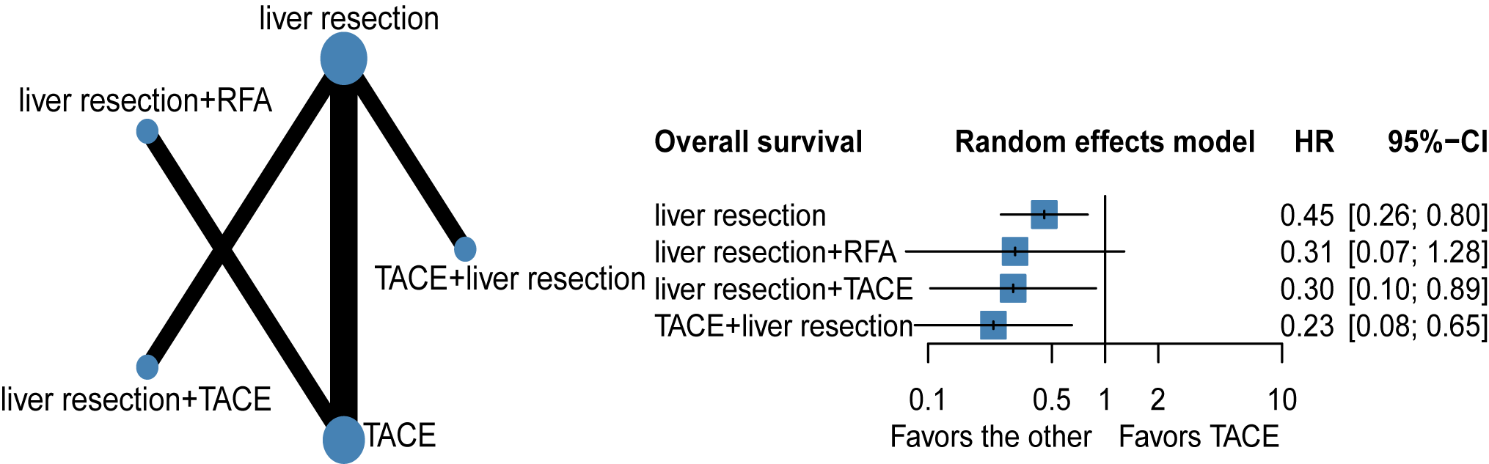


**Table S20 Risk of overall survival in studies with a lower percentage of tumor number ≥3 (n=6 studies)**

| **liver resection** | NA | 1.50 (0.60- 3.76) | 0.45 (0.26- 0.80) | 1.94 (0.83- 4.55) |
| --- | --- | --- | --- | --- |
| 1.46 (0.32- 6.74) | **liver resection+RFA** | NA | 0.31 (0.07- 1.28) | NA |
| 1.50 (0.60- 3.76) | 1.03 (0.17- 6.10) | **liver resection+TACE** | NA | NA |
| 0.45 (0.26- 0.80) | 0.31 (0.07- 1.28) | 0.30 (0.10- 0.89) | **TACE** | NA |
| 1.94 (0.83- 4.55) | 1.33 (0.23- 7.63) | 1.29 (0.37- 4.52) | 4.28 (1.54-11.89) | **TACE+liver resection** |

7. Tumor size (cm)

**
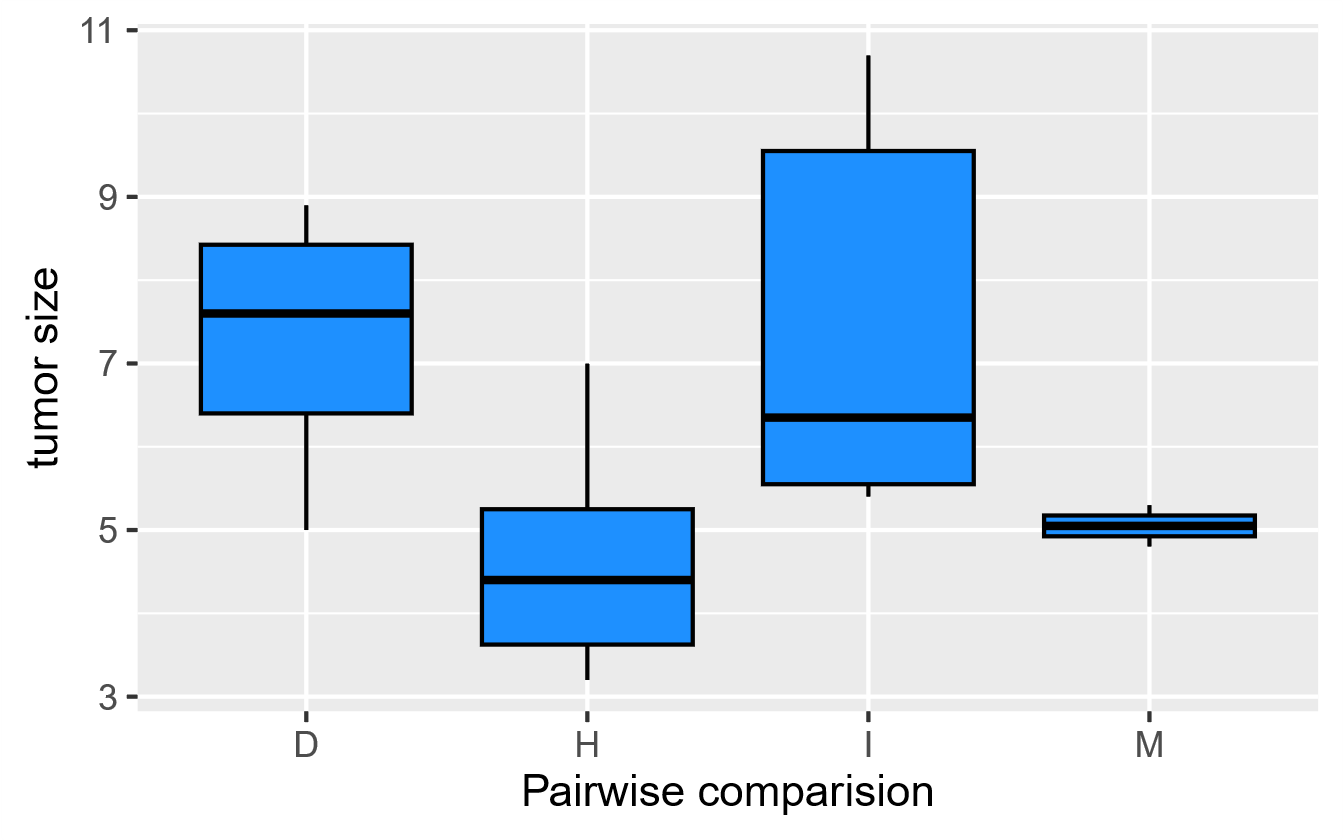
Figure S19: Distribution of the tumor size**

Median of tumor size: 6 (IQR=5-8)

**
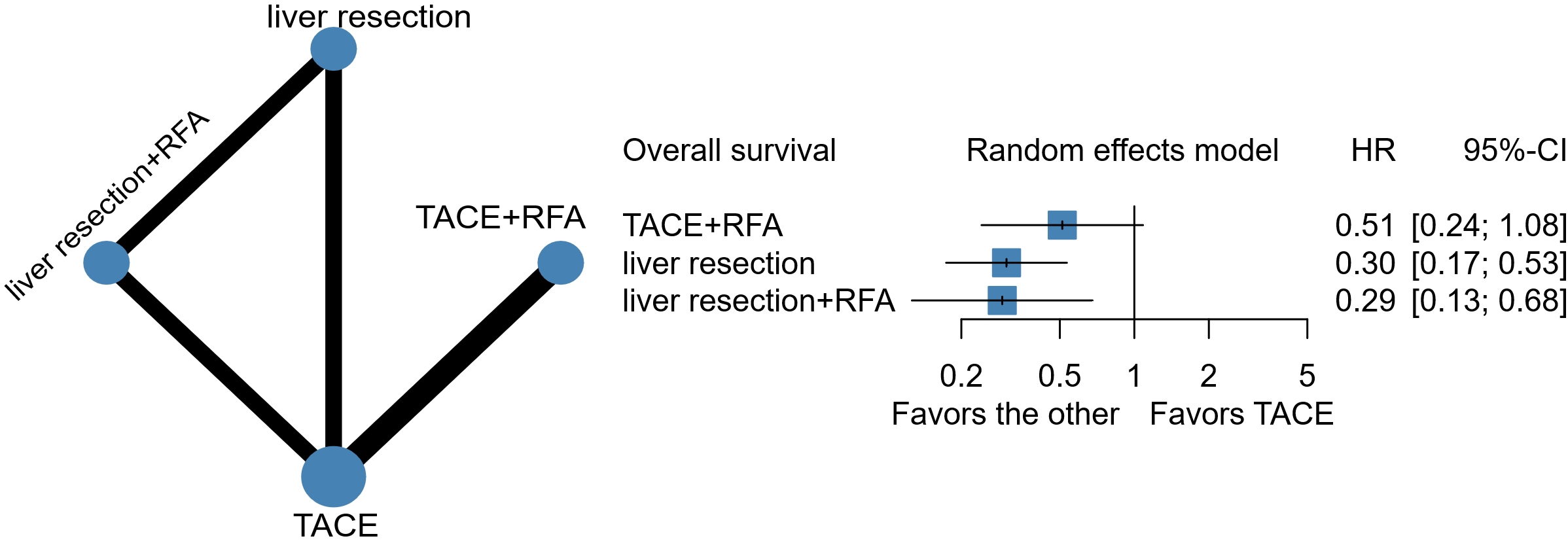
Figure S20 Risk of overall survival in studies with median tumor size ＜6 cm (n=5 studies)**

**Table S21 Risk of overall survival in studies with median tumor size ＜6 cm (n=5 studies)**

| **liver resection** | 1.09 (0.39- 3.03) | 0.30 (0.16- 0.55) | NA |
| --- | --- | --- | --- |
| 1.04 (0.46-2.35) | **liver resection+RFA** | 0.31 (0.09- 1.02) | NA |
| 0.30 (0.17-0.53) | 0.29 (0.13-0.68) | **TACE** | 1.96 (0.92- 4.14) |
| 0.60 (0.23-1.52) | 0.57 (0.19-1.77) | 1.96 (0.92-4.14) | **TACE+RFA** |

**
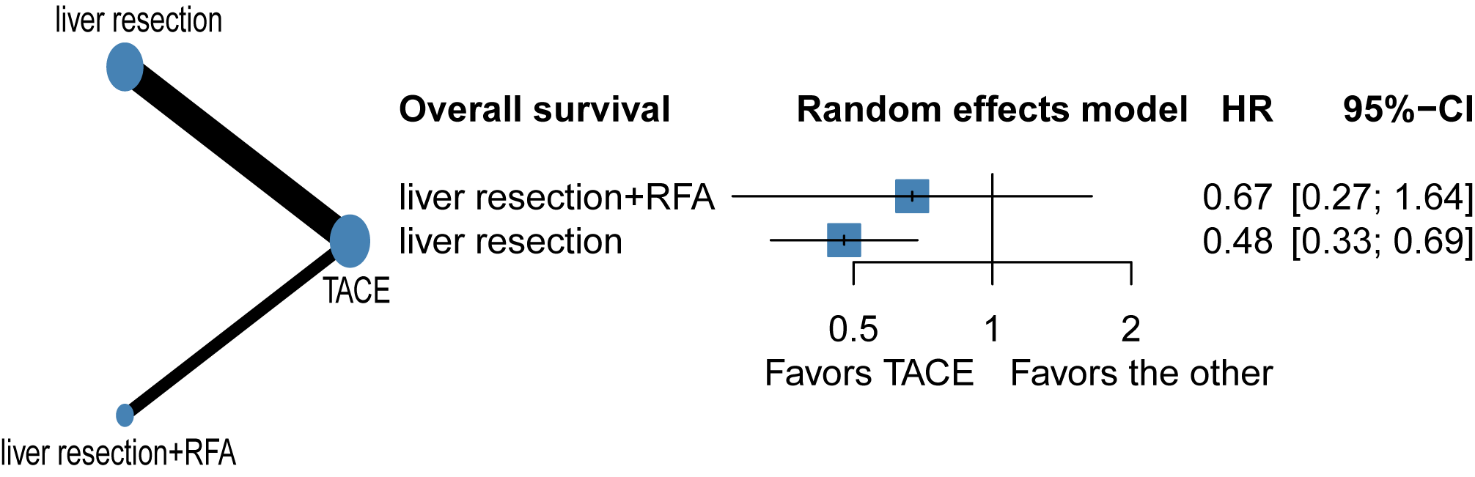
Figure S21 Risk of overall survival in studies with median tumor size ≥ 6 cm (n= 5 studies)**

**Table S22 Risk of overall survival in studies with median tumor size ≥ 6 cm (n=5 studies)**

| **liver resection** | NA | 0.48 (0.33-0.69) |
| --- | --- | --- |
| 0.71 (0.27-1.87) | **liver resection+RFA** | 0.67 (0.27-1.64) |
| 0.48 (0.33-0.69) | 0.67 (0.27-1.64) | **TACE** |

8. Sample size

**
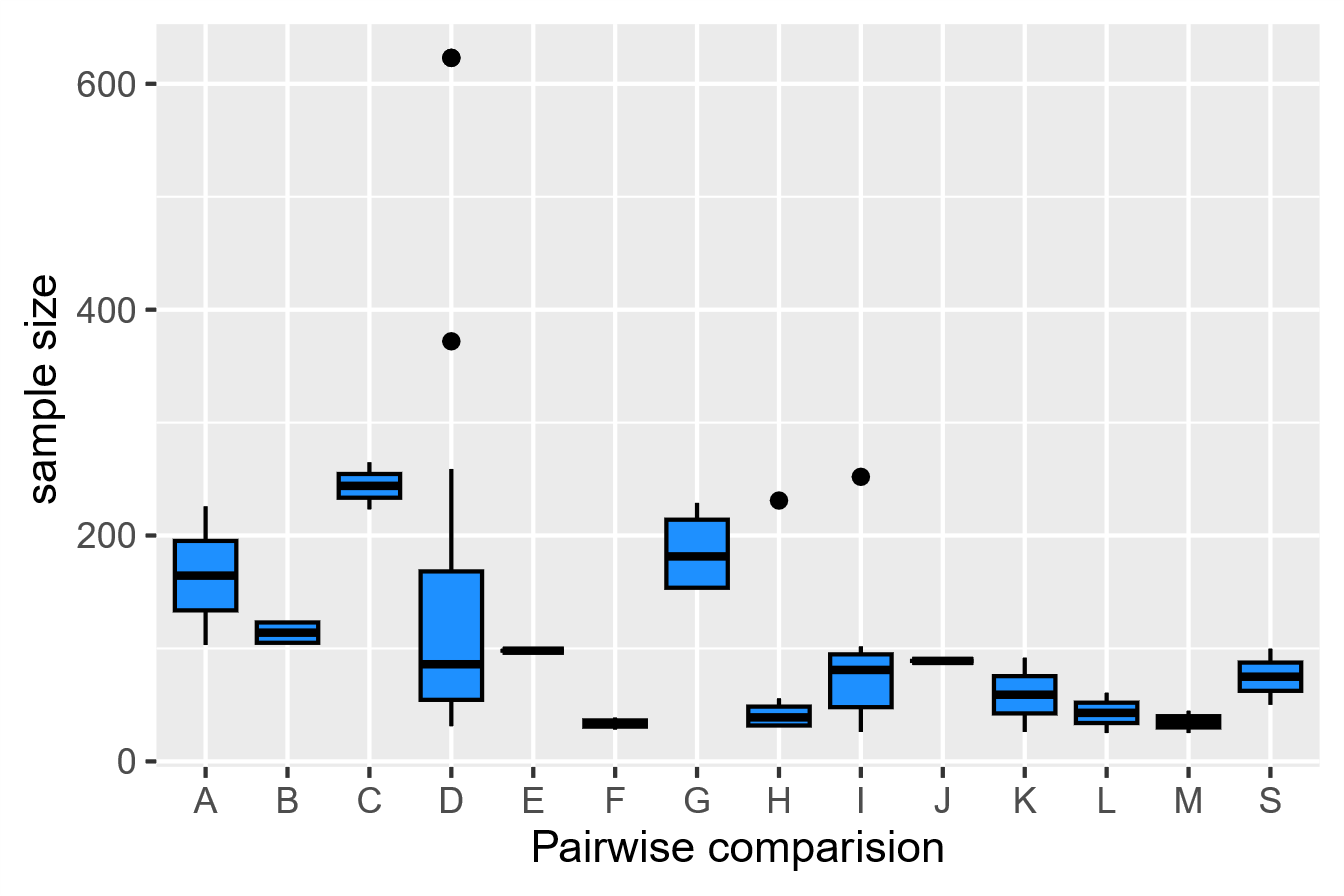
Figure S22: Distribution of the sample size of included studies**

Median of sample size: 88 (IQR=47-134)

**
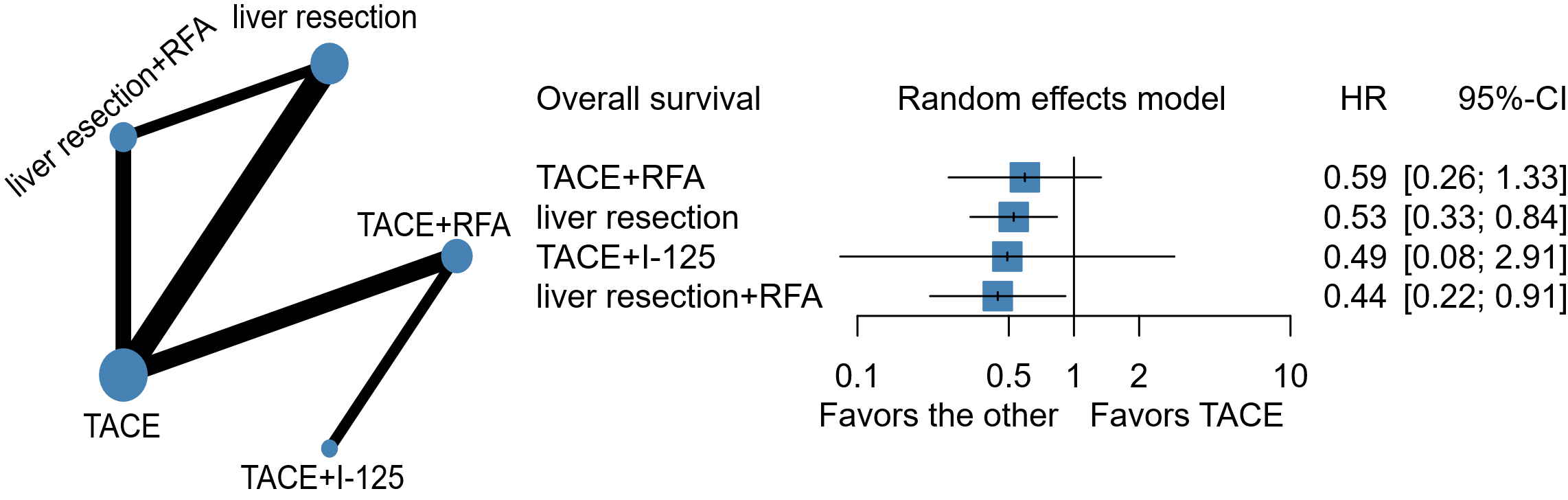
Figure S23 Risk of overall survival in studies with median sample size ＜88 (n = 13 studies)**

**Table S23 Risk of overall survival in studies with median sample size ＜88 (n = 13 studies)**

| **liver resection** | 1.09 (0.30-3.98) | 0.53 (0.33-0.86) | NA | NA |
| --- | --- | --- | --- | --- |
| 1.18 (0.54- 2.58) | **liver resection+RFA** | 0.43 (0.18-1.00) | NA | NA |
| 0.53 (0.33- 0.84) | 0.44 (0.22- 0.91) | **TACE** | NA | 1.69 (0.75-3.79) |
| 1.07 (0.17- 6.71) | 0.90 (0.13- 6.14) | 2.03 (0.34-12.01) | **TACE+I-125** | 0.83 (0.17-4.03) |
| 0.89 (0.35- 2.26) | 0.75 (0.25- 2.22) | 1.69 (0.75- 3.79) | 0.83 (0.17- 4.03) | **TACE+RFA** |

**
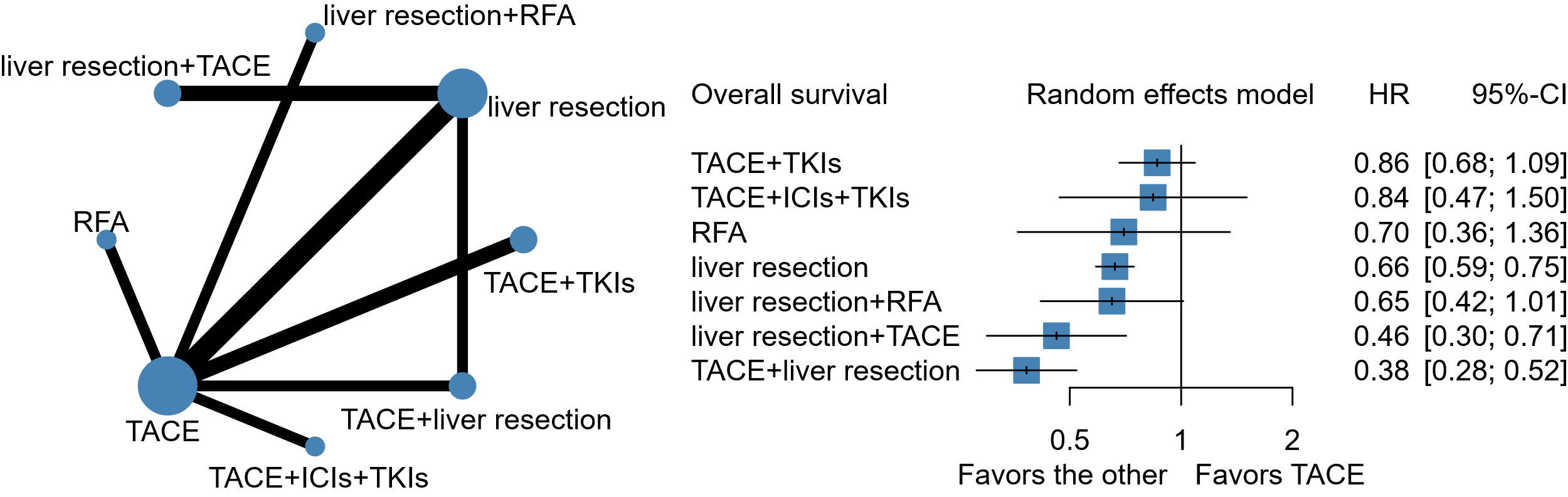
Figure S24: Risk of overall survival in studies with median sample size ≥88 (n = 13 studies)**

**Table S24 Risk of overall survival in studies with median sample size ≥88 (n = 13 studies)**

| **liver resection** | NA | 1.44 (0.95-2.18) | NA | 0.65 (0.58-0.74) | NA | 1.94 (1.37-2.74) | NA |
| --- | --- | --- | --- | --- | --- | --- | --- |
| 1.02 (0.64-1.61) | **liver resection+RFA** | NA | NA | 0.65 (0.42-1.01) | NA | NA | NA |
| 1.44 (0.95-2.18) | 1.41 (0.76-2.63) | **liver resection+TACE** | NA | NA | NA | NA | NA |
| 0.95 (0.48-1.85) | 0.93 (0.42-2.06) | 0.66 (0.30-1.45) | **RFA** | 0.70 (0.36-1.36) | NA | NA | NA |
| 0.66 (0.59-0.75) | 0.65 (0.42-1.01) | 0.46 (0.30-0.71) | 0.70 (0.36-1.36) | **TACE** | 1.19 (0.66-2.13) | 1.89 (1.05-3.41) | 1.16 (0.92-1.47) |
| 0.79 (0.43-1.43) | 0.77 (0.37-1.61) | 0.55 (0.26-1.13) | 0.83 (0.35-2.01) | 1.19 (0.66-2.13) | **TACE+ICIs+TKIs** | NA | NA |
| 1.73 (1.28-2.34) | 1.70 (0.99-2.93) | 1.20 (0.72-2.01) | 1.83 (0.88-3.80) | 2.62 (1.92-3.57) | 2.20 (1.14-4.26) | **TACE+liver resection** | NA |
| 0.77 (0.59-1.00) | 0.75 (0.46-1.25) | 0.53 (0.33-0.88) | 0.81 (0.40-1.64) | 1.16 (0.92-1.47) | 0.98 (0.52-1.83) | 0.44 (0.30-0.66) | **TACE+TKIs** |
